# Towards Heteroleptic Dicoordinate Cu^II^ Complexes

**DOI:** 10.1002/chem.202100888

**Published:** 2021-05-02

**Authors:** Michelle Kaiser, Jörg Göttlicher, Tonya Vitova, Alexander Hinz

**Affiliations:** ^1^ Karlsruher Institut für Technologie Institut für Anorganische Chemie (AOC) Engesserstrasse 15 76131 Karlsruhe Germany; ^2^ Karlsruher Institut für Technologie Institut für Photonenforschung und Synchrotronstrahlung (IPS) Hermann-von-Helmholtz-Platz 1 76344 Eggenstein-Leopoldshafen Germany; ^3^ Karlsruher Institut für Technologie Institut für Nukleare Entsorgung (INE) Hermann-von-Helmholtz-Platz 1 76344 Eggenstein-Leopoldshafen Germany

**Keywords:** copper, EPR, low-coordinate complexes, steric bulk, XANES

## Abstract

In this work we detail our efforts to systematically generate stable dicoordinate Cu^II^ complexes. Initial experiments via metathesis reactions of a bulky potassium carbazolide (RK) with copper(II) salts indeed yielded a stable product, RCuOTf (**1**). However, subsequent attempts to grasp systematic synthetic access to complexes of the type RCuX (X=monoanionic ligand) proved difficult as many of the complexes rapidly decomposed in solution. By using triflate‐related ligands such as ethyl sulfate and bistriflimide, the additional dicoordinate copper complexes RCuOSO_3_Et (**2**), [RCu(THF)][Cu(NTf_2_)_2_] (**3**) and RCuNTf_2_ (**4**) could be isolated. Spectroscopic indications corroborate more Cu^I^ than Cu^II^ character in all RCuX derivatives.

## Graphical abstract

**By employing a bulky carbazolyl substituent**, heteroleptic dicoordinate copper complexes with Cu^II^ character were synthesised. Delocalisation of the spin density in the ligand scaffold as well as dispersion forces apparently play a role in the stabilisation of these compounds.

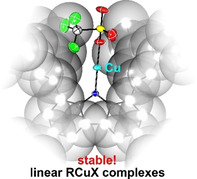

Recently, our group introduced a bulky substituent based on the carbazole scaffold and demonstrated its utility in the stabilisation of pseudo‐one‐coordinate tetrylenium ions of the type [R−E]^+^ (E=Ge, Sn, Pb; R=*N*‐1,8‐bis(3,5‐ditertbutylphenyl)‐3,6‐ditertbutylcarbazolyl).^[1]^ Our attention was then caught by the possibility of extending the scope of low‐coordinate species bearing the carbazolyl substituent to 3d metals, of which this contribution details our obtained results targeting dicoordinate Cu^II^ compounds.

Generally, transition metals tend to have higher coordination numbers than main group elements, but nevertheless low‐coordinate examples are known. Low‐coordinate (often three‐coordinate) complexes have been longstanding goals for synthetic work as they usually possess high reactivity as well as interesting properties.[^2^, ^3^, ^4^, ^5^, ^6^, ^7^] Notably, three‐coordinate Cu^II^ carbazolides were proposed by Fu and Jones as reactive intermediates in photoinduced Ullmann C−N coupling reactions which were observed by EPR spectroscopy after irradiation of Cu^I^ carbazolides in the presence of iodobenzene.^[5]^ However, all attempts to isolate this Cu^II^ carbazolide failed as it rapidly decomposed in solution.

For Cu^I^, dicoordination is one of the frequent coordination modes, for instance observed in isolated Gilman cuprate type complexes such as [Li(12‐crown‐4)_2_][CuMe_2_].^[6]^ In contrast, Cu^II^ usually adopts higher coordination numbers. Since pioneering work by Wannagat, Lappert and Power,^[7]^ a common bulky amido ligand in 3d metal chemistry has been ‐N(SiMe_3_)_2_,[^8^, ^9^, ^10^, ^11^, ^12^, ^13^, ^14^, ^15^, ^16^] but remarkably not even the typical divalent dicoordinate complex M(N{SiMe_3_}_2_)_2_ (Scheme 1, A) is known in substance for M=Cu^II^.^[17]^ However, because of the development of suitable sterically demanding ligands Power predicted in 2012 that it could be possible “that two coordination can be extended to divalent derivatives of the remaining first row elements scandium, titanium, vanadium, and copper with use of suitably large ligands“.^[18]^ Such complexes are interesting targets for synthesis due to their expectedly high reactivity. One of the desired complexes was then indeed discovered by the group of Power in 2016 (Scheme 1, **B**) utilising the Wigley ligand −N(SiMe_3_)Dipp (R’, Dipp=2,6‐diisopropylphenyl).^[19]^ The homoleptic complex featured two of these bulky amido ligands. Because many attempts to generate such complexes by salt metathesis routes were not successful,^[20]^ the Power group devised a unique way by exploiting the disproportionation of a Cu^I^ amido compound R'Cu into elemental copper and the desired Cu^II^ complex R’_2_Cu. As a possible driving force for this unusual reaction dispersion forces that stabilise the product were cited. While the dicoordinate Cu complex was stable in the solid state, in hydrocarbon solution at ambient temperature its decomposition was observed within hours. In stronger donor solvents such as toluene or ethers, no evidence for the presence of a Cu^II^ compound could be obtained.

The Power group reported on another attempt to generate dicoordinate Cu^II^ complexes in 2019 when they employed modified variants of the aryl(silyl)amide of the 2016 study.^[21]^ However, while for both Cu{N(Si^*i*^Pr_3_)_2_Dipp}_2_ (**C**) and Cu{N(Si^*i*^Pr_3_)Dipp‐4‐Ad}_2_ (**D**) unambiguous EPR spectra were obtained and the characteristic blue colour of the dicoordinate Cu^II^ complexes was observed, the isolation of the complexes was not possible due to their thermal instability.

As this is a complete survey of known dicoordinate Cu^II^ complexes, it is clear that this is a poorly understood problem of copper chemistry. We wish to contribute to solving this problem by exploring a metathesis route to obtain heteroleptic linear RCuX complexes.

**Scheme 1:**
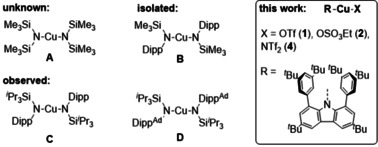
Dicoordinate Cu^II^ compounds (Dipp=2,6‐diisopropylphenyl, Dipp^Ad^=4‐adamantyl‐Dipp).

As a general method we chose the metathetical approach of treating potassium carbazolide with homoleptic Cu^II^ salts CuX_2_ (isolated or generated *in situ)*. In the initial set of experiments, simple cupric halides and pseudohalides were employed such as X=Cl, Br, OTf. Out of these candidates, only the triflate derivative RCuOTf (**1**) was found to be stable and could be isolated in good yield of 77 % (Scheme 2). The stability of this compound, however, is surprising compared to Power's complex as it is stable not only at ambient temperature but also in aprotic solvents such as THF, Et_2_O or toluene.

**Scheme 2:**
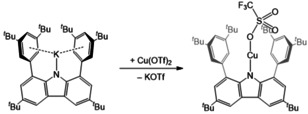
Synthesis of the heteroleptic linear RCuOTf complex (**1**).

The complex RCuOTf (**1**) features an N−Cu−O angle of 177.31(5)° with Cu−N and Cu−O distances of 1.8585(11) and Cu−O 1.8669(14) Å, respectively (Figure 1). These values are closely related to the structural features in Power's complex **B** (Cu1−N1 1.7914(10) Å, N1−Cu1−N1 A 180°). As formally, there is a d^9^ electron configuration, the compound is expected to be EPR active. Indeed, at ambient temperature an EPR resonance was observed at g=2.01063. The signal exclusively displayed hyperfine coupling with Cu and N (A_iso_(^63^Cu) 74.23, A_iso_(^14^N) 21.03 MHz). As N predominantly is ^14^N with I=1 and Cu has two I=3/2 nuclei, ^63^Cu and ^65^Cu, there is a principal three‐line splitting due to the ^14^N and a four‐line splitting due to the Cu atoms, where the difference in gyromagnetic ratios is observable only in the outer lines of the signal (Figure 2). The values compare very well with the computed values for RCuOTf (g 2.0189, A_iso_(^63^Cu) 82.95, A_iso_(^14^N) 26.36 MHz, computed with Gaussian16, M062X/def2TZVP), but are smaller than in Power's homoleptic bis‐amido complexes (A_iso_(^63^Cu) 125.82, A_iso_(^14^N) not resolved, see Supporting Information 2.6), indicating more spin density delocalised in the carbazole framework than in conventional amido substituents. The deep purple compound featured two strong absorption bands in the visible region of the electronic spectrum (787, 550 nm). A third band as also observed in Power's complex would be expected in the low‐energy range of the spectrum was not resolved.

**Figure 1:**
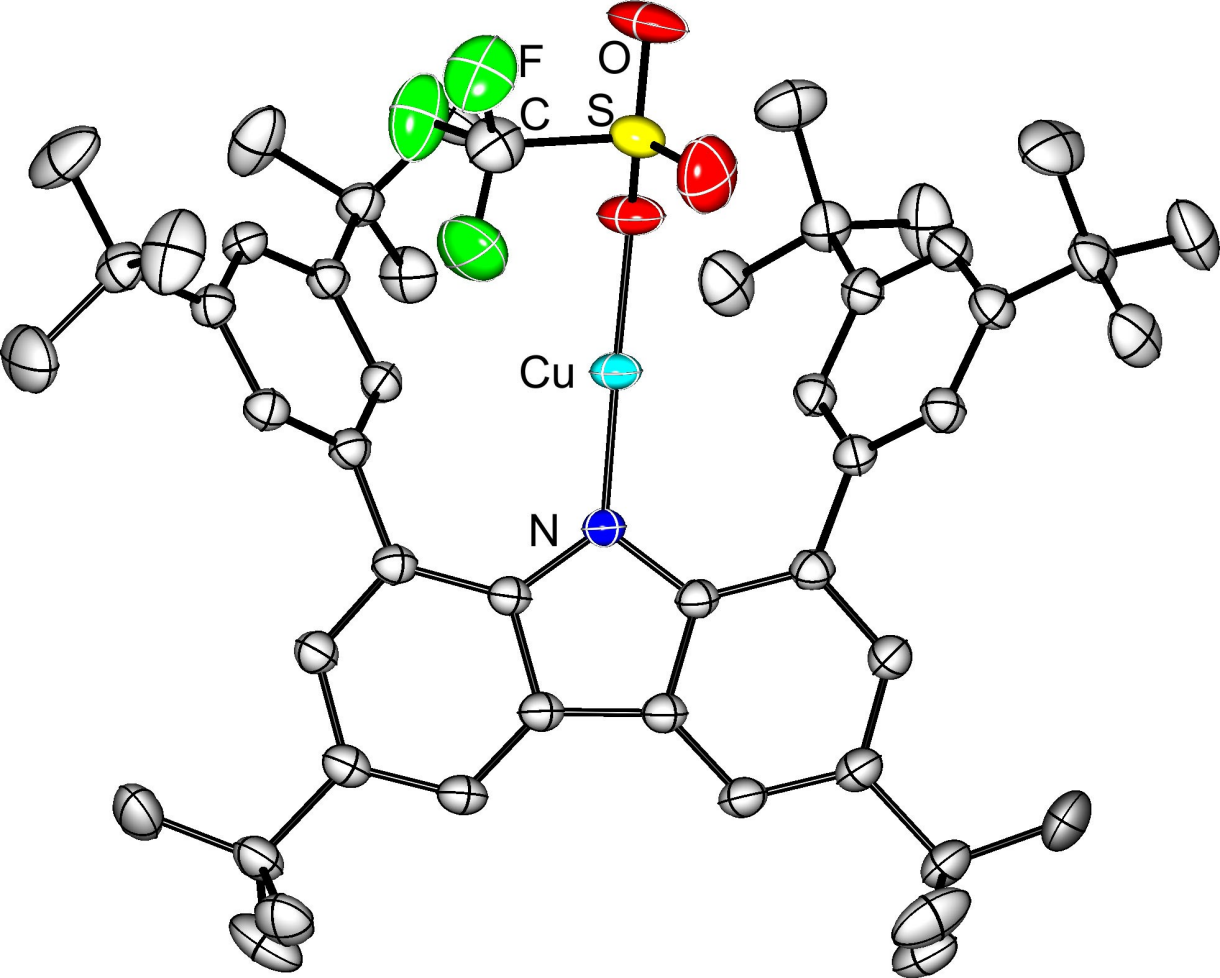
Molecular structure of RCuOTf (**1**). Ellipsoids for Heteroatoms are set at 50 % probability at 200 K. Selected distances [Å] and angles [°]: N‐Cu 1.8585(11), Cu‐O 1.8669(14), N‐Cu‐O 177.31(5).

**Figure 2:**
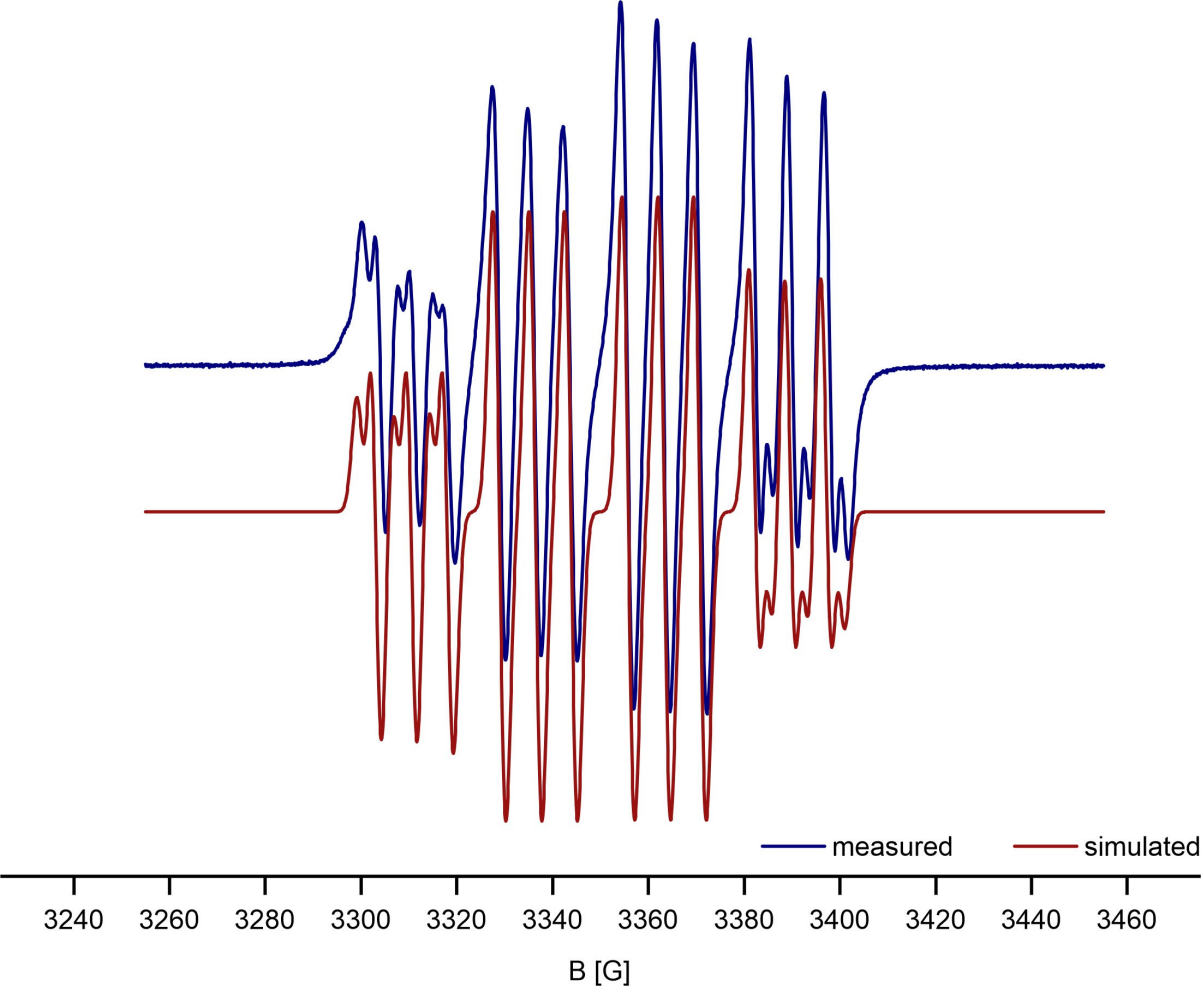
EPR spectrum of RCuOTf (**1**) in hexane solution at ambient temperature (measured top, simulated bottom).

With these data in hand we attempted to find similar compounds to establish a systematic trait that allows for the prediction of stable dicoordinate Cu^II^ compounds. Dispersion forces were recognised as important factor in the stabilisation of the homoleptic complex R’_2_Cu (**B**). However, we did not expect comparable results for our system, as there is just one bulky substituent providing most of the dispersion interaction with itself, and that to a larger extent than in the Power system. The dispersion interaction between R and the counterion could be estimated by the difference between the dispersion stabilisation in [RCu]^+^ and RCuX (Supporting Information, Table S10).

What we thought to be more telling was the computed spin density on the Cu and N atom of the R−Cu fragment. Both Power's complexes show relatively low spin density at the Cu atom (**B** 0.256, **C** 0.228 e) but high contributions of N spin density (**B** 0.356, **C** 0.356 e). A similar situation is found in RCuOTf (Cu 0.146, N 0.362 e). Our working hypothesis then turned to minimising spin density on Cu to enhance stability of the resulting complex (see Supporting Information 4.3). Among the considered anionic ligands in RCuX there were the alkoxides O^*t*^Bu, O^*t*^Bu^F^, the amide N(SiMe_3_)_2_, pseudohalides N_3_, O_2_C_2_F_3_, and the ions related to OTf, NTf_2_, OSO_3_Et and OTos. Various data from our analysis are compiled in Table 1.

**Table 1:** Computed data for the Cu^II^ complexes (stable in bold).^[a]^

Compound	Spin density on Cu [e]	Spin density on N [e]	G‐G_disp_ [kJ mol^−1^]^[b]^
[RCu]^+^	0.013	0.408	263.8
**[RCu(THF)]^+^**	**0.051**	**0.396**	**304.8**
RCuSbF_6_^[c]^	0.090	0.400	300.5
**RCuNTf_2_**	**0.109**	**0.392**	**340.5**
RCuBF_4_^[c]^	0.145	0.396	282.3
**RCuOTf**	**0.146**	**0.362**	**303.5**
**RCuOSO_3_Et**	**0.165**	**0.355**	**310.4**
RCuN_3_	0.172	0.321	273.8
RCuO^*t*^Bu^F^	0.178	0.349	322.0
RCuCl	0.183	0.363	271.8
RCuOTos	0.190	0.338	328.6
RCuOAc^F^	0.230	0.325	290.7
RCuN(SiMe_3_)_2_	0.259	0.304	341.6
RCuO^*t*^Bu	0.350	0.217	312.6
Cu(N{Si^*i*^Pr}_3_Dipp)_2_	0.228	0.356	341.8
**Cu(N{SiMe_3_}Dipp)_2_**	**0.256**	**0.356**	**222.7**
Cu(N{SiMe_3_}_2_)_2_	0.305	0.318	108.1
Cu(NTf_2_)_2_	0.629	0.074	77.7

[a] Gaussian16, PBE1PBE/Def2SVP, no solvent corrections; [b] ΔG between optimised structures with and without inclusion of dispersion forces; [c] tricoordinate complexes with two similar Cu−F contacts.

Several candidates were promising for comparable electronic structure, but RCuSbF_6_ and RCuBF_4_ were excluded because the predicted products are three‐coordinated. Some of the other examples turned out yield unstable products, but radical species could be observed by EPR spectroscopy (see below). However, two of these attempts showed success.

In case metathesis reaction of RK with Cu(OSO_3_Et)_2_ in THF the expected purple colour of RCuOSO_3_Et (**2**) was observed. Even after numerous attempts, no crystals suitable for XRD experiments could be obtained, but both the EPR and UV/Vis spectra show the expected signals for the linear copper complex (Table 2). Similarly, the metathesis reaction of RK with Cu(NTf_2_)_2_ in THF yielded a purple solution that produced a Cu^II^‐related EPR resonance (Table 2). However, single‐crystal structure elucidation revealed the formation of [RCu(THF)][Cu(NTf_2_)_2_]. In the solid state structure, two molecular entities, [RCu(THF)] and [Cu(NTf_2_)_2_], are found (Figure 3). Two possibilities arise: Either [RCu(THF)][Cu(NTf_2_)_2_] is a co‐crystallisate of two neutral molecules, or [RCu(THF)]^+^[Cu(NTf_2_)_2_]^−^ is a salt with two complex ions. We propose, that the oxidation states should be assigned in the latter fashion as [RCu(THF)]^+^[Cu(NTf_2_)_2_]^−^ on the basis of the following argumentation.

**Table 2:** EPR and UV/Vis data for linear Cu^II^ complexes **1–4**.

Compound	**RCuOTf 1**	**RCuOSO_3_Et 2**	**[RCuTHF] [Cu(NTf_2_)_2_] 3**	**RCuNTf_2_ 4**
g_iso_	2.01063	2.01750	2.01076	2.01408
A_iso_(^63^Cu) [MHz]	74.23	70.96	71.27	71.20
A_iso_(^14^N) [MHz]	21.03	21.99	22.45	22.51
λ_max_ [nm]	787, 550	783, 578	756, 513	772, 510

**Figure 3:**
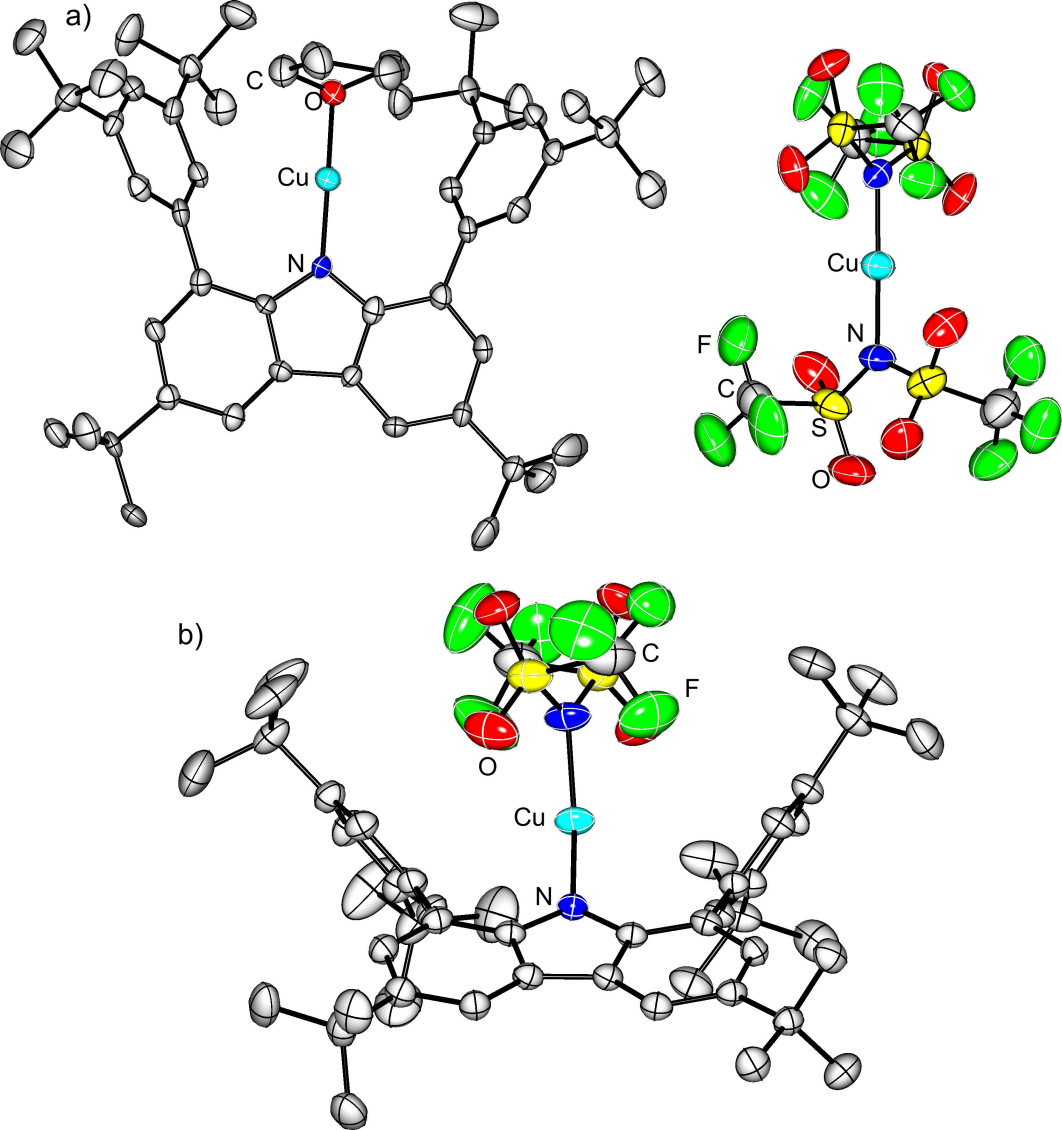
Molecular structure of a) [RCu(THF)][Cu(NTf_2_)_2_] (**3**, 150 K) and b) RCuNTf_2_ (**4**, 200 K). Ellipsoids are set at 50 % probability.

The computed molecular structures of [RCu(THF)] and [RCu(THF)]^+^ differ only slightly, so no strong case for either option can be made (Supporting Information 4.2.. Determined by XRD experiments, the N−Cu−O angle is slightly bent (170.3(3)°) and the N−Cu (1.845(6) Å) and Cu−O (1.873(5) Å) bonds are of comparable length. However, the computed structures of [Cu(NTf_2_)_2_]^−^ and [Cu(NTf_2_)_2_] differ greatly. The coordination geometry of [Cu^II^(NTf_2_)_2_] is distorted square planar, with two short Cu−O contacts (2.04 Å) and an S−N−N−S dihedral angle is close to 180°. In contrast, for [Cu(NTf_2_)_2_]^−^ all Cu−O contacts exceed 3 Å and the dihedral angle is 90°. The latter geometry closely resembles the determined structure in the crystal (Cu−O 2.96‐3.02 Å, dihedral of 104°). A similar case can be made by comparison of the predicted EPR parameters for [RCu(THF)]^+^ and [Cu(NTf_2_)_2_]. While the latter is predicted to have considerable spin density of Cu and correspondingly, a g value differing significantly from 2 (Table 1, Supporting Information 4.2.3), the former is predicted to have a g value close to 2. The experimental spectrum featured a resonance at g=2.01076, which also corroborates the formulation of **3** as [RCu(THF)]^+^[Cu(NTf_2_)_2_]^−^. This is also in line with the initial hypothesis that for linear complexes high spin density on Cu should correlate with low stability.

Obviously, the solvent THF has an impact on the formation of **3**, so the experiment was repeated in a less coordinating solvent, hexane, and indeed the desired heteroleptic complex RCuNTf_2_ (**4**, 64 %) was obtained as purple crystalline material. The EPR and UV/Vis spectra of **4** are very similar to the ones found for **1**, **2** and **3** (Table 2). Structurally, **4** is characterised by ^R^N−Cu and Cu−N^Tf^ distances of 1.864(2) and 1.926(3) Å, respectively, as well as an angle NCuN of 155.29(11)° which deviates considerably from an ideal linear arrangement due to steric repulsion of the NTf_2_ ligand with the flanking arenes.

Numerous other CuX_2_ complexes were employed in attempts of metathesis, however, no Cu^II^ containing products could be isolated. In case of attempted syntheses of RCuO^*t*^Bu, RCuOTos and RCuOAc^F^, radical intermediates could be observed, which did not show hyperfine coupling to Cu but only ^14^N hyperfine splitting (O^*t*^Bu: g 2.0120, A_iso_(^14^N) 39.73 MHz; OTos: g 1.9804, A_iso_(^14^N) 61.07 MHz; OAc^F^: g 2.0016, A_iso_(^14^N) 44.29 MHz; Supporting Information 2.8). This indicates homolysis of the N−Cu bond with the formation of a carbazolyl radical and a Cu^I^ complex as a major decomposition pathway.

It is surprising that *only* for the closely related OTf^−^, OSO_3_Et^−^ and NTf_2_^−^ anions, the linear copper complex is stable. This allows some degree of generalisation of the properties of the stable linear RCuX complexes. Most of the spin density resides on the [RCu] moiety of the complex, while the counterion X has little to no influence on the electronic situation of the [RCu]^+^ fragment.

An issue which necessarily needs to be addressed is whether the novel complexes should be considered Cu^II^ compounds with an anionic carbazolyl ligand [R^−^→Cu^2+^] or as Cu^I^ compounds with a neutral carbazolyl radical ligand [R→Cu^+^]. To shed light on this question, Cu−K edge XANES experiments were conducted as they conventionally allow the determination of oxidation states.^[22]^ For comparison, the complex [RCu(PPh_3_)] (**5**) which we would describe as Cu^I^ complex, as well as Power's Cu^II^ complex [{DippN(SiMe_3_)}_2_Cu] (**B**) were prepared. Linear coordination of the copper atom by produces a pronounced pre‐edge peak due to the dipole‐allowed 1s→4p transition. This is expected for Cu^I^ and Cu^II^ complexes equally (cf. dicoordinate Cu^I^ 8983.4–8984.2 eV, Cu^II^ with 2+3 or 2+4 coordination 8983.4‐8984.2 eV),^[23]^ but the energy of this transition may allow some inference for the oxidation state, as lower energies for this transition are indicative of more Cu^I^ character. The XANES spectra of the carbazolyl complexes **2**, **3**, and **4** (Figure 4, maximum at 8983.4 eV) bear semblance to the spectrum obtained for **B** (8983.4 eV). However, the Cu^I^ carbazolide **5** reveals its pre‐edge peak at 8981.8 eV with a clear‐cut difference to **1–4**. The energy of this transition for **1** (8982.7 eV) is higher than in the spectrum observed by the Peters group for tricoordinate complexes ([Ph_2_B(CH_2_P^*t*^Bu_2_)_2_Cu(NTol_2_)]^−^ 8982.2 eV, [Ph_2_B(CH_2_P^*t*^Bu_2_)_2_Cu(NTol_2_)] 8982.5 eV) as they referenced the first inflection point of the Cu foil spectrum to 8980.3 eV while our values have a 8979.0 eV reference.^[24]^ For Cu^II^ compounds there is typically a peak with very low intensity at below 8980 eV indicative of the 1s→3d transition^[25]^ which was not observed in the dicoordinate complexes.

**Figure 4:**
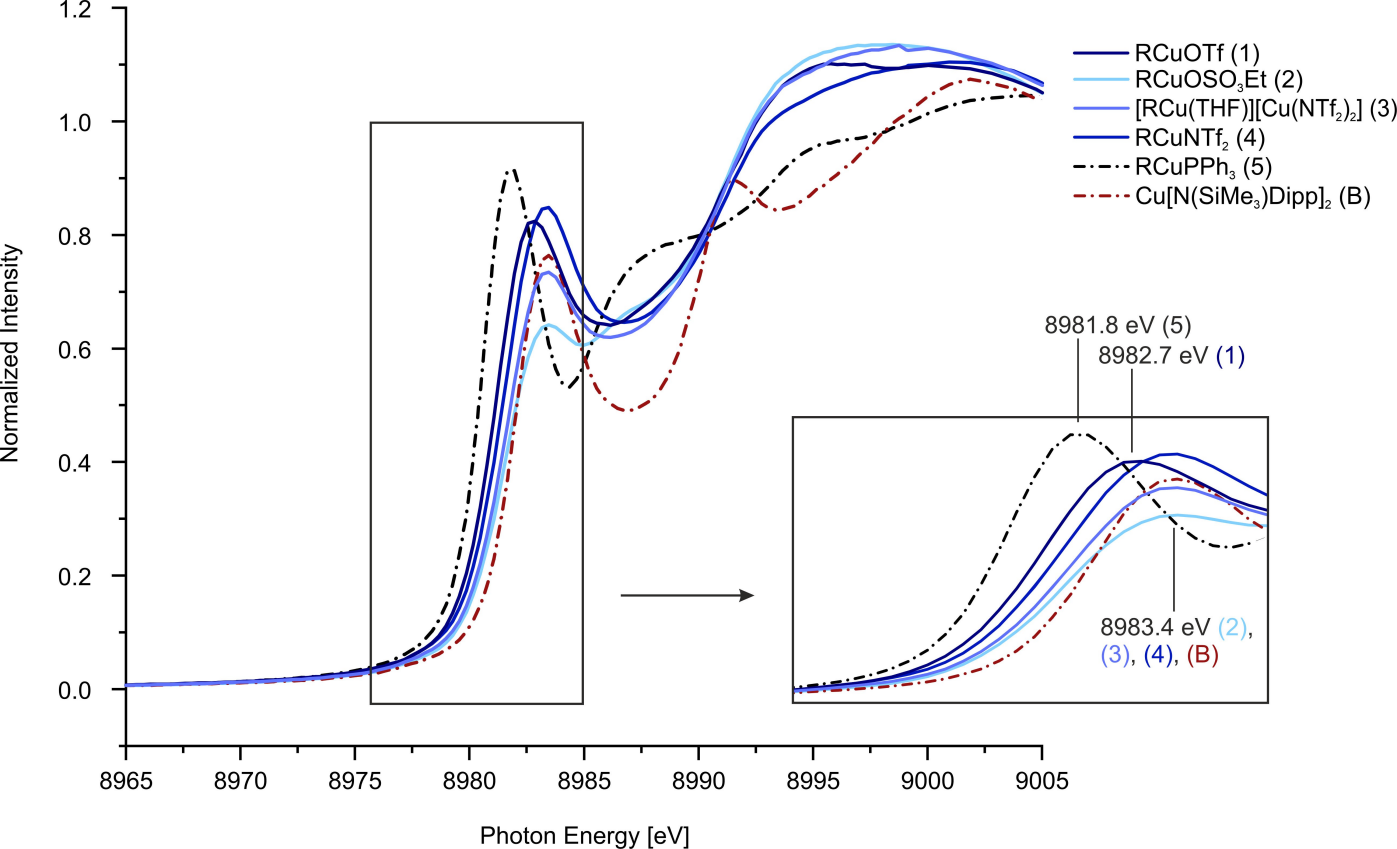
Cu−K‐edge XANES spectra of **1–5** and **B**.

The redox non‐innocence of carbazoles is well established. As early as 1972, Neugebauer and co‐workers isolated the neutral 1,3,6,8‐tetra‐tertbutyl‐9‐carbazolyl radical in substance^[26]^ and we encountered oxidation of the carbazole RH in an earlier study.^[1]^ These radicals are typically studied with EPR and UV/Vis spectroscopy as well as cyclic voltammetry. The electronic properties carbazolyl organoradicals can be finely tuned by introducing different substituents, usually exhibiting olive‐green to blue solutions with absorption maxima between 500 and 860 nm.^[27]^ For comparison, only [^*t*^Bu_4_CarbH]^+^, [RH]^+^, and [{DippN(SiMe_3_)}_2_Cu] (**B**) will be discussed. The UV/Vis spectrum of [^*t*^Bu_4_CarbH]^⋅+^ exhibited three maxima at 547, 583 and 927 nm.^[26]^ The bulky arene‐bearing carbazole RH could be oxidised to the corresponding turquoise radical cation and featured three absorptions in the UV/Vis range at 378, 470, and 770 nm, as well as another band at lower energy, beyond accessible scope of the used UV/Vis spectrometer. Power observed three transitions at 640, 880, and 960 nm for his purple compound [{DippN(SiMe_3_)}_2_Cu] (**B**), stating that these correspond to the differences between the calculated energies of the 3d orbitals. In the blue‐violet copper carbazolides, two strong absorptions were found in the range of 510–580 and 750–790 nm. According to TD‐DFT analysis of RCuOTf, these are dominated by SOMO‐8→SOMO and SOMO‐2/3→SOMO transitions, where all involved MOs have contributions of the carbazole π‐scaffold and Cu 3d‐orbitals.

Cyclic voltammetry of RCuOTf showed a reversible redox process at E_1/2_=−0.33 V vs Fc/Fc^+^ in THF. The value is related to Power's finding of a reversible Cu(I/II) oxidation at E_1/2_=−0.499 V for **B**. and is at higher potential than [Ph_2_B(CH_2_P^*t*^Bu_2_)_2_Cu(NTol_2_)] (E_1/2_=−0.882 V),^[24]^ but at lower potential than typical oxidations of carbazoles (RH +0.69 V).[^1^, ^28^]

EPR spectra in frozen solution commonly are strongly anisotropic and show large coupling tensor displays large hyperfine coupling in correlation with the highest g value. In **1–4**, the EPR spectra in frozen solution were too convoluted to reliably fit the data, but the anisotropy is smaller than in **B**.

The spin densities for formal Cu^II^ complexes obtained by XANES or DFT methods in the literature vary, ranging from (BDI)Cu(NHAd) (0.30e), B (0.26e), LCuNR (0.13e), blue copper site (0.41e). The new complexes **1–4** with the exception of [RCu(THF)]^+^ fall into the lower end of this range (0.11–0.17e).

In conclusion, we established a metathesis route towards heteroleptic carbazolyl copper complexes. In the products, the change in stability of RCuX in dependence of X is quite remarkable. The spin density on Cu and N in the computed RCuX complexes varies in the ranges of 0.05–0.35e and 0.22–0.41e, respectively. In this heteroleptic linear coordination environment it appears impossible to bestow more than 0.17e spin density on the Cu atom. Considering UV/Vis, EPR and Cu‐XANES spectra as well as DFT analysis and the surprising inherent instability of the other members of the RCuX family, the electronic situation in **1–4** is in between Cu^I^ and Cu^II^ and probably best described as mainly Cu^I^ with some Cu^II^ character. Further theoretical and practical work is required to develop deeper understanding of the stability of linear Cu^II^ compounds and to obtain genuine linear Cu^II^ complexes, other approaches have to be explored.

CCDC 2004478, 2004479, 2004480 and 2063690 contain the supplementary crystallographic data for this paper. These data are provided free of charge by The Cambridge Crystallographic Data Centre.

## Conflict of interest

The authors declare no conflict of interest.

## Supporting information

As a service to our authors and readers, this journal provides supporting information supplied by the authors. Such materials are peer reviewed and may be re‐organized for online delivery, but are not copy‐edited or typeset. Technical support issues arising from supporting information (other than missing files) should be addressed to the authors.

Supplementary
