# Supplementary material for: Towards Heteroleptic Dicoordinate CuII Complexes
Source: Chemistry. 2021 May 2;27(30):7998–8002. doi: 10.1002/chem.202100888 (PMC8252544; doi:10.1002/chem.202100888)
Supplement: Supplementary file 1 — Supplementary [file CHEM-27-7998-s001.pdf]

Chemistry–A European Journal

Supporting Information

Towards Heteroleptic Dicoordinate Cu^{II} Complexes

Michelle Kaiser, Jörg Göttlicher, Tonya Vitova, and Alexander Hinz*

Contents

1 General Considerations 1

2 Syntheses 3

2.1 RCuOTf (1) 3

2.2 $RCuOSO_3Et$ (2) 7

2.3 $[RCu(THF)][Cu(NTf_2)_2]$ (3) 10

2.4 $RCuNTf_2$ (4) 14

2.5 $RCu\text{-}PPh_3$ (5) 17

2.6 Radical Intermediates 19

2.6.1 RK + $Cu(O^tBu)_2$ 20

2.6.2 RK + $Cu(OTos)_2$ 21

2.6.3 RK + $Cu(OAc^F)_2$ 22

3 Crystallography 23

4 Computational details 24

4.1 Overview 24

4.2 $[RCu(THF)][Cu(NTf_2)_2]$ 26

4.2.1 [RCu(THF)] fragment 26

4.2.2 $[Cu(NTf_2)_2]$ fragment 26

4.2.3 EPR parameters 27

4.3 Spin density plots, isosurfaces at 0.005 28

4.3.1 RCuOTf 28

4.3.2 $RCuOSO_3Et$ 29

4.3.3 $[RCu(THF)]^+$ 30

4.3.4 $RCuNTf_2$ 31

4.4 Optimised Structures 32

4.4.1 RCuOTf 32

4.4.2 $[RCu]^+$ 38

4.4.3 $RCuSbF_6$ 40

4.4.4 $RCuNTf_2$ 42

4.4.5 $RCuBF_4$ 44

4.4.6 $RCuOSO_3Et$ 46

4.4.7 $RCuN_3$ 48
4.4.8 $RCuO^tBu^F$ 50
4.4.9 RCuCl 52
4.4.10 RCuOTos 54
4.4.11 $RCuO_2C_2F_3$ 56
4.4.12 $RCuN(SiMe_3)_2$ 58
4.4.13 $RCuO^tBu$ 60
4.4.14 $[RCu(THF)]^+$ 62
4.4.15 $Cu(NTf_2)_2^-$ 65
4.4.16 $Cu(NTf_2)_2$ 65
4.4.17 $Cu(N\{SiMe_3\}_2)_2$ 66
4.4.18 $Cu(N\{SiMe_3\}Dipp)_2$ 67
4.4.19 $Cu(N\{Si^iPr_3\}Dipp)_2$ 68
5 References 71

1 General Considerations

Et_2O (Sigma-Aldrich), THF (Roth, 99%), *n*-hexane (Roth, 98%) were dried over sodium and distilled prior to use. $MgSO_4$ (Sigma-Aldrich, 99.5%), NaOH (Honeywell, 99%), *n*-BuLi (Sigma-Aldrich, 2.5 M in hexane), 1-bromo-3,5-di-tert-butylbenzene (98% ArkPharm, procured via ChemPur). $CDCl_3$ (Roth, 99.8%), $CuOTf_2$ (ABCR), 98%) was used as received. C_6D_6 (Roth, 99.5%) was dried over sodium and distilled prior to use.

The protonated ligand **RH**, its corresponding potassium salt **RK** and [$(DippNSiMe_3)_2Cu$] were prepared according to literature protocols.[1,2]

IR spectra were recorded on a Bruker Alpha spectrometer using the attenuated total reflection (ATR) technique on powdered samples.

Elemental analyses were obtained with a Vario Micro Cube (Elementar Analysensysteme GmbH) in the institutional technical laboratories of the Karlsruhe Institute of Technology (KIT).

NMR spectra were acquired on a Bruker Advance 400 MHz spectrometer. Reported chemical shifts are referenced to the ^{1}H and ^{13}C NMR resonances of the deuterated solvent.[3] Coupling constants J are given in Hertz as positive values regardless of their real individual sign. ^{1}H, ^{31}P NMR spectra were obtained at 400.1, 121.5 MHz, respectively.

EPR spectra were recorded on a Bruker EMXplus X-band spectrometer (microwave frequency: 9.43 GHz). The spectra were simulated using the MATLAB/easyspin package.[4]

The UV/Vis spectra were recorded using a Mettler-Toledo Spektralphotometer UV7 in quartz cuvettes (d = 1 cm) in solution. To subtract the solvent, the sample was measured relative to the pure solvent.

Mass spectra were recorded on an Advion expressionL CMS mass spectrometer with an ASAP probe (Atmospheric Solids Analysis Probe) using APCI (atmospheric pressure chemical ionization).

Cyclic voltammetry measurements were performed at room temperature under argon with an EG&G potentiostat (PAR-model 263A) and an electrochemical cell for sensitive compounds. We used a freshly polished Pt disk working electrode, a Pt wire as counter electrode, as well as an Ag (pseudo)reference electrode and $[nBu_4N][Al(OC(CF_3)_3)_4]$ as electrolyte. Potentials were calibrated against the Fc/Fc^{+} couple (internal standard).

Single crystals were mounted in perfluoropolyalkyl ether oil on a cryo loop and then brought into the cold nitrogen stream of a low-temperature device (Oxford Cryosystems Cryostream unit) so that the oil solidified. Diffraction data were collected using a Stoe IPDS II diffractometer and graphite-monochromated Mo-K_{α} (0.71073 Å) or a Stoe STADIVARI diffractometer and Ga-K_{α} (1.34143 Å) radiation. The structures were solved by direct methods with SHELXS[5] or by intrinsic phasing with SHELXT[6] followed by full-matrix least-squares refinement using SHELXL-2014/7[7] and the ShelXle GUI.[8] All non-hydrogen atoms were refined anisotropically. The contribution of the hydrogen atoms, in their calculated positions, was included in the refinement using a riding model.

XANES: Samples were prepared in a glove box under Ar atmosphere as powder film on a scotch tape and covered with another scotch tape to prevent oxidation during the maximum 10 minutes lasting transfer into the vacuum chamber for X-ray absorption spectroscopy. Cu K-edge X-ray absorption spectra were measured at the SUL-X beamline of the synchrotron radiation facility of the KIT using a wiggler as radiation source and a fixed exit double crystal monochromator with Si(11) crystals for tuning the energy. Energy step size across the XANES region was 0.3 eV. Energy was calibrated by assigning the first inflection point of a Cu metal foil spectrum to 8979 eV. Measurements were performed under vacuum to avoid oxidation. Sample spectra were recorded with a collimated beam at sample position of about 0.8 x 0.8 mm^2 in transmission and fluorescence mode (Cu K_{α}) using ionization chambers and a seven element SDD detector (Rayspec) at Falcon electronics. Fluorescence spectra were used for further data processing by applying pre- and post-edge background corrections using a linear and a polynomial function, respectively. Background corrected data were finally normalized to an edge jump of one to enable comparison between spectra. For data processing the ATHENA program of the IFEFFIT package was used (Ravel and Newville, 2005).[9]

2 Syntheses

2.1 RCuOTf (1)

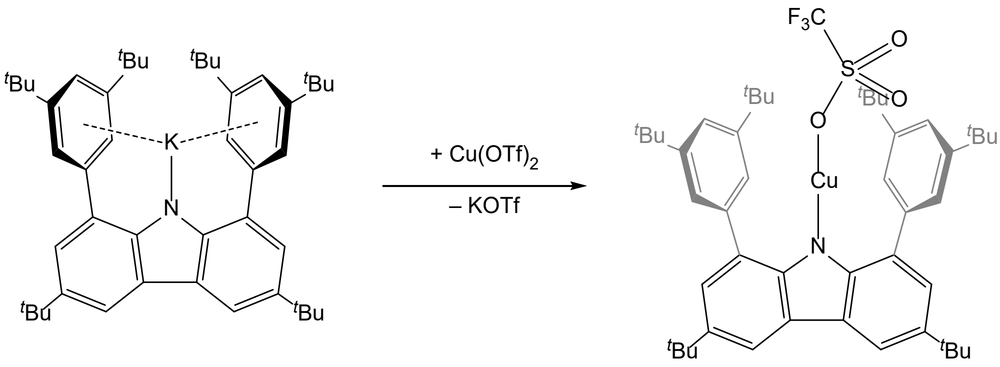

RH (200 mg, 0.305 mmol) and Benzyl potassium (39.6 mg, 0.305 mmol) were added in a Schlenk tube and solved in THF. After a few minutes **RK** as a yellow fluorescent solution has formed. To this solution $Cu(OTf)_2$ (110 mg, 0.305 mmol) was added. This causes an immediate colour change from fluorescent yellow to deep violet. The solution was stirred for two hours and dried under vacuo. The residue was solved in Et_2O, filtered via a syringe filter and dried again under vacuo, affording the product as a dark violet crystalline solid (205 mg, 0.236 mmol, 77 %). Recrystallisation from hexane afforded single crystals for X-ray diffraction.

EA calc. (found): C 67.83 (70.33), H 7.44 (6.99), N 1.61 (1.57), S 3.70 (3.16).

MS (APCI-) m/z: [M] calc. 866.39, found. 866.8; (APCI+) m/z: [M-OTf] calc. 717.43, found 717.8, [RH] calc. 655.51, found 655.8.

UV/Vis (Hexane, λ_{max}): 787 nm (ε = 1.097 · 10^3 l mol^{-1} cm^{-1}), 550 nm (ε = 0.670 · 10^3 l mol^{-1} cm^{-1}).

IR (ATR, cm^{-1}) $\tilde{\upsilon}$: 2955 (s), 2868 (m), 1589 (m), 1533 (m), 1476 (m), 1393 (m), 1362 (s), 1331 (s), 1286 (m), 1261 (m), 1245 (s), 1233 (s), 1196 (vs), 1168 (s), 1056 (s), 1010 (s), 925 (m), 900 (m), 870 (vs), 752 (m), 715 (s), 692 (m), 664 (m), 633 (vs), 592 (m), 578 (m), 565 (m), 529 (m), 513 (m), 481 (m), 456 (m), 432 (m), 416 (s), 409 (s), 400 (s), 392 (s).

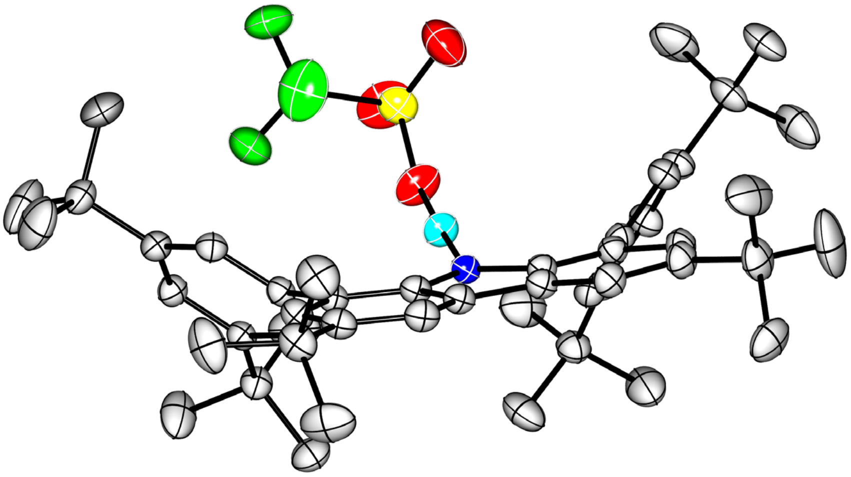

Figure S1: Molecular structure of **1**.

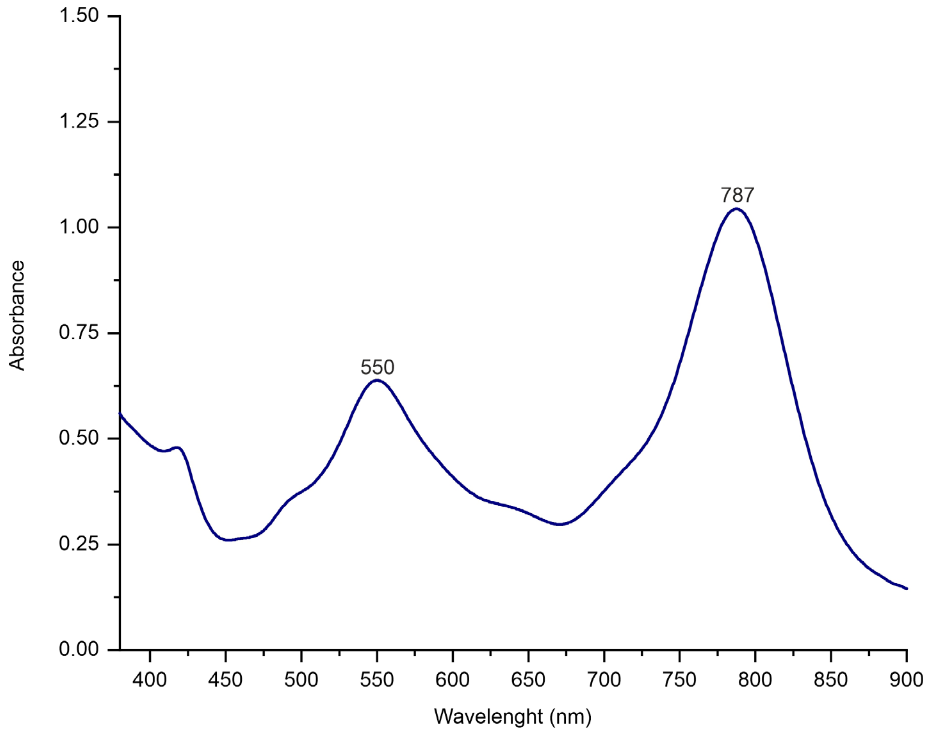

Figure S2: UV/Vis spectrum of **1**.

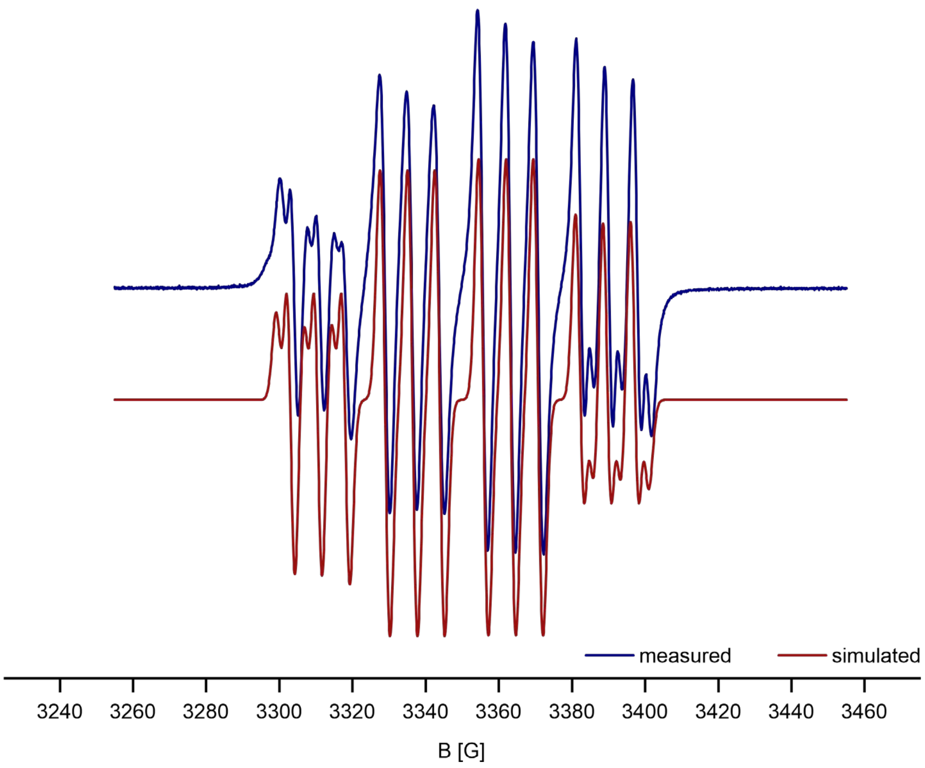

Figure S3: EPR spectrum of **1** at 295 K in hexane.

Table S1: Parameters for the simulation of the EPR spectrum at 295 K of **1**.

Parameter	Value
frequency [GHz]	9.428
g	2.01063
A_{iso}(N) [MHz]	21.03
A_{iso}(Cu) [MHz]	74.23
line width [mT]	0.3000

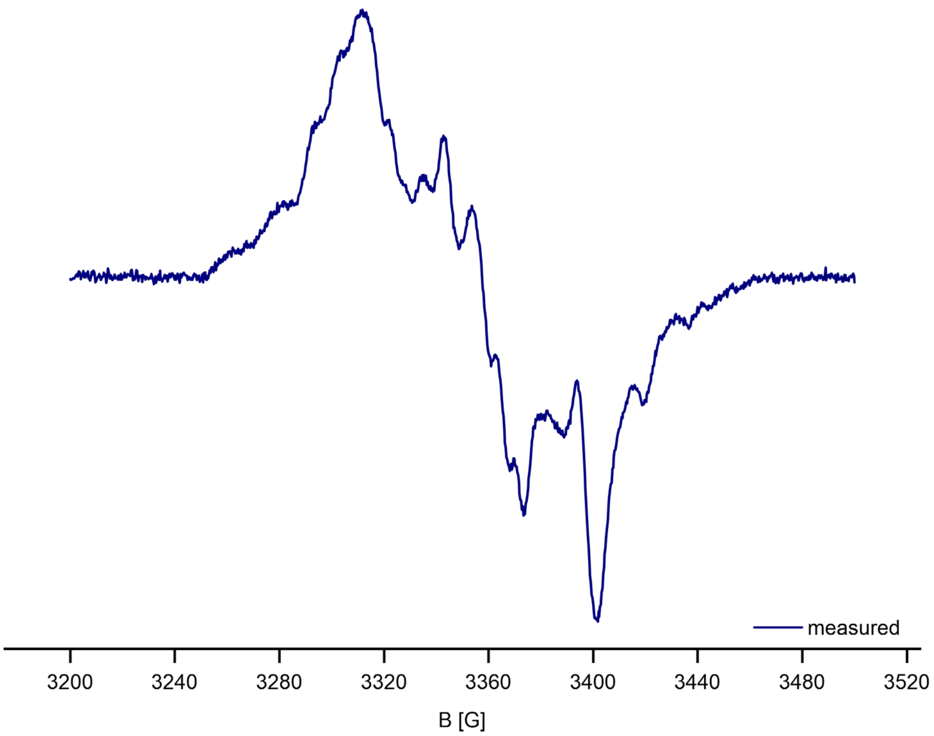

Figure S4: EPR spectrum of **1** at liquid nitrogen cooling (~77 K) in hexane (Frequency: 9.432 GHz).

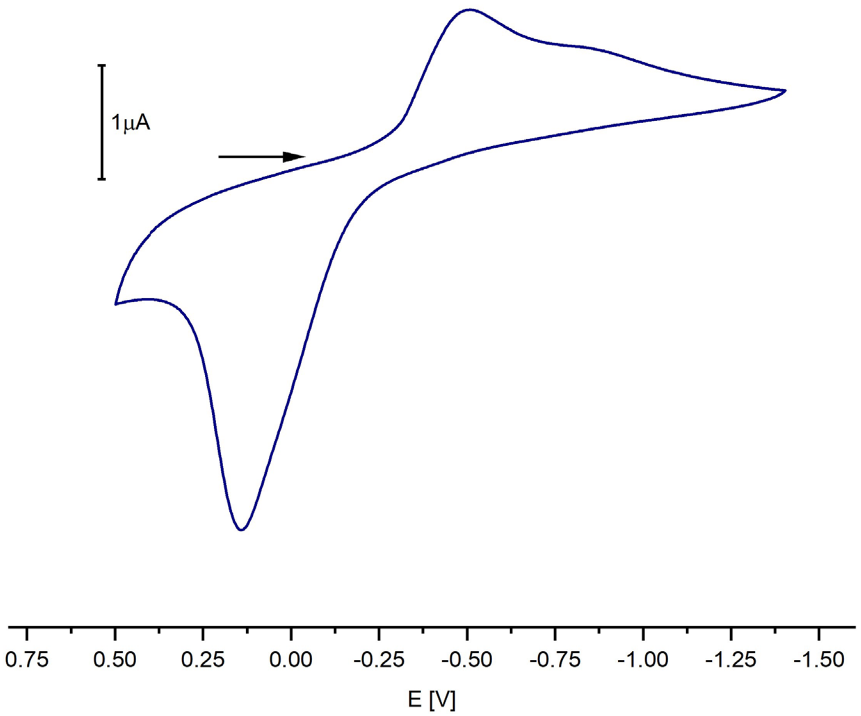

Figure S5: CV of **1** in THF with $[nBu_4N][Al(OC(CF_3)_3)_4]$ vs Fc/Fc^+. Scan rate 250 mVs^{-1}.

2.2 RCuOSO$_3$Et (2)

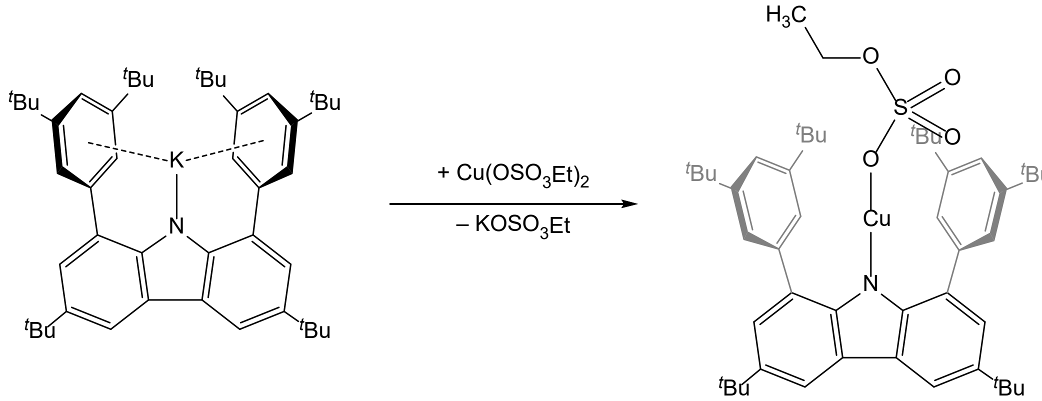

RK (150 mg, 0.214 mmol) and Cu(OSO$_3$Et)$_2$·2 THF (97.7 mg, 0.214 mmol) were added in a Schlenk tube and solved in THF. This causes an immediate colour change from fluorescent yellow to deep violet. The solution was stirred for two hours and dried under vacuo. The residue was solved in Et$_2$O, filtered via a syringe filter and dried again under vacuo, affording the product as a dark violet crystalline solid (110 mg, 0.130 mmol, 61 %).

UV/Vis (Hexane, λ_{max}): 783 nm, 578 nm.

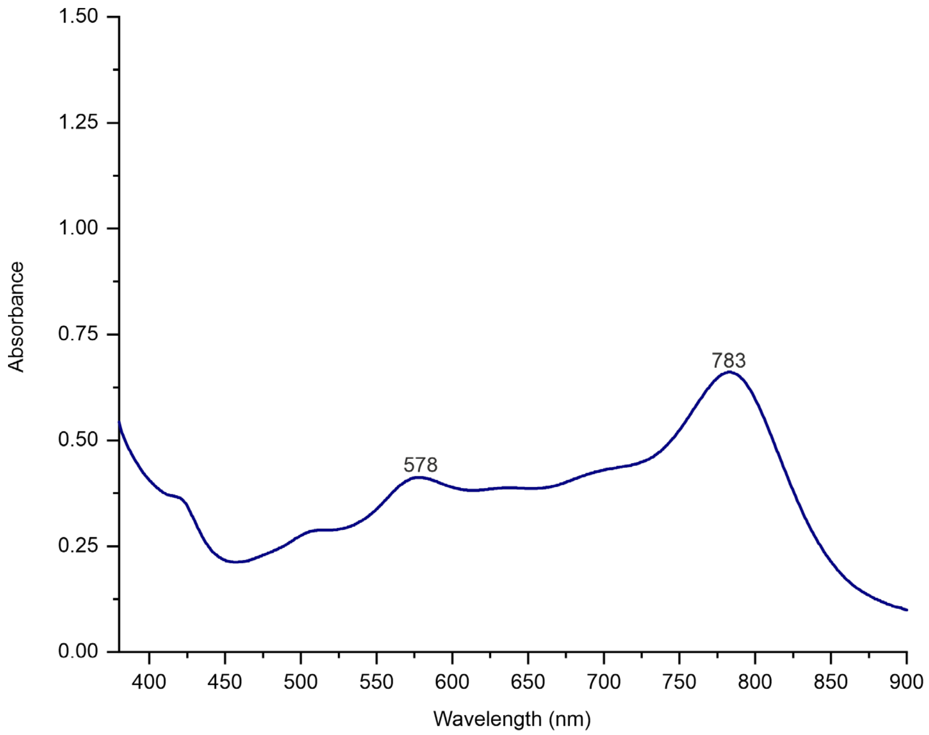

Figure S6: UV/Vis spectrum of **2** (sample decomposed rapidly in sample container).

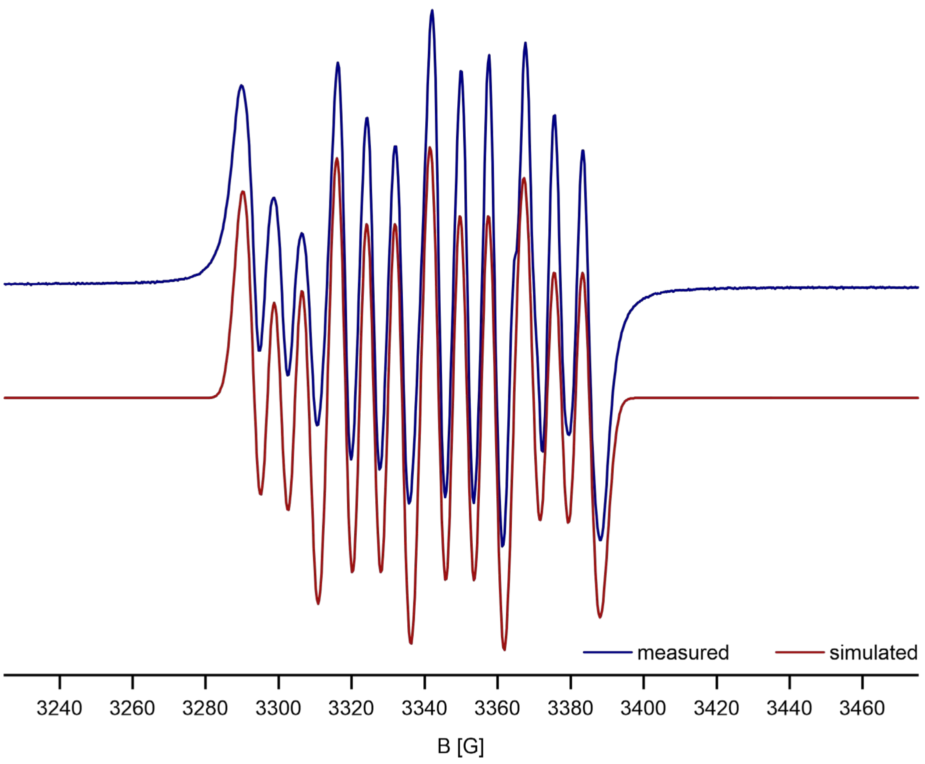

Figure S7: EPR spectrum of **2** at 295 K in hexane.

Table S2: Parameters for the simulation of the EPR spectrum at 295 K of **2**.

Parameter	Value
frequency [GHz]	9.429
g	2.01750
A_{iso}(N) [MHz]	21.99
A_{iso}(Cu) [MHz]	70.96
line width [mT]	0.5845

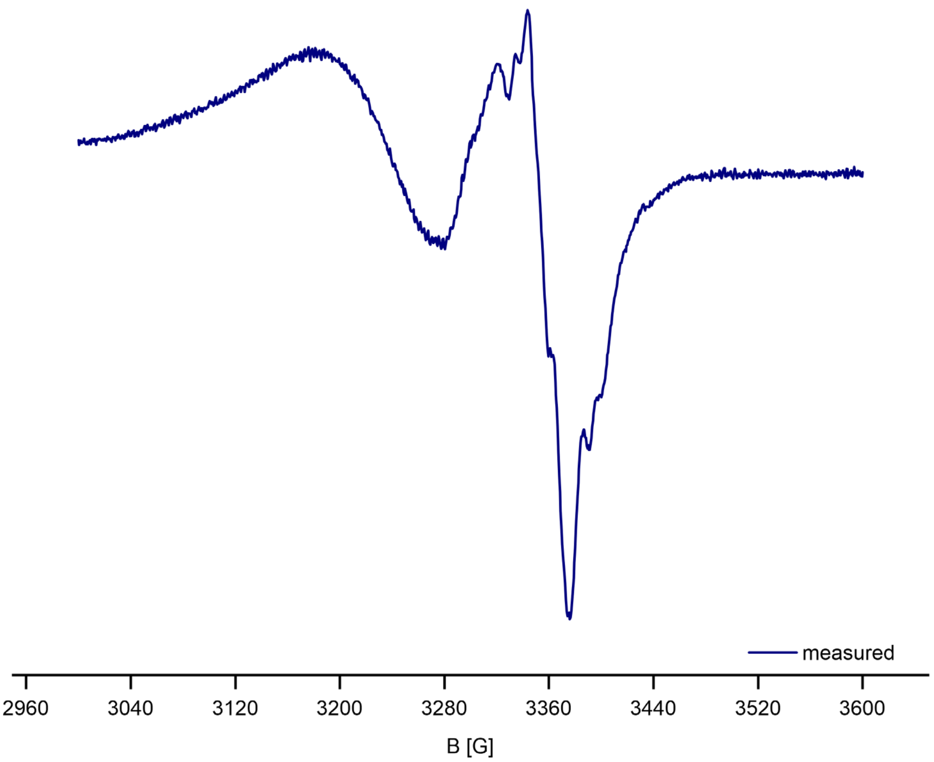

Figure S8: EPR spectrum of **2** at liquid nitrogen cooling (~77 K) in hexane (Frequency: 9.434 GHz).

2.3 [RCu(THF)][Cu(NTf$_2$)$_2$] (3)

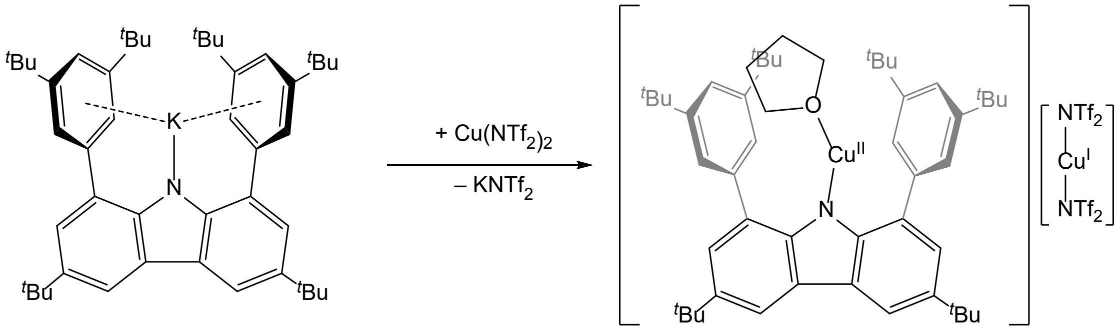

RK (100 mg, 0.142 mmol) and Cu(NTf$_2$)$_2$·2 AgCl (130 mg, 0.142 mmol) were added in a Schlenk tube and solved in THF This causes an immediate colour change from fluorescent yellow to deep violet. The solution was stirred for 15 minutes and dried under vacuo.

The residue was extracted with hexane and dried under vacuo, affording the product as a dark violet crystalline solid (41.1 mg, 0.032 mmol, 23 %). Recrystallisation from hexane afforded single crystals for X-ray diffraction.

EA calc. (found): C 44.34 (44.88), H 4.19 (4.29), N 3.23 (3.06), S 9.86 (10.00).

UV/Vis (Hexane, λ_{max}): 756 nm (ε = 3.363 · 10^3 l mol^{-1} cm^{-1}), 513 nm (ε = 2.070 · 10^3 l mol^{-1} cm^{-1}).

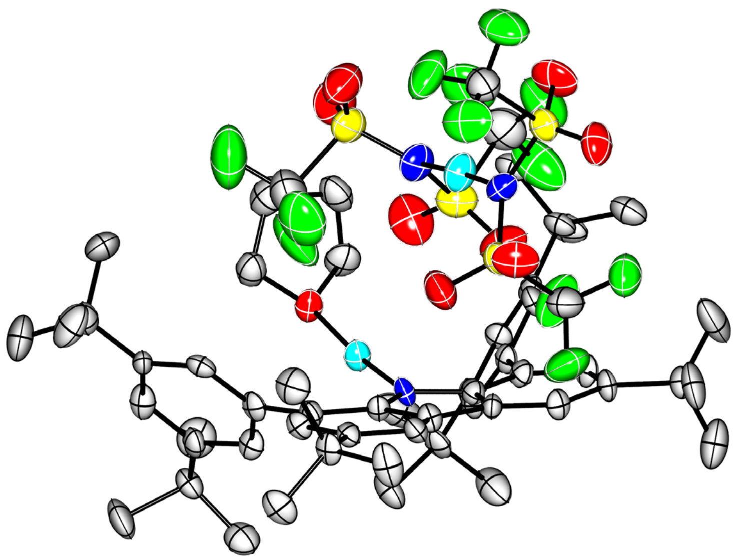

Figure S9: Molecular structure of **3**.

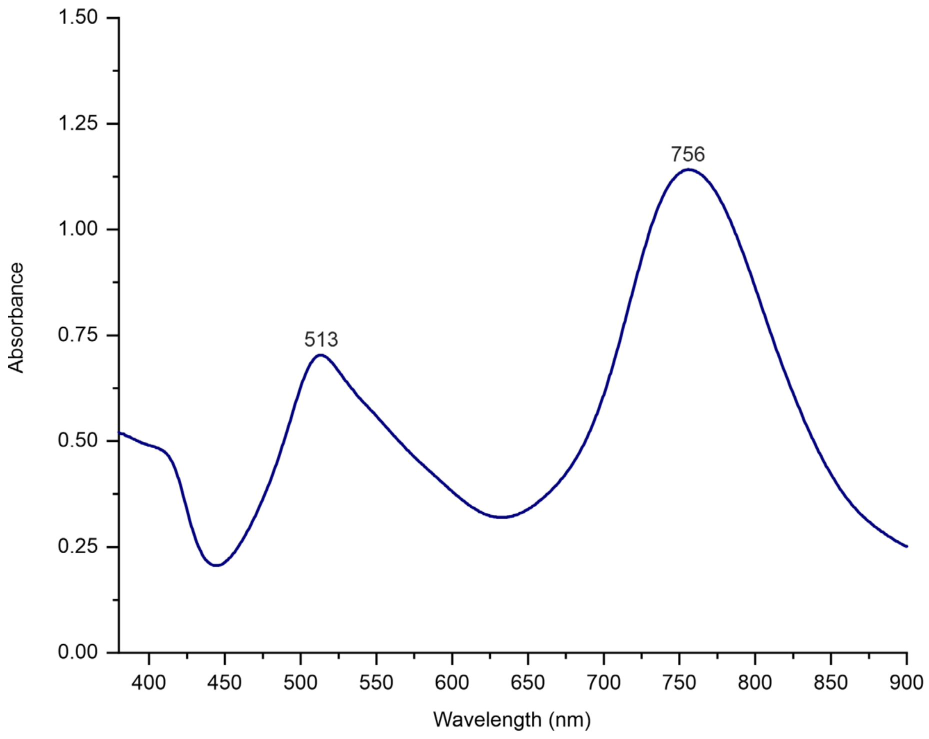

Figure S10: UV/Vis spectrum of **3**.

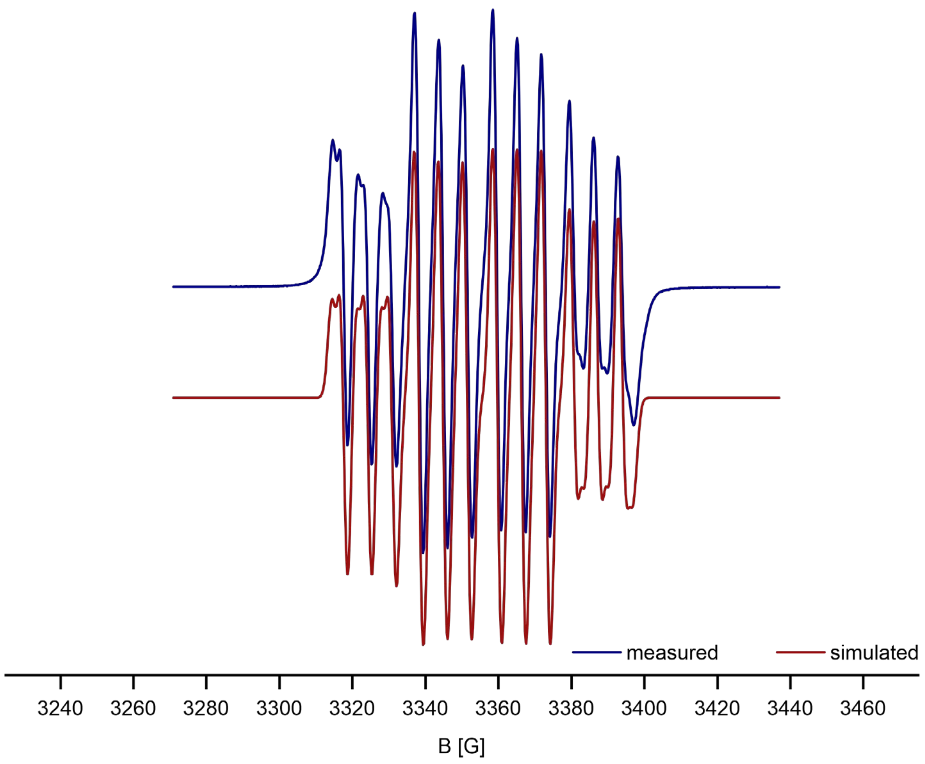

Figure S11: EPR spectrum of **3** at 295 K in hexane.

Table S3: Parameters for the simulation of the EPR spectrum at 295 K of **3**.

Parameter	Value
frequency [GHz]	9.434
g	2.01076
A_{iso}(N) [MHz]	22.45
A_{iso}(Cu) [MHz]	71.27
line width [mT]	0.3428

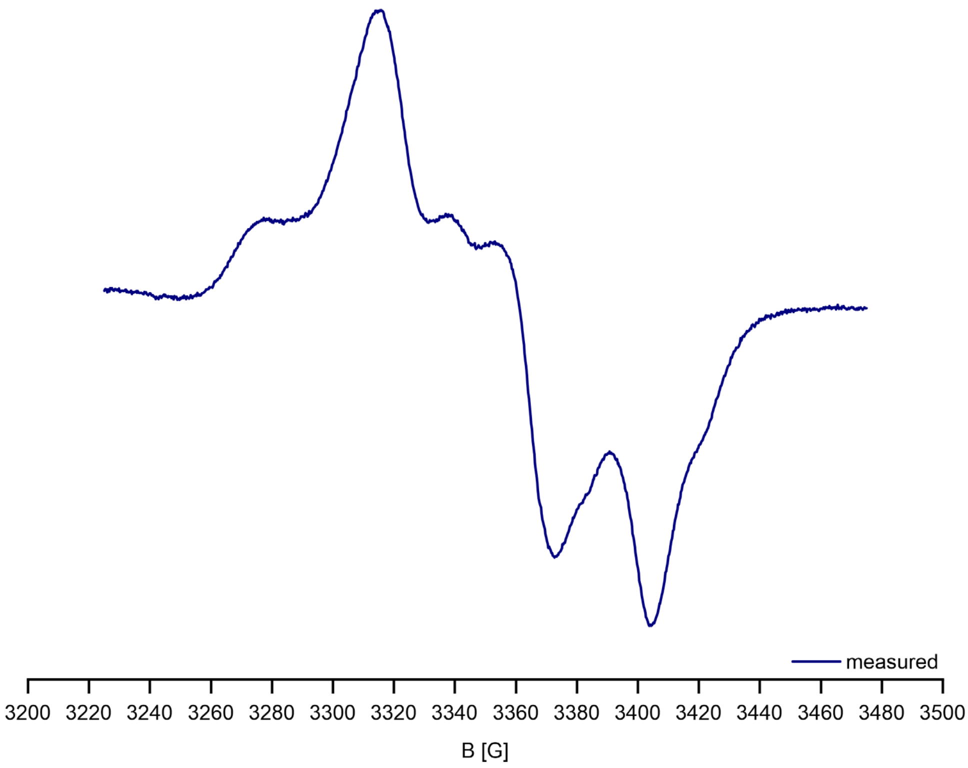

Figure S12: EPR spectrum of **3** at liquid nitrogen cooling (~77 K) in hexane (Frequency: 9.434 GHz).

2.4 $RCuNTf_2$ (4)

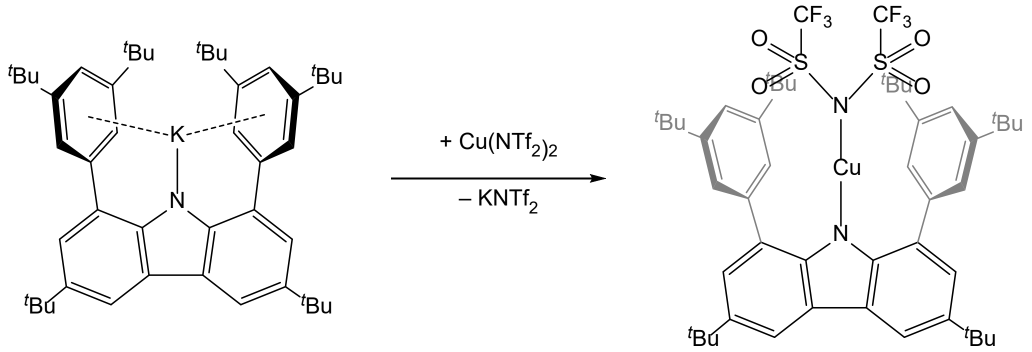

RK (100 mg, 0.142 mmol) and $Cu(NTf_2)_2{\cdot}2\,AgCl$ (130 mg, 0.142 mmol) were added in a Schlenk tube and solved in hexane. The solution was sonicated for two hours and a deep violet suspension has formed. The suspension was filtered via a syringe filter and dried under vacuo affording the product as a dark violet crystalline solid(91.1 mg, 0.091 mmol, 64 %). Recrystallisation from hexane afforded single crystals for X-ray diffraction.

EA calc. (found): satisfactory analysis could not be obtained.

UV/Vis (Hexane, λ_{max}): 772 nm (ε = 1.997 · 10^3 l mol^{-1} cm^{-1}), 510 nm (ε = 1.401 · 10^3 l mol^{-1} cm^{-1}).

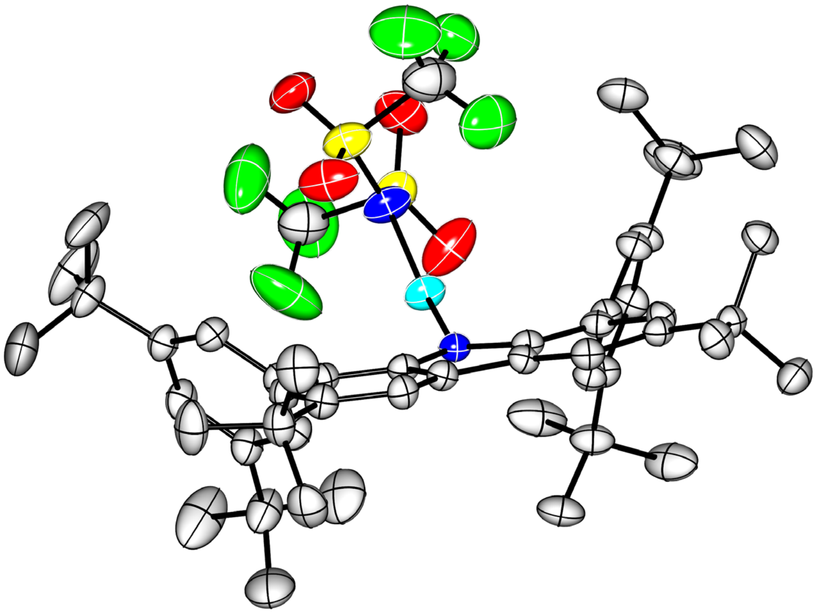

Figure S13: Molecular structure of **4.**

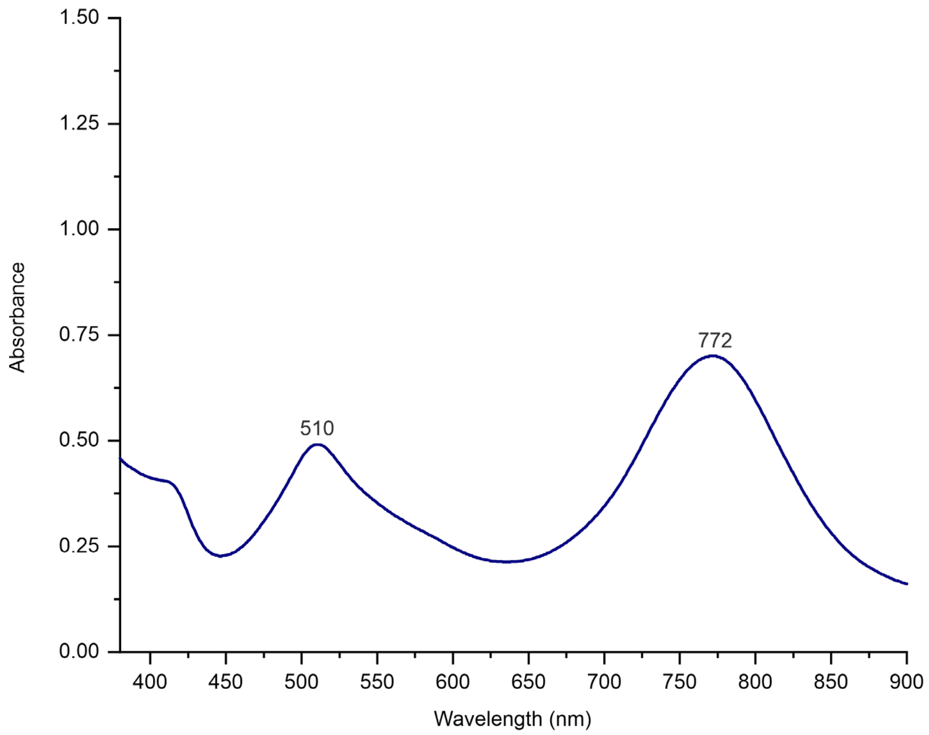

Figure S14: UV/Vis spectrum of **4**.

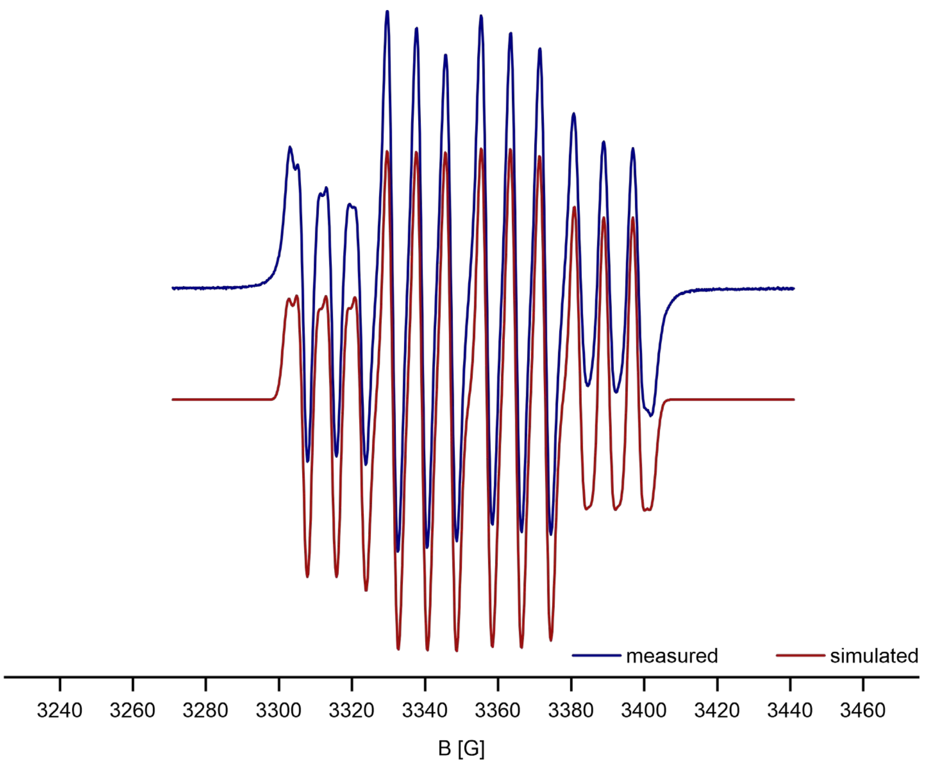

Figure S15: EPR spectrum of **4** at 295 K in hexane.

Table S4: Parameters for the simulation of the EPR spectrum at 295 K of **4**.

Parameter	Value
frequency [GHz]	9.434
g	2.01408
A_{iso}(N) [MHz]	22.51
A_{iso}(Cu) [MHz]	71.20
line width [mT]	0.3483

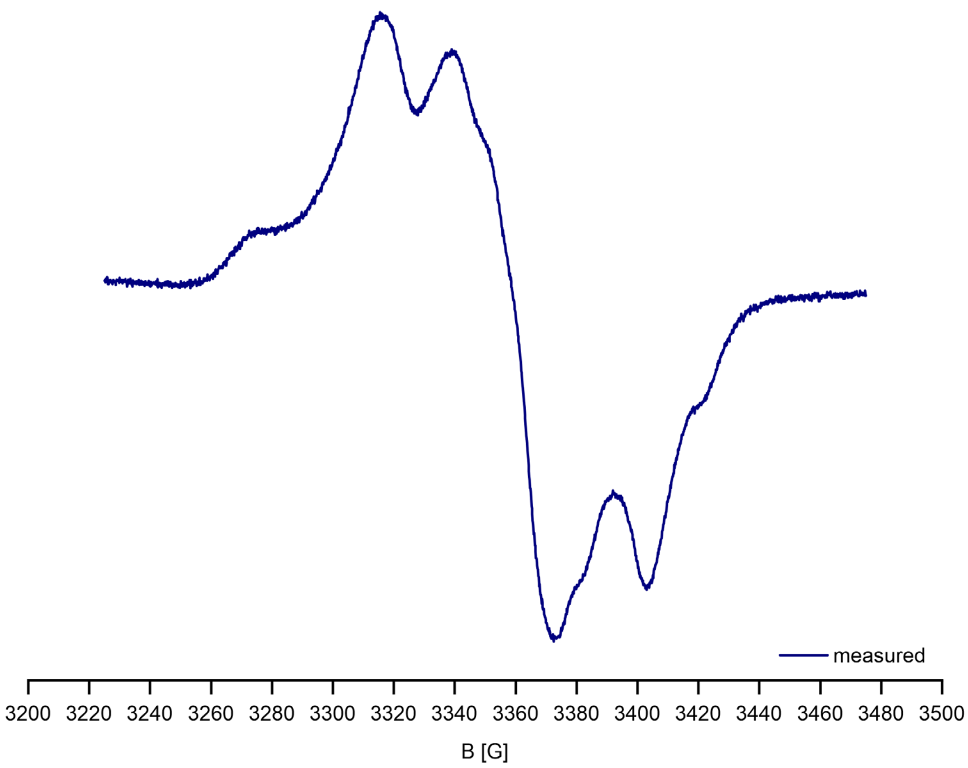

Figure S16: EPR spectrum of **4** at liquid nitrogen cooling (~77 K) in hexane (Frequency: 9.433 GHz).

2.5 RCu-PPh3 (5)

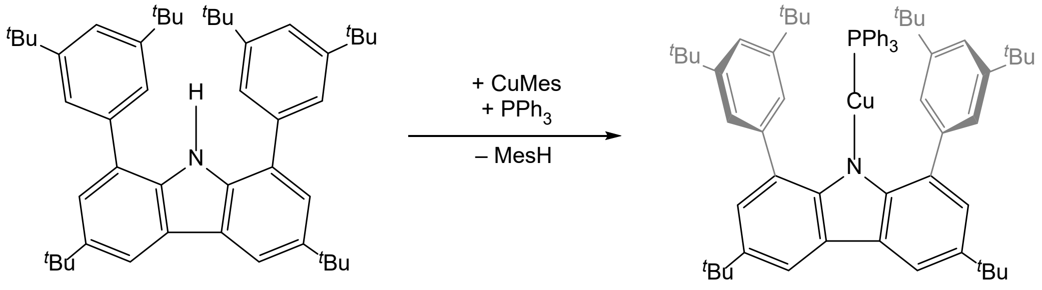

RH (400 mg, 0.609 mmol), CuMes (112 mg/ 0.612 mmol) and PPh_3 (160 mg/0.609 mmol) were added in a Schlenk tube and solved in THF. The solution was heated to 80°C for 5 hours and a golden yellow solution has formed. The solution was dried under vacuo, extracted with hexane, filtered via a syringe filter and dried again under vacuo affording the product as a yellow solid (340 mg, 0.347 mg, 57%. Recrystallisation from Et_2O afforded single crystals for X-ray diffraction.

^{1}H-NMR (300.1 MHz, C_6D_6): δ (ppm) = 1.26 (s, 36 H, Ar-^{t}Bu), 1.56 (s, 18 H, Carb-^{t}Bu), 6.66 (m, 6H, PPh_3), 6.88 (m, 9 H, PPh_3), 7.51 (t, J_{HH} = 1.8 Hz, 2 H, p-C*H*), 7.77(d, J_{HH} = 2.0 Hz, 2 H, $C^{2,7}$*H*), 7.85 (d, J_{HH} = 1.8 Hz ,4 H, o-C*H*), 8.63 (d, J_{HH} = 2.0 Hz, 2 H, $C^{4,5}$*H*).

^{31}P-NMR (121.5 MHz, C_6D_6): δ (ppm) = 8.37 (s, PPh_3).

MS (APCI-) m/z: [M-(Ph)$_3$] calc. 748.41, found. 749.8/ (APCI+) m/z: [M+H] calc. 980.53, found. 980.9.

EA calc. (found.): C 79.69 (80.04) H 8.50 (8.53) N 1.33 (1.33).

IR (ATR, cm^{-1}) ῦ: 3717 (vw), 3513 (vw), 3057 (vw), 2952 (m), 2903 (vw), 2864 (vw), 1586 (w), 1477 (w), 1435 (w), 1388 (vw), 1360 (w), 1286 (vw), 1267 (vw), 1246 (vw), 1226 (m), 1200 (vw), 1181 (vw), 1097 (w), 1027 (vw), 995 (vw), 923 (vw), 903 (vw), 861 (m), 846 (w), 744 (m), 716 (m), 707 (w), 692 (vs), 643 (w), 616 (vw), 591 (vw), 565 (vw), 554 (vw), 533 (m), 517 (m), 501 (s), 486 (w), 459 (m), 451 (w), 434 (s), 408 (vs), 398 (m), 386 (m).

2.6 Cu[N($SiMe_3$)Dipp]$_2$ (B)

Cu[N($SiMe_3$)Dipp]$_2$ (B) was prepared according to literature procedures and determined by measuring the crystal cell by X-ray diffraction.

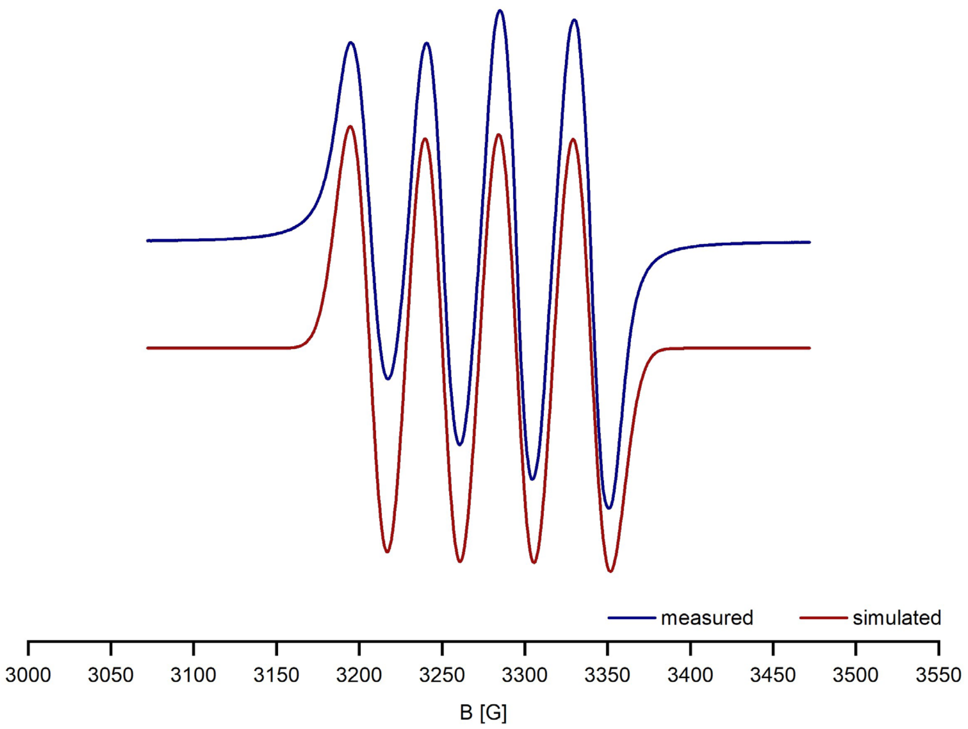

Figure S17: EPR spectrum of **B** at 295 K in hexane.

Table S5: Parameters for the simulation of the EPR spectrum at 295 K of **B**.

Parameter	Value
frequency [GHz]	9.4348
g	2.06029
A_{iso}(Cu) [MHz]	125.82
line width [mT]	2.7140

2.7 XANES

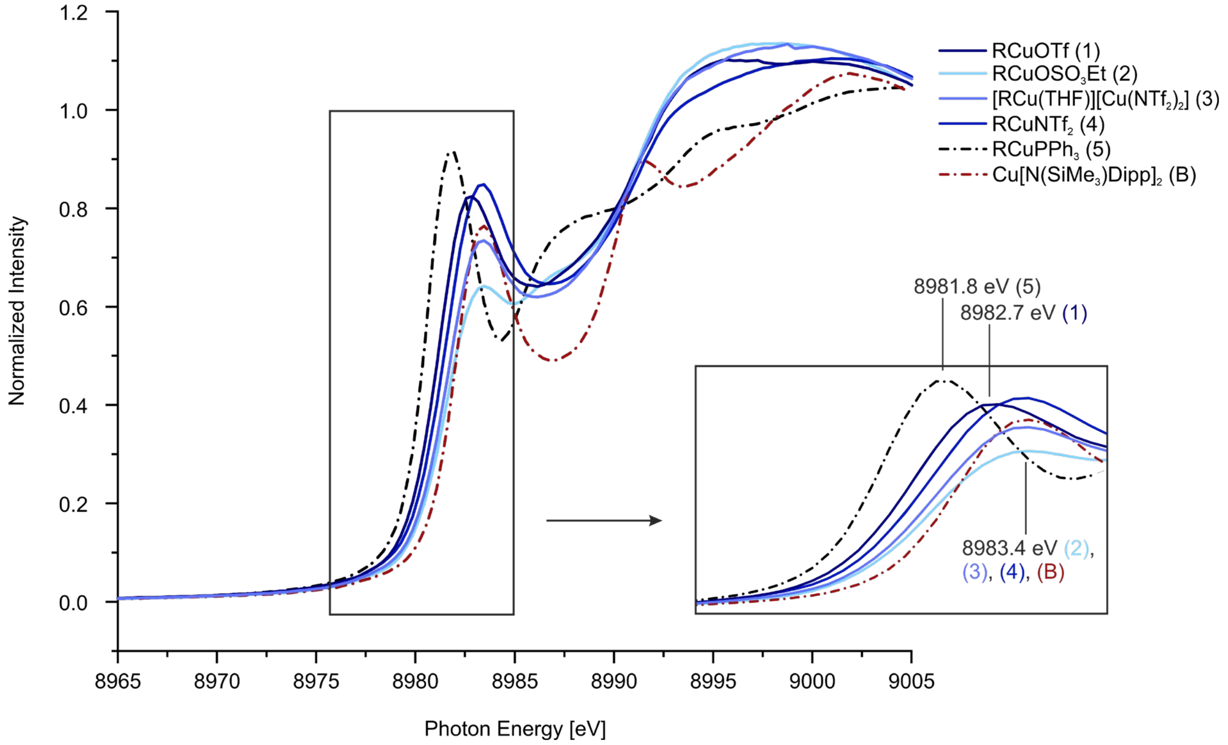

Figure S18: Cu-K-edge XAS spectra of **1**-**5** and **B**.

2.8 Radical Intermediates

2.8.1 RK + Cu(O^{t}Bu)$_2$

RK (80 mg, 0.114 mmol) and Cu(O^{t}Bu)$_2$ (23,9 mg, 0.114 mmol) were combined in a Schlenk tube and dissolved in hexane. The solution was sonicated for two hours and a yellow turbid solution formed. EPR spectroscopy of this solution showed a radical intermediate.

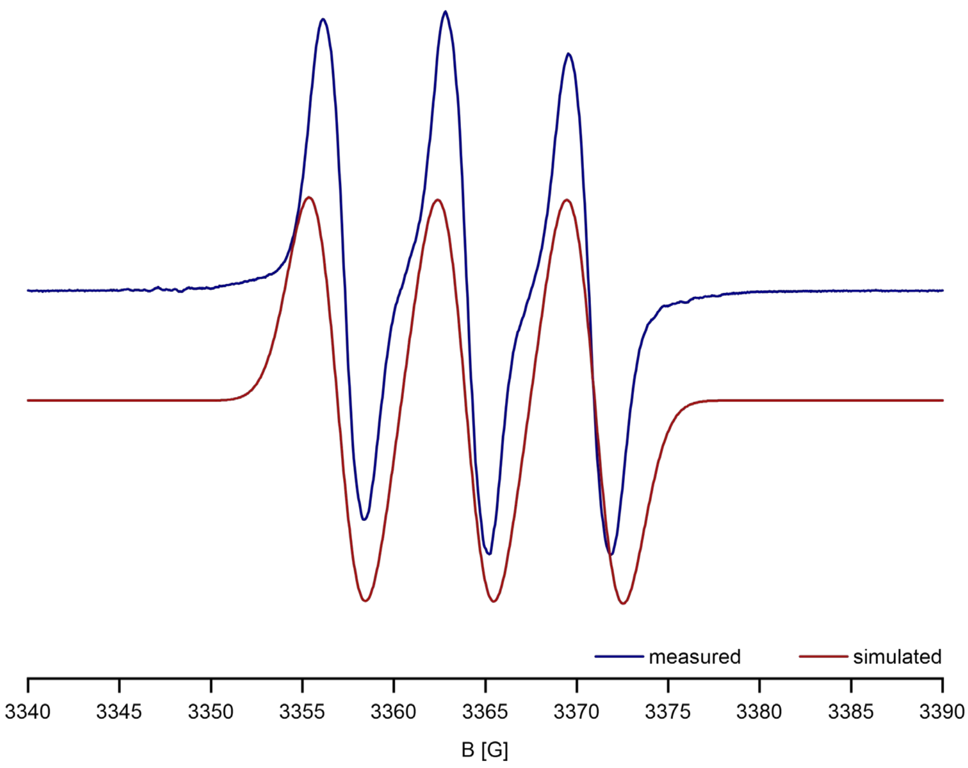

Figure S19: EPR spectrum at 295 K in hexane.

Table S6: Parameters for the simulation of the EPR spectrum at295 K.

Parameter	Value
frequency [GHz]	9.428
g	2.01196
A_{iso}(N) [MHz]	39.73
line width [mT]	0.7331

2.8.2 RK + $Cu(OTos)_2$

RH (100 mg, 0.152 mmol) and benzyl potassium (19.8 mg, 0.152 mmol) were combined in a Schlenk tube and dissolved in toluene. After a few minutes **RK** as a yellow fluorescent solution had formed. To this solution $Cu(OTos)_2$ (65 mg, 0.160 mmol) was added. This caused an immediate colour change from fluorescent yellow to a red solution. EPR spectroscopy of this solution showed a radical intermediate.

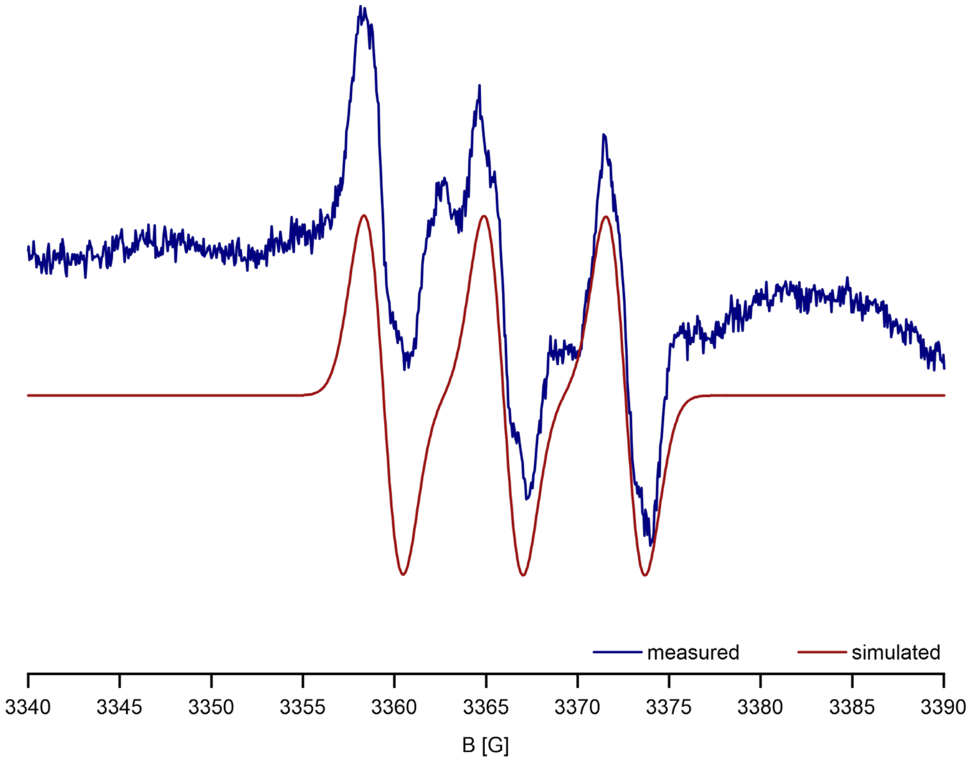

Figure S20: EPR spectrum at 295 K in toluene.

Table S7: Parameters for the simulation of the EPR spectrum at295 K.

Parameter	Value
frequency [GHz]	9.434
g	1.98044
A_{iso}(N) [MHz]	61.07
line width [mT]	0.8378

2.8.3 RK + $Cu(OAc^F)_2$

RH (200 mg, 0.305 mmol) and benzyl potassium (39.6 mg, 0.305 mmol) were combined in a Schlenk tube and dissolved in THF. After a few minutes **RK** as a yellow fluorescent solution had formed. To this solution a solution of "$Cu(CuOAc^F)_2$" = $[Cu_5(O_2C_2F_3)_8(OH)_2]$ (62.9 mg, 0.051 mmol) in THF was added. After a few minutes a brownish turbid solution has formed. The solution was stirred for one hour and dried in vacuo. The residue was extracted with hexane and filtered via a syringe filter. EPR spectroscopy of the filtrate showed a radical intermediate.

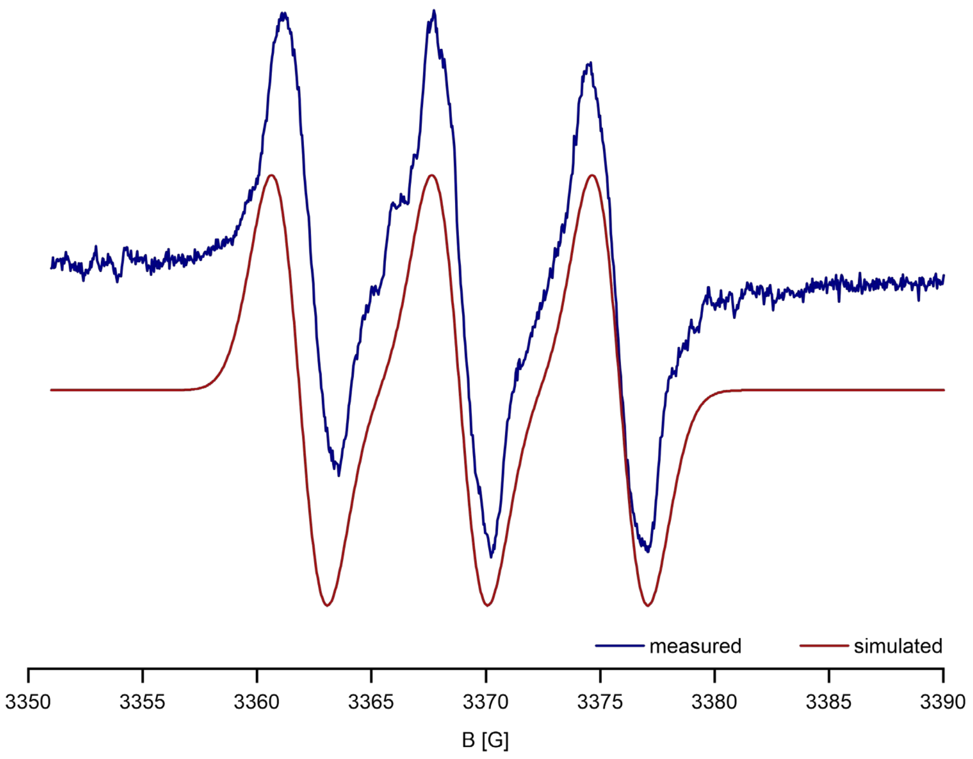

Figure S21: EPR spectrum at 295 K in hexane.

Table S8: Parameters for the simulation of the EPR spectrum at295 K.

Parameter	Value
frequency [GHz]	9.441
g	2.00155
A_{iso}(N) [MHz]	44.29
line width [mT]	0.6460

3 Crystallography

Table S9: Crystallographic details for **1**, **3**, **4** and **5**.

	**RCuOTf (1)**	**[RCuTHF][Cu(NTf$_2$)$_2$] (3)**	**RCuNTf$_2$ (4)**	**RCuPPh$_3$·Et$_2$O (5)**
CCDC #	2004478	2004479	2004480	2063690
Empirical formula	$C_{49}H_{64}CuF_3NO_3S$	$C_{56}H_{72}Cu_2F_{12}N_3O_9S_4$	$C_{50}H_{64}CuF_6N_2O_4S_2$	$C_{70}H_{89}CuNOP$
FW [g mol^{-1}]	867.61	1414.48	998.69	1054.93
Wavelength [Å]	0.71073	1.34143	0.71073	0.71073
Temperature [K]	200(2)	150(2)	200(2)	200(2)
Crystal system	triclinic	monoclinic	monoclinic	monoclinic
Space group	*P-1*	$P2_1$	$P2_1/n$	*$P2_1/c$*
a [Å]	12.613(3)	10.0221(5)	16.253	10.8262(3)
b [Å]	13.408(3)	22.3696(9)	16.569	23.3749(4)
c [Å]	14.565(3)	14.5434(8)	19.251	24.4417(6)
α [°]	88.91(3)	90	90	90
β [°]	79.72(3)	96.037(4)	92.37	90.418(2)
γ [°]	79.12(3)	90	90	90
V [Å^3]	2379.9(9)	3242.4(3)	5179.6	6185.1(3)
Z	2	2	4	4
ρ_{calc} (g·cm^{-3})	1.211	1.449	1.281	1.133
μ	0.554	4.822	0.567	0.421
F(000)	922.0	1462.0	2100.0	2272.0
reflections collected	31020	19567	25477	45722
independent reflections	12788	9775	10182	14177
reflectionsGT (I > 2σ(I))	11475	4884	6993	11461
R_{int}	0.0256	0.0981	0.0323	0.0269
parameters	609	930	638	750
restraints	330	400	168	324
GooF	1.017	0.898	1.029	1.076
R1	0.0392	0.0636	0.0498	0.0543
R1 (all)	0.0438	0.1539	0.0813	0.0730
wR2	0.1106	0.1311	0.1241	0.1387
wR2 (all)	0.1147	0.1609	0.1427	0.1513
weight factors	0.0392<br>0.1147	0.0636<br>0.1609	0.0498<br>0.1427	0.0563<br>7.3792

4 Computational details

Geometry optimisations were performed using Gaussian16[10] utilizing the PBE1PBE level of theory, Def2SVP basis sets and empirical dispersion correction (GD3). EPR parameters were computed on the M062X level of theory with Def2TZVP basis sets. No solvent corrections were applied. All optimised molecular structures where checked to be minima on the energy hypersurface and possess no imaginary vibrational frequencies. Natural Bond Orbital Theory was applied to study the electronic states.[11]

4.1 Overview

Table S10: Summary of computational details.

	dispersion	no dispersion	ΔG	$\Delta\Delta G$	spin density	
compound	a.u.	a.u.	kJ/mol	kJ/mol	Cu	N
$[RCu]^+$	-3557.905773	-3557.805306	263.8	0.0	0.013	0.408
$[RCuTHF]^+$	-3789.848195	-3789.732095	304.8	41.0	0.051	0.396
$RCuSbF_6$	-4396.528566	-4396.414111	300.5	36.7	0.090	0.400
$RCuNTf_2$	-5382.951188	-5382.821483	340.5	76.8	0.109	0.392
$RCuBF_4$	-3981.842244	-3981.734716	282.3	18.5	0.145	0.396
RCuOTf	-4518.320150	-4518.204545	303.5	39.7	0.146	0.362
$RCuOSO_3Et$	-4335.368557	-4335.250339	310.4	46.6	0.165	0.355
$RCuN_3$	-3722.003243	-3721.898973	273.8	10.0	0.172	0.321
$RCuO^tBu^F$	-4682.420144	-4682.297482	322.0	58.3	0.178	0.349
RCuCl	-4018.057433	-4017.953891	271.8	8.1	0.183	0.363
RCuOTos	-4451.627706	-4451.502560	328.6	64.8	0.190	0.338
$RCuO_2C_2CF_3$	-4083.413949	-4083.303244	290.7	26.9	0.230	0.325
$RCuN(SiMe_3)_2$	-4430.252140	-4430.122021	341.6	77.9	0.259	0.304
$RCuO^tBu$	-3790.629758	-3790.510679	312.6	48.9	0.350	0.217
$Cu(N\{Si^iPr_3\}Dipp)_2$	-3970.219726	-3970.089540	341.8		0.228	0.356
$Cu(N\{SiMe_3\}Dipp)_2$	-3499.726033	-3499.641208	222.7		0.256	0.356
$Cu(N\{SiMe_3\}_2)_2$	-3384.384606	-3384.343414	108.1		0.305	0.318
$Cu(NTf_2)_2$	-5289.695971	-5289.666361	77.7		0.629	0.074

Table S11: Experimental and computational EPR parameters.

			g iso	A Cu iso	A N iso	CuX	Cuy	Cuz
RCuOTf	observed		**2.0106**	**74.23**	**21.03**			
	M062X	Def2TZVP	2.0189	-82.95	26.36	-49.26	-12.47	61.73
$RCuOSO_3Et$	observed		**2.0175**	**70.96**	**21.99**			
	M062X	Def2TZVP	2.0233	-85.81	26.47	-54.69	-16.41	71.10
$RCuNTf_2$	observed							
	M062X	Def2TZVP	2.0136	-82.01	26.96	-47.31	12.48	34.83
$[RCuTHF]^+$	observed		**2.0107**	**71.20**	**22.51**			
	M062X	Def2TZVP	2.0089	-89.92	26.14	-31.25	5.54	25.71
$RCuO^tBu$	observed							
	M062X	Def2TZVP	3.5654	561.30	6.02	-354.64	-197.31	551.95
$RCuO_2C_2F_3$	observed							
	M062X	Def2TZVP	2.9470	221.41	14.39	-381.47	-155.10	536.57
RCuOTos	observed		1.9799		50.18			
	M062X	Def2TZVP	2.0348	-94.89	26.44	-67.10	-28.95	96.04

4.2 [RCu(THF)][Cu(NTf_2)$_2$]

4.2.1 [RCu(THF)] fragment

	cation	neutral
	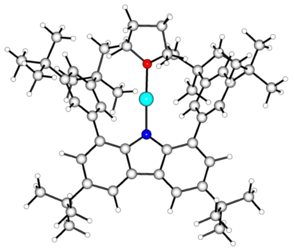	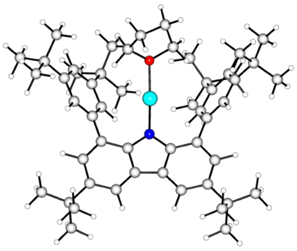
N–Cu	1.895	1.880
Cu–O	1.930	1.959
N–Cu–O	157.76	165.83
exp.		
N–Cu	1.845(6)	
Cu–O	1.873(5)	
N–Cu–O	170.3(3)	

4.2.2 [Cu(NTf_2)$_2$] fragment

	anion	neutral
	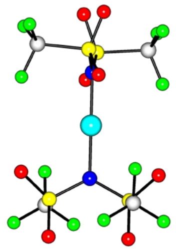	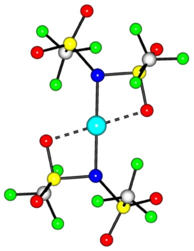
N–Cu	1.907, 1.906	1.953, 1.953
Cu–O	3.023, 3.025, 3.023, 3.026	2.041, 2.041, 3.656, 3.658
N–Cu–N	179.98	179.29
S-N-N-S	90.08	179.77
exp.		
N–Cu	1.890(7), 1.916(10)	
Cu–O	2.971(9), 2.964(14), 3.017(8), 3.021(11)	
N–Cu–N	177.0(5)	
S-N-N-S	104.0(9), 104.2(8), 106.7(11), 101.6(12)	

4.2.3 EPR parameters

	$[RCu(THF)]^+$	$[Cu(NTf_2)_2]$ (PBE1PBE, def2SVP)
g	2.0089	2.4275
$A_{iso}(^{63}Cu)$	89.92	15.34
$A_{iso}(^{14}N)$	26.14	46.78
exp.		
g	2.0107	
$A_{iso}(^{63}Cu)$	71.20	
$A_{iso}(^{14}N)$	22.51	

4.3 Spin density plots, isosurfaces at 0.005

4.3.1 RCuOTf

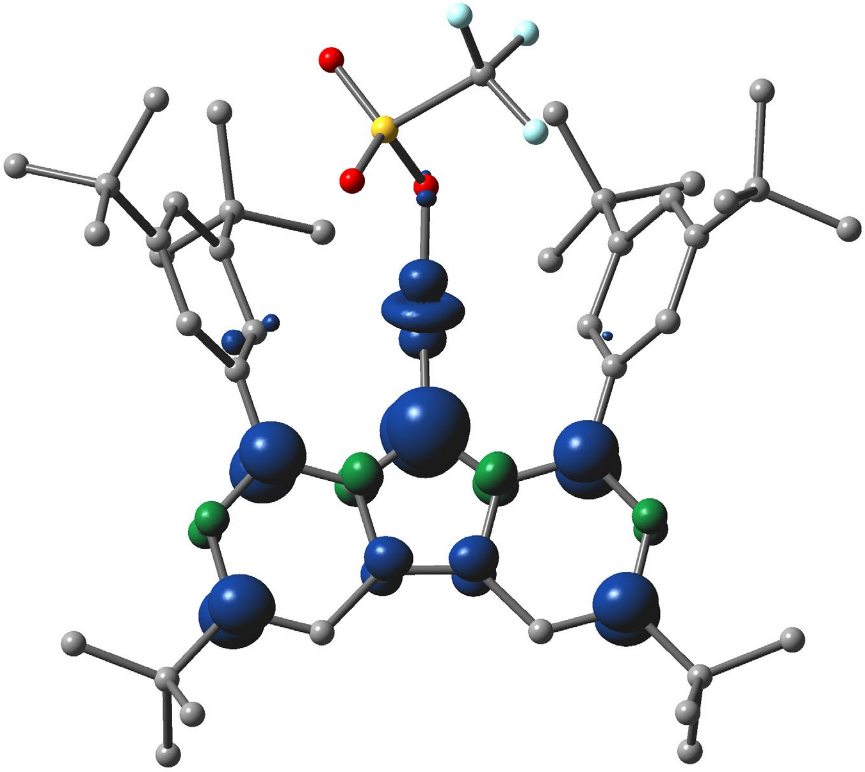

4.3.2 $RCuOSO_3Et$

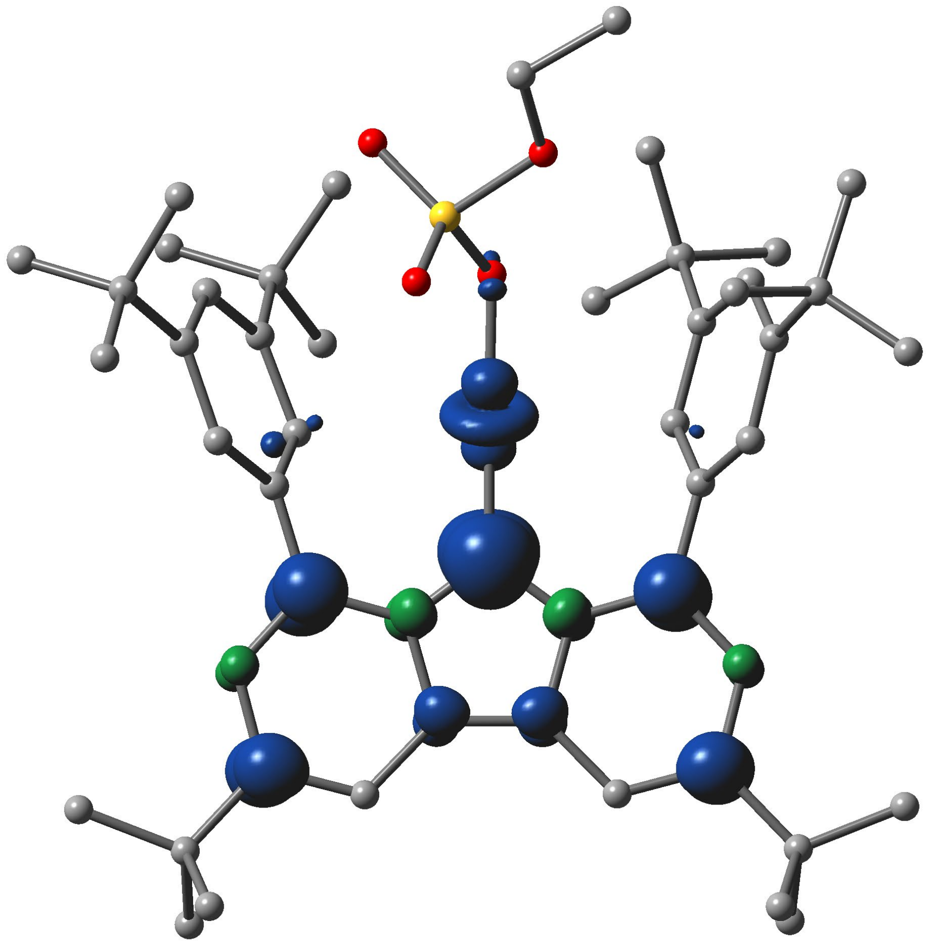

4.3.3 [RCu(THF)]$^{+}$

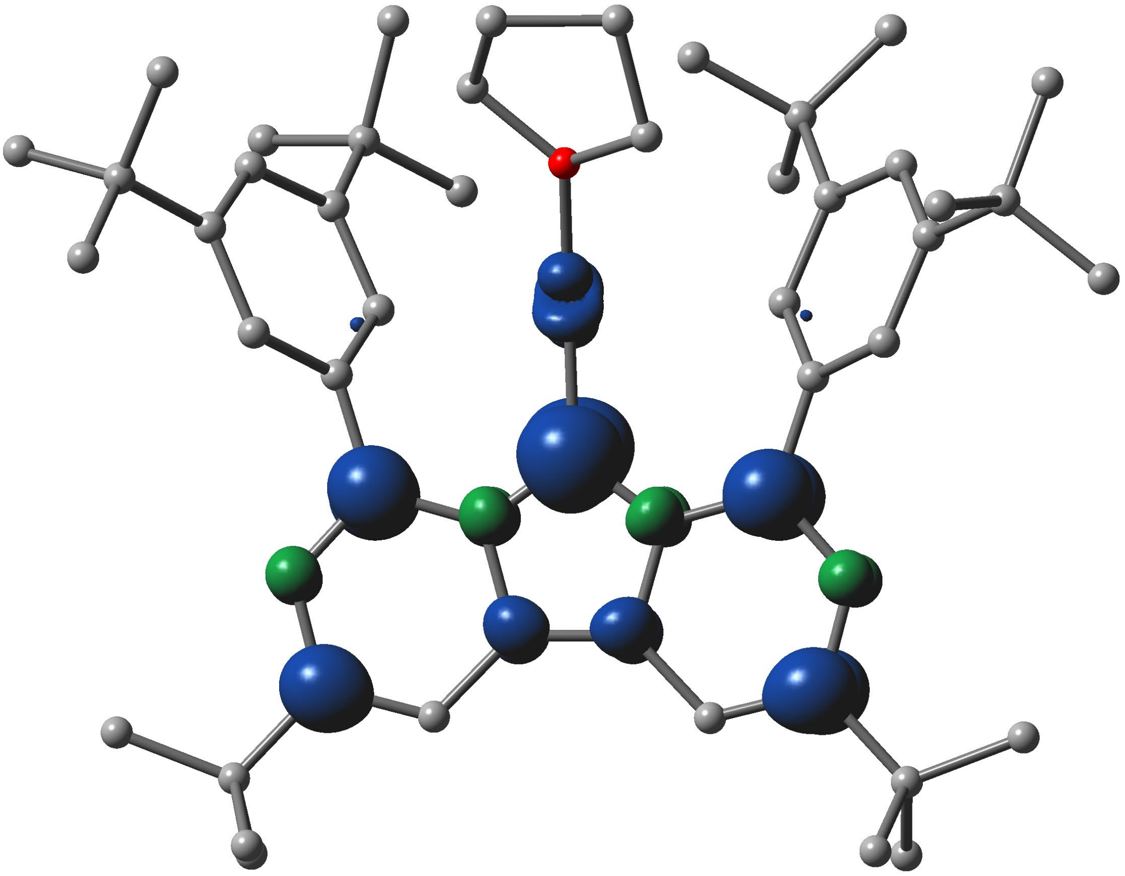

4.3.4 RCuNTf$_2$

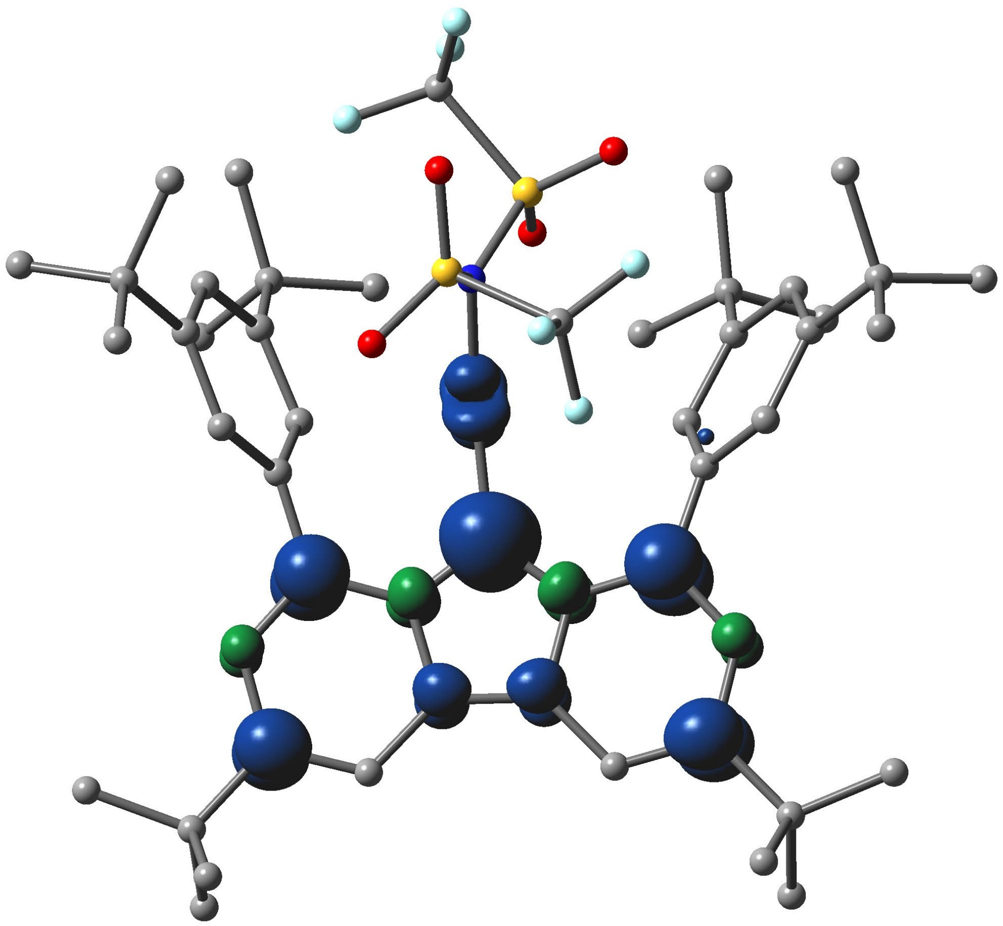

4.4 Details for TD-DFT computation of RCuOTf

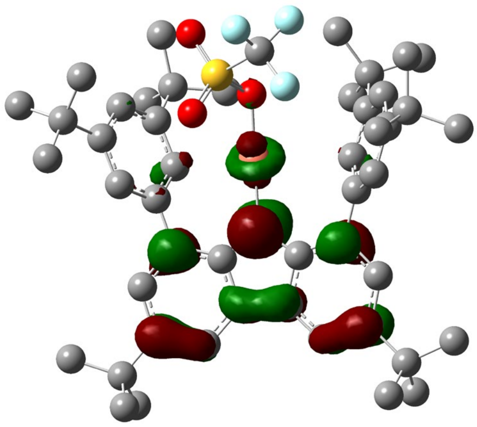

231-beta (unoccupied SOMO)

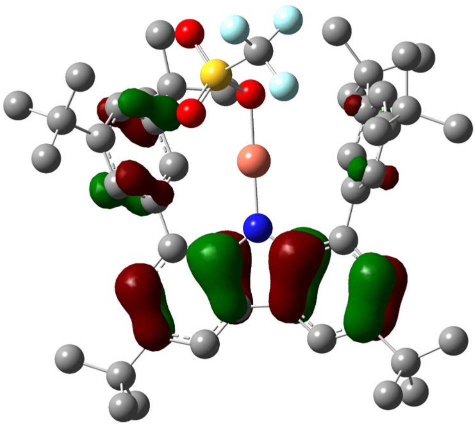

229-beta (occupied SOMO-2)

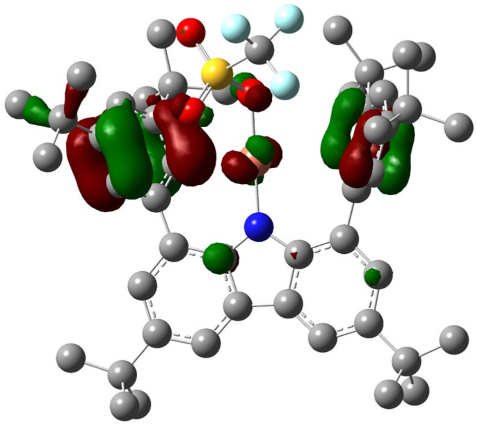

228-beta (occupied SOMO-3)

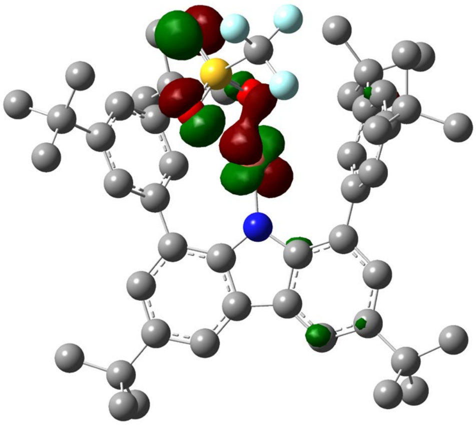

223-beta (occupied SOMO-8)

```
Excited State   :  2.035-A      1.5744 eV  787.52 nm  f=0.1170  <S**2>=0.785
    216A -> 239A        0.01276
    222A -> 238A       -0.01160
    225A -> 237A        0.01122
    226A -> 236A        0.01755
    227A -> 235A        0.01310
    228A -> 232A        0.04320
    229A -> 234A       -0.01371
    230A -> 233A       -0.01484
```

230A -> 234A 0.01049
230A -> 236A 0.01501
231A -> 233A -0.05871
231A -> 236A 0.02324
193B -> 231B 0.01644
197B -> 231B 0.03706
208B -> 231B -0.02129
209B -> 231B 0.02620
218B -> 231B -0.15815
218B -> 238B -0.01622
219B -> 231B -0.02028
220B -> 231B 0.02139
221B -> 231B -0.16586
221B -> 234B 0.01162
221B -> 235B 0.01008
222B -> 231B -0.06597
222B -> 238B -0.01382
223B -> 231B -0.02462
224B -> 231B -0.15692
224B -> 238B -0.01241
225B -> 231B 0.07431
226B -> 231B 0.12329
227B -> 231B 0.40832
227B -> 232B -0.03462
227B -> 233B 0.01232
227B -> 234B 0.01115
228B -> 231B 0.17490
228B -> 232B -0.02485
228B -> 233B -0.03309
229B -> 231B 0.80474
229B -> 232B 0.03694
229B -> 238B 0.01422
230B -> 231B 0.18071
230B -> 233B -0.01895
228A <- 232A 0.01865
231A <- 233A -0.02418
231A <- 236A 0.01153
227B <- 232B -0.01632
228B <- 232B -0.01130
229B <- 231B -0.01199
229B <- 232B 0.01467
230B <- 233B -0.01073

Excited State : 2.033-A 1.7070 eV 726.31 nm f=0.0082 <S**2>=0.783
224A -> 240A 0.01595
226A -> 238A -0.01143
228A -> 232A 0.01772
229A -> 233A -0.01004
230A -> 240A 0.02472
231A -> 232A 0.01082
231A -> 233A -0.01173
231A -> 234A -0.01252
231A -> 237A 0.01084
231A -> 240A -0.01253
195B -> 231B 0.01147
197B -> 231B -0.02989
198B -> 231B -0.03574
199B -> 231B 0.02323
206B -> 231B -0.02749
207B -> 231B -0.01684
208B -> 231B 0.01366
209B -> 231B -0.05013
210B -> 231B -0.03763
216B -> 231B -0.03638
217B -> 231B -0.01388
218B -> 231B 0.02396
219B -> 231B -0.17555
219B -> 234B 0.01319
219B -> 235B 0.01092

```
  219B -> 238B      -0.01423
  220B -> 231B      -0.15930
  220B -> 234B       0.01064
  220B -> 238B      -0.01383
  221B -> 231B       0.31168
  221B -> 234B      -0.01415
  221B -> 235B      -0.01502
  221B -> 236B      -0.01028
  221B -> 238B       0.02815
  221B -> 240B       0.01294
  222B -> 231B       0.31653
  222B -> 234B      -0.01201
  222B -> 235B      -0.01540
  222B -> 238B       0.02111
  222B -> 240B       0.01490
  223B -> 231B      -0.13071
  223B -> 240B       0.01211
  224B -> 231B       0.32814
  224B -> 234B      -0.01128
  224B -> 235B      -0.01292
  224B -> 238B       0.01929
  224B -> 240B       0.01356
  225B -> 231B       0.10764
  225B -> 236B      -0.01111
  226B -> 231B      -0.15729
  226B -> 235B       0.01060
  227B -> 231B      -0.36423
  227B -> 232B      -0.01536
  227B -> 238B      -0.01073
  228B -> 231B       0.61210
  228B -> 238B       0.01889
  229B -> 231B       0.23029
  229B -> 233B      -0.02310
  230B -> 231B      -0.03133
  230B -> 232B       0.01110
  230B -> 234B       0.02130
  230B -> 240B       0.01805
  230A <- 240A       0.01350
  230B <- 234B       0.01049
  230B <- 240B       0.01020

Excited State  :  2.044-A      2.3686 eV  523.46 nm  f=0.0225  <S**2>=0.794
    225A -> 232A      -0.01468
    225A -> 233A       0.01579
    226A -> 234A       0.01023
    228A -> 232A       0.01512
    228A -> 236A      -0.01837
    228A -> 239A       0.01023
    229A -> 233A      -0.02404
    230A -> 233A       0.01187
    230A -> 234A       0.01446
    231A -> 232A       0.04225
    231A -> 233A      -0.01541
    231A -> 236A       0.01431
    191B -> 231B      -0.02111
    192B -> 231B      -0.01114
    198B -> 231B      -0.01185
    202B -> 231B      -0.01818
    204B -> 231B       0.01242
    205B -> 231B       0.02809
    207B -> 231B      -0.01107
    208B -> 231B      -0.02674
    209B -> 231B      -0.01374
    210B -> 231B       0.01497
    214B -> 231B       0.02664
    216B -> 231B      -0.05135
    217B -> 231B       0.02701
    218B -> 231B       0.57652
    218B -> 234B      -0.02034
```

```
218B -> 235B      -0.01762
218B -> 236B      -0.01039
218B -> 238B       0.03177
218B -> 240B       0.01261
219B -> 231B       0.29614
219B -> 238B       0.01498
220B -> 231B       0.04459
221B -> 231B      -0.01640
221B -> 233B      -0.01313
222B -> 231B      -0.07171
222B -> 233B       0.01013
223B -> 231B      -0.55627
223B -> 232B      -0.01251
223B -> 238B      -0.01150
224B -> 231B      -0.20757
225B -> 231B       0.36409
225B -> 232B       0.02083
225B -> 233B      -0.02420
226B -> 231B       0.15319
226B -> 233B      -0.01107
226B -> 235B       0.01094
227B -> 231B      -0.07397
227B -> 237B      -0.01026
228B -> 231B       0.05493
228B -> 233B       0.01037
229B -> 231B      -0.02174
230B -> 231B       0.18322
230B -> 232B       0.01340
230B -> 234B       0.01576
230B -> 238B      -0.01586
```

4.5 Optimised Structures

4.5.1 RCuOTf

```
0 2
 Cu                 0.06040500   -0.43304800    0.42588500
 S                  0.16319500   -3.08496000   -0.91776500
 F                  2.66652000   -3.91887600   -0.97947400
 F                  1.58747900   -4.16710200   -2.82824500
 F                  2.25552000   -2.20853500   -2.22396700
 O                 -0.28346100   -4.41170400   -0.52023200
 O                 -0.63135700   -2.30387700   -1.86299200
 O                  0.63243200   -2.24373000    0.25709000
 N                 -0.42318500    1.38831200    0.31622300
 C                 -1.70230800    1.88860600    0.26281600
 C                 -2.90815900    1.17047400    0.16964300
 C                 -4.08142800    1.91848900   -0.02233700
 H                 -5.01279900    1.35739100   -0.09770500
 C                 -4.10063600    3.31685100   -0.08769100
 C                 -2.87452300    4.01197300    0.02347600
 H                 -2.85085900    5.10220800   -0.04338500
 C                 -1.69743300    3.30996100    0.19003800
 C                 -0.29431200    3.69272500    0.22227000
 C                  0.36830400    4.89896100    0.10429300
 H                 -0.20412700    5.82660300    0.03056200
 C                  1.77947100    4.92523600    0.05318800
 C                  2.46983500    3.70796500    0.11701700
 H                  3.55912200    3.69862100    0.07913900
 C                  1.83252800    2.46422000    0.25279500
 C                  0.42817600    2.46942000    0.30489600
 C                 -2.95551200   -0.29932700    0.24380500
 C                 -2.42993900   -0.97179100    1.35740700
 H                 -2.03490000   -0.37755600    2.18566200
 C                 -2.53802900   -2.36804400    1.46974700
 C                 -3.12029100   -3.05793000    0.40782400
 H                 -3.17553500   -4.14422400    0.45814100
 C                 -3.63617300   -2.41919200   -0.73100600
 C                 -3.56206800   -1.02979100   -0.78658200
 H                 -3.93833700   -0.48327100   -1.65107200
 C                 -2.11002400   -3.08445500    2.75293000
 C                 -0.80920800   -2.48715500    3.29457800
 H                 -0.92272500   -1.43240200    3.58907900
 H                 -0.01658900   -2.55821200    2.53751700
 H                 -0.48049300   -3.03403500    4.19184100
 C                 -1.87911200   -4.58055900    2.51687600
 H                 -1.48631900   -5.04349200    3.43518300
 H                 -1.15934800   -4.74611700    1.70136100
 H                 -2.81241600   -5.10734100    2.26644800
 C                 -3.23029000   -2.89717400    3.78871300
 H                 -2.96721200   -3.39740600    4.73504700
 H                 -4.17756000   -3.32509100    3.42562500
 H                 -3.40303600   -1.83054100    4.00225400
 C                 -4.25490300   -3.26230800   -1.84771700
 C                 -3.21214000   -4.25912100   -2.37707600
 H                 -2.83064200   -4.92099100   -1.58633800
 H                 -2.34366400   -3.72857000   -2.79050100
 H                 -3.66036400   -4.88816100   -3.16302500
 C                 -5.46215700   -4.02533100   -1.27928100
```

H	-5.16979400	-4.70081500	-0.46147600
H	-5.92970800	-4.63870800	-2.06619200
H	-6.22262300	-3.33108100	-0.88802200
C	-4.73204400	-2.39928200	-3.01714200
H	-5.51134200	-1.68308000	-2.71185500
H	-5.16311000	-3.04184000	-3.80003600
H	-3.89935900	-1.83863700	-3.46844800
C	-5.39423600	4.11168300	-0.27562600
C	-5.58951400	5.04615000	0.92925800
H	-6.51466600	5.63261700	0.81148500
H	-4.75672000	5.75687700	1.03880800
H	-5.66545400	4.47066400	1.86462000
C	-5.28940300	4.94505400	-1.56347900
H	-6.21296200	5.52547900	-1.71767900
H	-5.14183700	4.29641300	-2.44055700
H	-4.45191300	5.65765200	-1.52791400
C	-6.62163900	3.20527300	-0.38804300
H	-6.76802000	2.59894100	0.51879800
H	-6.55083600	2.52505400	-1.25052600
H	-7.52454300	3.81957400	-0.52452300
C	2.49789200	6.26998100	-0.07565100
C	2.13958700	7.14861700	1.13376600
H	1.05752100	7.33666400	1.19988100
H	2.64364900	8.12538600	1.05904800
H	2.45560600	6.67096600	2.07388900
C	2.04110500	6.96271800	-1.36997800
H	0.95722600	7.15120000	-1.37746900
H	2.28127300	6.34784500	-2.25093700
H	2.54801600	7.93445000	-1.48147500
C	4.01908700	6.11370200	-0.12496100
H	4.41293900	5.64461500	0.78955000
H	4.49039900	7.10384500	-0.21827600
H	4.34134500	5.51163200	-0.98830000
C	2.62526400	1.22026300	0.29484200
C	3.51516800	0.93841400	-0.74324200
H	3.56586800	1.63386200	-1.58307600
C	4.31048600	-0.21286300	-0.72517500
C	4.21207300	-1.04810000	0.39053300
H	4.83160500	-1.94331300	0.43418600
C	3.36672700	-0.77898600	1.47518700
C	2.54026700	0.34688800	1.39496600
H	1.88998500	0.62452000	2.22681400
C	5.30620000	-0.48161300	-1.85803400
C	6.50382400	0.46296400	-1.66126200
H	6.98768100	0.28884200	-0.68764200
H	6.18935400	1.51766100	-1.69644300
H	7.25496800	0.30490600	-2.45218300
C	4.66843300	-0.20871000	-3.22788700
H	3.78755200	-0.84478300	-3.39002500
H	5.39681600	-0.41862200	-4.02677500
H	4.35782800	0.84005300	-3.34245600
C	5.80658200	-1.92923000	-1.84425000
H	6.39875300	-2.15484100	-0.94451400
H	6.45909700	-2.10613700	-2.71270500
H	4.97075700	-2.64254600	-1.89335700
C	3.42305500	-1.68389700	2.70892400
C	4.85374900	-1.65880800	3.27250800
H	4.91670600	-2.28936100	4.17381900
H	5.15386300	-0.63560200	3.54800800
H	5.58716800	-2.04157000	2.54737500
C	3.05340100	-3.12330300	2.31840300
H	3.06412400	-3.77194000	3.20879500

H	3.76785400	-3.54097100	1.59367700
H	2.05999200	-3.16844800	1.85298600
C	2.47667900	-1.19899600	3.80747200
H	2.51887100	-1.88394200	4.66778500
H	1.43505500	-1.16903100	3.46318300
H	2.75197000	-0.19520400	4.16721700
C	1.77086900	-3.36761900	-1.78985400

4.5.2 $[RCu]^+$

1 2

Cu	0.98186500	-0.00022600	-0.00025400
N	-1.04127600	0.00027800	-0.00063400
C	-1.84342000	1.07367900	0.20384500
C	-1.39142800	2.38517100	0.43334700
C	-2.37454200	3.37496300	0.53275400
H	-2.05440800	4.40187100	0.70804000
C	-3.74672800	3.07494600	0.44929400
C	-4.16201400	1.72757100	0.24975300
H	-5.22722700	1.49446600	0.19264100
C	-3.22156200	0.72676600	0.12063100
C	-3.22172200	-0.72576600	-0.12150000
C	-4.16241100	-1.72638700	-0.25022400
H	-5.22755700	-1.49303300	-0.19292800
C	-3.74744800	-3.07390300	-0.44955300
C	-2.37533800	-3.37423300	-0.53314600
H	-2.05546000	-4.40126800	-0.70817800
C	-1.39198300	-2.38464700	-0.43416600
C	-1.84364500	-1.07295000	-0.20496600
C	0.07293800	2.57366900	0.52181000
C	0.77016300	3.46353800	-0.28047700
H	0.20466500	4.12468200	-0.94084100
C	2.18318100	3.48725800	-0.29764200
C	2.86709400	2.59139000	0.51882300
H	3.95615900	2.60373700	0.52292900
C	2.20710300	1.68886300	1.38753100
C	0.80328900	1.69542000	1.37328200
H	0.24067500	1.12727300	2.11688700
C	2.90282000	4.47196600	-1.22151300
C	2.49166200	4.18844000	-2.67542100
H	1.40861500	4.30241200	-2.83190200
H	2.77050900	3.16562300	-2.97156000
H	2.99879200	4.88939400	-3.35611300
C	4.42430900	4.35083600	-1.11935500
H	4.89927000	5.07365000	-1.79878300
H	4.77660900	3.34837300	-1.40889700
H	4.78814700	4.56864300	-0.10352200
C	2.49654800	5.90316600	-0.83641600
H	3.00180500	6.62981700	-1.49110800
H	2.77834200	6.12781500	0.20362200
H	1.41307100	6.06666400	-0.93613200
C	2.99633600	0.89571000	2.43736400
C	4.18243700	0.16585700	1.79822100
H	4.87095000	0.84678800	1.27775400
H	3.83073800	-0.58364400	1.07375300
H	4.76368100	-0.35658400	2.57311700
C	3.50647600	1.89965100	3.48446100
H	4.18005700	2.64331600	3.03303300
H	4.06327400	1.37542700	4.27659000
H	2.67114100	2.44121200	3.95384800
C	2.12379100	-0.15158800	3.13453800

H	1.30206400	0.29732000	3.71130900
H	2.73433200	-0.73251100	3.84119100
H	1.68888400	-0.86798900	2.41441900
C	-4.82027700	4.15592700	0.57861600
C	-5.66812100	4.17370300	-0.70501400
H	-6.44682800	4.94827400	-0.62963600
H	-6.17508700	3.21394600	-0.88398000
H	-5.04797800	4.39864200	-1.58619200
C	-5.71509500	3.83006300	1.78679300
H	-6.49511000	4.59946600	1.89484500
H	-5.12899300	3.80577600	2.71816200
H	-6.22224700	2.85961000	1.68039000
C	-4.22287500	5.54973300	0.78138200
H	-3.58767500	5.85258900	-0.06518800
H	-3.62826600	5.61341900	1.70551900
H	-5.03319000	6.28894200	0.86290200
C	-4.82123500	-4.15467700	-0.57843400
C	-5.71626000	-3.82884400	-1.78647400
H	-6.22318800	-2.85826800	-1.68011900
H	-6.49645400	-4.59810800	-1.89421000
H	-5.13036200	-3.80484000	-2.71797800
C	-5.66881100	-4.17203900	0.70538800
H	-6.17553800	-3.21214500	0.88427800
H	-5.04851900	-4.39695100	1.58646700
H	-6.44768900	-4.94646600	0.63031200
C	-4.22418000	-5.54864400	-0.78108200
H	-3.62988900	-5.61265900	-1.70540300
H	-5.03466600	-6.28771100	-0.86216500
H	-3.58875200	-5.85143600	0.06534300
C	0.07231000	-2.57361300	-0.52243000
C	0.76903000	-3.46360100	0.28019400
H	0.20313100	-4.12438400	0.94059200
C	2.18205200	-3.48782900	0.29775100
C	2.86648600	-2.59240100	-0.51874900
H	3.95554300	-2.60512800	-0.52262600
C	2.20701700	-1.68975700	-1.38773500
C	0.80320500	-1.69571200	-1.37381100
H	0.24101100	-1.12741200	-2.11761600
C	2.90108800	-4.47235800	1.22228700
C	2.49400700	-5.90360300	0.83826300
H	2.77567800	-6.12919100	-0.20160400
H	1.41043700	-6.06642500	0.93808900
H	2.99885400	-6.63004700	1.49350000
C	2.49005700	-4.18747800	2.67596100
H	2.76933600	-3.16453400	2.97128100
H	2.99688800	-4.88809600	3.35722100
H	1.40696000	-4.30087200	2.83254600
C	4.42265800	-4.35218900	1.12007600
H	4.78637700	-4.57083000	0.10438500
H	4.89719000	-5.07487200	1.79994100
H	4.77553000	-3.34974500	1.40900800
C	2.99680100	-0.89694900	-2.43738000
C	3.50644000	-1.90111000	-3.48452300
H	4.06365900	-1.37717400	-4.27654300
H	2.67082100	-2.44211100	-3.95405200
H	4.17948800	-2.64524000	-3.03306000
C	4.18326100	-0.16784000	-1.79805900
H	4.76473600	0.35451900	-2.57283800
H	4.87148200	-0.84920800	-1.27777300
H	3.83193000	0.58165800	-1.07341600
C	2.12487900	0.15091600	-3.13447600
H	2.73580200	0.73168900	-3.84092500

H	1.69022600	0.86739600	-2.41425000
H	1.30300000	-0.29742300	-3.71146900

4.5.3 $RCuSbF_6$

0 2

Cu	-0.15757100	-0.36381500	0.45569100
N	1.61022200	0.37515900	0.39907000
C	2.06774800	1.66550900	0.31698300
C	1.31681400	2.85626500	0.24619200
C	2.03703400	4.04405400	0.02473500
H	1.45577300	4.96447600	-0.02498200
C	3.42943300	4.09590800	-0.09639700
C	4.15811900	2.88536300	-0.00913900
H	5.24513400	2.88610100	-0.11810600
C	3.48969700	1.69636300	0.18826500
C	3.91270800	0.30204000	0.20985700
C	5.12207800	-0.34513800	0.05010100
H	6.04035000	0.23628300	-0.06029600
C	5.16446500	-1.75848400	0.00253700
C	3.96186800	-2.47133300	0.11318200
H	3.96699100	-3.56048200	0.07213800
C	2.71950500	-1.84716200	0.29426500
C	2.71201900	-0.44387900	0.34212400
C	-0.14909100	2.90305900	0.38661800
C	-0.77774800	2.31485900	1.49319600
H	-0.15590200	1.80931100	2.23370900
C	-2.15724200	2.45060900	1.68843600
C	-2.88448200	3.14742100	0.72118600
H	-3.96254400	3.24961700	0.85470800
C	-2.29759700	3.71725000	-0.41537300
C	-0.91567600	3.59854400	-0.55784300
H	-0.40756800	4.01847800	-1.42591700
C	-2.86164900	1.91950900	2.93891200
C	-1.98677600	0.90436500	3.67357600
H	-1.07678800	1.36118700	4.09195100
H	-1.68717800	0.09424000	2.99436200
H	-2.54142600	0.45406200	4.51049800
C	-4.18927900	1.24020700	2.57085700
H	-4.64385800	0.79402000	3.46921700
H	-4.04074700	0.45092200	1.82244200
H	-4.91926800	1.95342300	2.16129200
C	-3.14055600	3.10815700	3.87284100
H	-3.64957800	2.76799500	4.78942200
H	-3.78423400	3.85487400	3.38290000
H	-2.20504300	3.61061300	4.16454400
C	-3.18050200	4.43942900	-1.43603300
C	-4.26733800	3.47972600	-1.94595300
H	-4.89621700	3.09338200	-1.13048300
H	-3.81468700	2.61190800	-2.44381400
H	-4.92519500	3.99950500	-2.66076100
C	-3.83539300	5.65118500	-0.75430400
H	-4.46369600	5.34975200	0.09739300
H	-4.47823000	6.19075300	-1.46829800
H	-3.07454100	6.35349400	-0.37879000
C	-2.37547600	4.93240100	-2.63978700
H	-1.60251000	5.66125700	-2.34921200
H	-3.04587600	5.43122100	-3.35626400
H	-1.88799900	4.09702100	-3.16518600
C	4.18546300	5.40635700	-0.31887400
C	5.16328100	5.62593200	0.84695400

H	5.72261800	6.56405000	0.70330500
H	5.89738700	4.81045700	0.92912200
H	4.62467600	5.69154900	1.80476700
C	4.96810800	5.31534300	-1.63935200
H	5.52116500	6.25103100	-1.81851000
H	4.28799300	5.15130500	-2.48921500
H	5.70006700	4.49400100	-1.63101000
C	3.24758400	6.61253100	-0.39730500
H	2.67588700	6.74878400	0.53328100
H	2.53442800	6.52331300	-1.23097700
H	3.83535700	7.52832700	-0.56149900
C	6.51467700	-2.45564100	-0.17607000
C	7.43346300	-2.07793700	0.99724200
H	7.60740300	-0.99291800	1.05221300
H	8.41420100	-2.56720600	0.88611800
H	6.99799700	-2.39766300	1.95640600
C	7.14887800	-1.99311300	-1.49823300
H	7.32077700	-0.90662500	-1.51704200
H	6.50393700	-2.24636000	-2.35362000
H	8.12298400	-2.48594200	-1.64563800
C	6.38172400	-3.97936900	-0.21367000
H	5.95387700	-4.37682200	0.71929800
H	7.37544100	-4.43450500	-0.34201800
H	5.75371500	-4.31561300	-1.05277500
C	1.45891900	-2.61213400	0.37322100
C	1.07358800	-3.43153300	-0.68490900
H	1.71959800	-3.48236900	-1.56386400
C	-0.14593800	-4.12230200	-0.65966000
C	-0.92271000	-4.02372400	0.49420100
H	-1.86749900	-4.56198500	0.53893500
C	-0.54166100	-3.26479200	1.61115300
C	0.63422600	-2.50928600	1.51625200
H	1.00127100	-1.93337300	2.36831000
C	-0.57414900	-4.95346200	-1.87164000
C	0.43495000	-6.09688000	-2.06129800
H	0.46932700	-6.74497600	-1.17155200
H	1.45269900	-5.71807200	-2.24206900
H	0.15086800	-6.71723700	-2.92636000
C	-0.59340300	-4.06919400	-3.12883900
H	-1.32656200	-3.25704200	-3.03459200
H	-0.86276300	-4.67644300	-4.00753500
H	0.38648000	-3.61091900	-3.32830800
C	-1.97237800	-5.54856500	-1.69071400
H	-2.01502400	-6.25140500	-0.84389200
H	-2.25556600	-6.10779500	-2.59536500
H	-2.72171200	-4.75903700	-1.53079600
C	-1.37928600	-3.34537000	2.89091100
C	-1.47641100	-4.82042000	3.31661700
H	-2.04095100	-4.90451800	4.25885400
H	-0.47688600	-5.25457000	3.47537200
H	-1.99507300	-5.43184000	2.56401000
C	-2.79099100	-2.79877500	2.63277400
H	-3.41065000	-2.90905400	3.53695300
H	-3.28935500	-3.32580900	1.80702000
H	-2.76448800	-1.73694800	2.35743200
C	-0.73657900	-2.56950000	4.04217400
H	-1.37117200	-2.64006500	4.93858800
H	-0.62058000	-1.50186700	3.81042300
H	0.25383900	-2.97502400	4.30171600
F	-1.00704400	-1.31977300	-1.46383200
F	-2.20713100	-0.36263300	0.54882900
Sb	-2.87019700	-0.77460700	-1.28116900

F	-2.29333800	0.96264100	-1.82973000
F	-3.30015600	-1.28206900	-3.06043500
F	-3.33676700	-2.51670800	-0.64383500
F	-4.58190100	-0.09136100	-0.81173500

4.5.4 $RCuNTf_2$

0 2

Cu	-0.19583600	-1.05105600	-0.03391100
N	0.02453000	0.85310700	-0.18638100
C	1.16877800	1.60543100	-0.28479300
C	2.50356600	1.16616500	-0.23285300
C	3.50738900	2.14566100	-0.24984300
H	4.53964100	1.79534500	-0.21908700
C	3.24222800	3.52129200	-0.33180400
C	1.90039200	3.93605900	-0.37228900
H	1.65251900	5.00003000	-0.41289600
C	0.87551100	2.99925300	-0.34275500
C	-0.56815600	3.09124100	-0.27334300
C	-1.48054400	4.13886400	-0.20240400
H	-1.12090200	5.16946300	-0.26299800
C	-2.84929600	3.87679300	-0.03121300
C	-3.26666200	2.53589800	0.06253700
H	-4.32340700	2.30066700	0.19457300
C	-2.37972900	1.45898000	-0.02754500
C	-1.01873900	1.74816300	-0.18713500
C	2.83271500	-0.26237900	-0.10882200
C	2.23177400	-1.21887800	-0.95072400
H	1.60034500	-0.85411900	-1.76496600
C	2.59034500	-2.57610900	-0.87334600
C	3.51844800	-2.94969800	0.09804400
H	3.79304400	-4.00355100	0.17860800
C	4.11635900	-2.03094900	0.97238700
C	3.76548400	-0.68646100	0.84624800
H	4.19658400	0.06668600	1.50556200
C	2.02569800	-3.63496400	-1.82256400
C	0.99736000	-3.03869800	-2.78103600
H	1.43671900	-2.27269600	-3.43832600
H	0.16456600	-2.58206700	-2.22449400
H	0.57186200	-3.82531800	-3.42199100
C	1.34437300	-4.73931300	-1.00005200
H	0.89098200	-5.49052700	-1.66626500
H	0.55662900	-4.30707300	-0.36431400
H	2.05892600	-5.26213000	-0.34705500
C	3.17463300	-4.23201800	-2.64972000
H	2.78892800	-4.99699300	-3.34271300
H	3.93139100	-4.71001000	-2.00984800
H	3.67956000	-3.45407300	-3.24323300
C	5.10969300	-2.52997700	2.02508100
C	4.39377900	-3.52068600	2.95671800
H	4.00411500	-4.39078900	2.40760500
H	3.54418900	-3.03785000	3.46360000
H	5.08803300	-3.89322800	3.72702500
C	6.28124300	-3.23411700	1.32290700
H	5.94662100	-4.09717600	0.72839100
H	7.00662100	-3.60265300	2.06583300
H	6.80597500	-2.54293600	0.64533400
C	5.67320500	-1.38868700	2.87415300
H	6.21367100	-0.65120000	2.26097900
H	6.38369900	-1.79049100	3.61269800
H	4.88170000	-0.86177700	3.42850300

C	4.35759900	4.57158000	-0.37382500
C	4.25343600	5.37003000	-1.68275400
H	5.04356700	6.13694600	-1.73263800
H	3.28394300	5.88261400	-1.77205300
H	4.36231400	4.70724800	-2.55511400
C	4.20414500	5.52480000	0.82196600
H	4.99623200	6.29112200	0.80918600
H	4.27370900	4.97438900	1.77295100
H	3.23577600	6.04676400	0.80853400
C	5.75078000	3.94203800	-0.30627600
H	5.93604100	3.26604400	-1.15512800
H	5.89778300	3.37352300	0.62504200
H	6.51796700	4.73106600	-0.33730500
C	-3.83524300	5.04818400	0.04975400
C	-3.76733600	5.86050700	-1.25301100
H	-2.75906300	6.26336900	-1.43092900
H	-4.46625400	6.71202500	-1.21542300
H	-4.03441900	5.23482500	-2.11867400
C	-3.45870300	5.94963700	1.23620400
H	-2.44252000	6.35856300	1.13362300
H	-3.49876500	5.38829500	2.18249100
H	-4.15508100	6.80070300	1.31134000
C	-5.27874500	4.57960000	0.24645500
H	-5.62172000	3.94908200	-0.58818300
H	-5.94915000	5.45124400	0.30070700
H	-5.39999200	4.00970400	1.18052900
C	-2.82811300	0.05266200	0.06516300
C	-3.40071900	-0.43673000	1.23832700
H	-3.46936500	0.23668700	2.09546100
C	-3.85717800	-1.75867400	1.32930600
C	-3.77540100	-2.55599000	0.18677400
H	-4.15596500	-3.57617700	0.22692000
C	-3.22109100	-2.10254500	-1.01770700
C	-2.69784000	-0.80387600	-1.04846800
H	-2.27989800	-0.38674900	-1.96634500
C	-4.40130100	-2.27780900	2.66209100
C	-5.57714900	-1.39986000	3.11612000
H	-6.39570200	-1.42721200	2.38014200
H	-5.27965900	-0.34929300	3.24917600
H	-5.97152500	-1.75797500	4.08050000
C	-3.27198700	-2.21413900	3.70338500
H	-2.40886200	-2.81657600	3.38071200
H	-3.62221400	-2.59941500	4.67459200
H	-2.91820000	-1.18393800	3.85745800
C	-4.88900900	-3.72468100	2.56511800
H	-5.70736000	-3.83334700	1.83645500
H	-5.27093900	-4.05402600	3.54350400
H	-4.07758200	-4.41136500	2.27921500
C	-3.25012900	-3.01641700	-2.24546500
C	-4.71374400	-3.35483700	-2.57376000
H	-4.76405000	-4.00726100	-3.46030500
H	-5.29101800	-2.44178600	-2.78662600
H	-5.20944400	-3.88001900	-1.74394100
C	-2.48290600	-4.31101300	-1.93951100
H	-2.46902500	-4.96806500	-2.82393400
H	-2.94601600	-4.87269400	-1.11474700
H	-1.44579600	-4.09613200	-1.64977800
C	-2.63472800	-2.34709400	-3.47611600
H	-2.66500100	-3.03942700	-4.33126700
H	-1.58370600	-2.06984600	-3.31517300
H	-3.18801300	-1.44001100	-3.76368900
H	-0.37240000	-2.45702000	0.48266700

4.5.5 $RCuBF_4$

0 2			
Cu	0.62491300	-0.24898400	-0.06912500
N	-1.16354200	0.34495600	0.15688400
C	-2.26958900	-0.46983100	0.18648800
C	-2.28891900	-1.87362600	0.15451200
C	-3.54488200	-2.49058000	0.05560900
H	-3.55997600	-3.58005700	0.02548400
C	-4.74738800	-1.77080800	0.03316800
C	-4.69550200	-0.35794800	0.10726200
H	-5.61774900	0.22729900	0.09078300
C	-3.47533000	0.28397900	0.17945300
C	-3.05225200	1.68024000	0.18781100
C	-3.71250700	2.89334300	0.16727300
H	-4.80466200	2.91869500	0.17692600
C	-2.97232300	4.09712900	0.12142300
C	-1.57078000	4.02458500	0.08663000
H	-0.97791800	4.93778300	0.03841400
C	-0.86995300	2.81198900	0.10988800
C	-1.63136000	1.63827400	0.16825800
C	-1.03283500	-2.63994200	0.23768300
C	-0.15675000	-2.40184200	1.30933200
H	-0.46190300	-1.68970900	2.07749400
C	1.03511700	-3.12527100	1.44153700
C	1.34563100	-4.04352300	0.43443900
H	2.29274100	-4.57848900	0.49351300
C	0.50532500	-4.29215400	-0.65971100
C	-0.70139000	-3.59214500	-0.73067700
H	-1.38921400	-3.74370900	-1.56274100
C	1.91781500	-2.97645400	2.68384100
C	1.71857200	-1.60891400	3.34696800
H	0.70947800	-1.49062500	3.77013900
H	1.89445200	-0.79756200	2.62664800
H	2.43063600	-1.48661100	4.17715500
C	3.40428200	-3.13479000	2.34092800
H	4.01275600	-2.95876200	3.24144000
H	3.70935400	-2.42643900	1.56046300
H	3.64129500	-4.14911000	1.98724000
C	1.50607100	-4.07835600	3.67478100
H	2.11036600	-4.01448600	4.59439700
H	1.65361400	-5.07766300	3.23674500
H	0.44454900	-3.98621600	3.95348200
C	0.95127300	-5.26088500	-1.75745400
C	2.11826400	-4.61347500	-2.52009700
H	2.96475300	-4.38866300	-1.85721500
H	1.80815900	-3.65734600	-2.96797300
H	2.46650900	-5.28234700	-3.32393900
C	1.40834300	-6.58881800	-1.13622200
H	2.26886900	-6.45782300	-0.46436200
H	1.71666500	-7.28987900	-1.92778700
H	0.59595000	-7.05887200	-0.55984500
C	-0.17582200	-5.56206800	-2.74836000
H	-1.05693800	-5.99463000	-2.24821900
H	0.17417800	-6.28858300	-3.49745300
H	-0.49404700	-4.66105200	-3.29431300
C	-6.11075600	-2.45912700	-0.06026500
C	-6.94104000	-2.10361800	1.18380200
H	-7.92883700	-2.58879200	1.13400300
H	-7.10737200	-1.01970600	1.27327600

H	-6.43832300	-2.44366000	2.10220600
C	-6.83600300	-1.96596500	-1.32319800
H	-7.82177800	-2.45010400	-1.40940100
H	-6.25668100	-2.20512100	-2.22817700
H	-7.00069200	-0.87830800	-1.30740600
C	-5.98879500	-3.98216000	-0.13993000
H	-5.50298300	-4.40233300	0.75387100
H	-5.41863600	-4.30151900	-1.02564900
H	-6.99122400	-4.43050600	-0.21258500
C	-3.72266700	5.43060100	0.10377100
C	-4.57095500	5.54562600	1.38083100
H	-5.30876600	4.73331200	1.46052800
H	-5.12309300	6.49892500	1.38713000
H	-3.93555600	5.51197200	2.27915500
C	-4.63891000	5.47729100	-1.13004200
H	-5.38249600	4.66643800	-1.12383200
H	-4.05339200	5.38960500	-2.05809500
H	-5.18835400	6.43164800	-1.16040100
C	-2.77510600	6.63045000	0.04497800
H	-2.10632900	6.66773700	0.91831300
H	-3.35922400	7.56316600	0.03550800
H	-2.15508300	6.61929100	-0.86435000
C	0.60975900	2.75262300	0.02456200
C	1.22663000	2.89954800	-1.21680400
H	0.59706100	3.04675700	-2.09724500
C	2.62190700	2.84947400	-1.34189400
C	3.36728700	2.67150900	-0.17702600
H	4.45266200	2.62396400	-0.25216500
C	2.78289500	2.52921100	1.09065300
C	1.38938300	2.55143100	1.17404500
H	0.88087900	2.43731200	2.13198600
C	3.25931100	3.00181900	-2.72536200
C	2.94703200	4.40868800	-3.25938100
H	3.35990600	5.18384600	-2.59471900
H	1.86331000	4.58205000	-3.34583000
H	3.38864200	4.54468900	-4.25982600
C	2.67365300	1.94485200	-3.67535000
H	2.85803000	0.93365500	-3.28785300
H	3.13942700	2.03183700	-4.67007500
H	1.58809600	2.06789200	-3.80832600
C	4.77763800	2.81889500	-2.68043100
H	5.26401200	3.58179100	-2.05238000
H	5.19180000	2.91825100	-3.69559000
H	5.04300100	1.82253100	-2.30031300
C	3.68507800	2.39217900	2.32139600
C	4.51719100	3.67809800	2.45693500
H	5.17141700	3.61951700	3.34179900
H	3.86655300	4.55966000	2.56830300
H	5.15744300	3.84190200	1.57729000
C	4.62618900	1.18862600	2.15939100
H	5.30573900	1.12276700	3.02419700
H	5.24269400	1.26700500	1.25216600
H	4.06015000	0.25106300	2.08257100
C	2.87758400	2.20479900	3.60741100
H	3.56132400	2.10003300	4.46372700
H	2.25634400	1.29774700	3.56834600
H	2.22219300	3.06579800	3.81127900
F	1.83795000	-0.97950500	-1.66469200
F	2.63403300	-0.55452700	0.38659300
B	3.07137900	-1.01106100	-0.92478200
F	3.97230000	-0.12677600	-1.44538500
F	3.55561900	-2.28406700	-0.83037000

4.5.6 $RCuOSO_3Et$

```
0 2
 Cu                 0.39879800    0.10813100    0.40018100
 S                  3.10633500   -0.22944000   -0.87335700
 O                  4.16938900   -1.12988300   -0.44446900
 O                  2.26684100   -0.64895100   -1.99361700
 O                  2.28687500    0.29949400    0.28298300
 N                 -1.48331400   -0.03825800    0.27191500
 C                 -2.21868400   -1.19420600    0.17513000
 C                 -1.73599700   -2.50762700    0.05540900
 C                 -2.68281300   -3.51399300   -0.19286000
 H                 -2.30372000   -4.53130200   -0.29013200
 C                 -4.05884600   -3.26731100   -0.28306100
 C                 -4.51656300   -1.93813600   -0.13432700
 H                 -5.58199300   -1.71012800   -0.21560100
 C                 -3.61206500   -0.91785300    0.08732900
 C                 -3.72211000    0.53098000    0.17048800
 C                 -4.77248300    1.42442400    0.07277800
 H                 -5.79405700    1.05037300   -0.02828300
 C                 -4.52067200    2.81427200    0.07740200
 C                 -3.19216800    3.25273400    0.17225500
 H                 -2.96751500    4.31938200    0.16961900
 C                 -2.10451700    2.37574900    0.29049000
 C                 -2.38530000    1.00067000    0.28810500
 C                 -0.30268800   -2.82277300    0.18194800
 C                  0.38494300   -2.50285300    1.36595500
 H                 -0.17579600   -2.03621000    2.17786900
 C                  1.71736300   -2.89670700    1.54610400
 C                  2.35476700   -3.52995300    0.47474100
 H                  3.40738200   -3.79594900    0.58161000
 C                  1.70647800   -3.84623200   -0.72671800
 C                  0.36007000   -3.49908800   -0.84655300
 H                 -0.19714400   -3.72421200   -1.75556800
 C                  2.46612400   -2.70264900    2.86850100
 C                  1.55221300   -2.14408500    3.96167900
 H                  0.70944800   -2.82084100    4.17334600
 H                  1.14306900   -1.15895600    3.69760100
 H                  2.12260400   -2.01874700    4.89468900
 C                  3.64880500   -1.74696500    2.65873100
 H                  4.18955900   -1.59832400    3.60758400
 H                  3.30943600   -0.77275300    2.28383900
 H                  4.35479400   -2.12984300    1.90925600
 C                  2.98865500   -4.06638000    3.34905400
 H                  3.51041000   -3.95314700    4.31286200
 H                  3.70191900   -4.50686400    2.63730500
 H                  2.16318200   -4.78232600    3.48732700
 C                  2.47021100   -4.57388400   -1.83619500
 C                  3.65600400   -3.71316700   -2.29488200
 H                  4.34374100   -3.48207300   -1.46985400
 H                  3.30316300   -2.74898300   -2.68689100
 H                  4.21948600   -4.24131400   -3.08133700
 C                  2.98062000   -5.91626600   -1.28855200
 H                  3.66754300   -5.77760000   -0.44041100
 H                  3.52805600   -6.46330900   -2.07290600
 H                  2.14650300   -6.54953300   -0.94694000
 C                  1.58247900   -4.85387900   -3.05054700
 H                  0.72262000   -5.49395000   -2.79656900
 H                  2.16715400   -5.37731800   -3.82250000
 H                  1.20484900   -3.92123100   -3.49651900
```

C	-5.07650600	-4.38212800	-0.53437200
C	-6.06143400	-4.44030900	0.64443600
H	-6.80636800	-5.23542600	0.48106800
H	-6.60770600	-3.49376500	0.77212600
H	-5.53383600	-4.65345100	1.58682400
C	-5.84283400	-4.07967600	-1.83251900
H	-6.58212400	-4.87171200	-2.03227200
H	-5.15594300	-4.02739500	-2.69115300
H	-6.38604600	-3.12446500	-1.77844800
C	-4.41273700	-5.75327700	-0.67666500
H	-3.86775500	-6.04155000	0.23511000
H	-3.70927500	-5.78177500	-1.52277000
H	-5.18116100	-6.51978700	-0.85921300
C	-5.69836500	3.78591400	-0.02927300
C	-6.64427500	3.56170500	1.16155200
H	-7.03997500	2.53533300	1.18523900
H	-7.50331800	4.24901000	1.10227200
H	-6.12517300	3.74346200	2.11517200
C	-6.45214000	3.52284300	-1.34315900
H	-6.84853200	2.49783300	-1.39388800
H	-5.79184800	3.67090000	-2.21146900
H	-7.30474800	4.21398100	-1.43878200
C	-5.24793100	5.24810400	-0.01844800
H	-4.72057800	5.50765500	0.91215400
H	-6.12608000	5.90701400	-0.09609800
H	-4.58538000	5.47906400	-0.86657800
C	-0.71302700	2.86050100	0.34574400
C	-0.19766400	3.59951500	-0.71857600
H	-0.84504700	3.79276400	-1.57645400
C	1.13496000	4.02595800	-0.72102200
C	1.91599600	3.73424900	0.39973400
H	2.95381700	4.05874400	0.41541500
C	1.43328500	3.01018500	1.49513600
C	0.11191000	2.55055900	1.44237600
H	-0.32706300	2.00782000	2.28195400
C	1.71328900	4.69516000	-1.96838100
C	0.81211900	5.84925300	-2.42781000
H	0.72968000	6.62207900	-1.64751700
H	-0.20419400	5.51082900	-2.67759600
H	1.23082000	6.31976700	-3.33149800
C	1.79084000	3.61841600	-3.06542500
H	2.36785100	2.74949700	-2.71471000
H	2.26836700	4.02342200	-3.97285300
H	0.78855200	3.25846700	-3.34353100
C	3.11616100	5.25290300	-1.71983400
H	3.11644600	6.00654400	-0.91668200
H	3.49144900	5.73742900	-2.63433700
H	3.82852100	4.46045800	-1.45098200
C	2.32797000	2.76350100	2.71090100
C	2.28907800	4.02110800	3.59369300
H	2.92569500	3.88703000	4.48341800
H	1.26390500	4.23693300	3.93335000
H	2.65341900	4.90172800	3.04227600
C	3.77551600	2.48387600	2.27964500
H	4.37726200	2.20001200	3.15688000
H	4.25358900	3.37016800	1.83666100
H	3.81264300	1.66949400	1.54340800
C	1.82994100	1.56446400	3.51887000
H	2.53560900	1.32858200	4.32946700
H	1.75321700	0.67756300	2.87414300
H	0.84950100	1.74726700	3.98440600
O	3.85331200	1.16097400	-1.32399300

C	4.91201700	0.97563600	-2.26345300
H	5.64689500	0.27252600	-1.83816000
H	4.50452900	0.52441500	-3.18558900
C	5.53905300	2.31702600	-2.54415000
H	4.82218300	3.00206300	-3.01789400
H	5.89697600	2.77842200	-1.61163200
H	6.39759200	2.19433200	-3.22123800

4.5.7 $RCuN_3$

0 2

Cu	0.23971000	-0.77992900	0.07591400
N	-0.36014200	0.99927000	-0.12210800
C	0.41641000	2.13245200	-0.18604400
C	1.81895700	2.21048900	-0.16008200
C	2.38957600	3.48937900	-0.08697100
H	3.47809300	3.54468800	-0.06198000
C	1.62680200	4.66579200	-0.08720900
C	0.21986700	4.55741100	-0.15244000
H	-0.40140000	5.45628600	-0.15005500
C	-0.37893500	3.31133100	-0.19787100
C	-1.75731100	2.84383300	-0.18457800
C	-2.99268300	3.46842900	-0.16675600
H	-3.04875200	4.55915400	-0.20029800
C	-4.17119000	2.69771000	-0.09392900
C	-4.05489000	1.29884500	-0.02774000
H	-4.94942600	0.68008400	0.04346200
C	-2.82306100	0.63517100	-0.04775400
C	-1.67003500	1.42650600	-0.13348200
C	2.63030700	0.98353600	-0.22092700
C	2.41925600	0.07859500	-1.27001800
H	1.70535500	0.35049100	-2.05096200
C	3.17388200	-1.09955600	-1.37741700
C	4.09650500	-1.36929500	-0.36659100
H	4.65505200	-2.30333800	-0.40163000
C	4.32582300	-0.49130200	0.70775100
C	3.59547800	0.69647400	0.75299600
H	3.73218400	1.40848300	1.56722700
C	2.99678000	-1.99830000	-2.60357400
C	1.50339700	-2.22718400	-2.87304000
H	0.98515700	-1.29907400	-3.15930800
H	0.99866400	-2.62747100	-1.98136600
H	1.36703000	-2.94193000	-3.69930500
C	3.67549000	-3.35813200	-2.42427400
H	3.48816000	-3.98473400	-3.30969900
H	3.29542200	-3.88845000	-1.53813300
H	4.76710400	-3.25815800	-2.32365900
C	3.62618100	-1.28400400	-3.81104400
H	3.50787900	-1.89326000	-4.72177100
H	4.70182200	-1.11301100	-3.64994600
H	3.15349200	-0.30628000	-3.99232300
C	5.31943900	-0.88199000	1.80501200
C	4.71869800	-2.04857400	2.60589800
H	4.48613800	-2.91198000	1.96566800
H	3.77825700	-1.74319600	3.09074400
H	5.41952900	-2.37467400	3.39154900
C	6.65208600	-1.31754900	1.17752000
H	6.53876900	-2.20245400	0.53488400
H	7.37402400	-1.57845600	1.96736200
H	7.08722800	-0.50913400	0.56925600
C	5.60179400	0.27803300	2.76273500

H	6.00730900	1.15524100	2.23421700
H	6.34386700	-0.03359000	3.51357300
H	4.69887700	0.59047000	3.30894100
C	2.26527600	6.05595700	-0.02870900
C	1.87232600	6.84597300	-1.28737700
H	2.31939600	7.85269600	-1.26269200
H	0.78223100	6.96799100	-1.37304300
H	2.22544500	6.33625300	-2.19699000
C	1.75566800	6.79162400	1.22136300
H	2.20279000	7.79675800	1.28231100
H	2.02249400	6.24160900	2.13689500
H	0.66241000	6.91432100	1.21052000
C	3.79234000	5.99118000	0.04187800
H	4.22317800	5.49925300	-0.84351800
H	4.13829600	5.45365600	0.93804300
H	4.20513900	7.01046700	0.08746500
C	-5.52780400	3.40757400	-0.08069200
C	-5.67899900	4.22955200	-1.37085000
H	-4.89036900	4.99046400	-1.46880000
H	-6.64923700	4.75164200	-1.38084000
H	-5.63028300	3.57980000	-2.25820000
C	-5.59629500	4.34337500	1.13705600
H	-4.80660800	5.10904000	1.11312100
H	-5.48597300	3.77660000	2.07435000
H	-6.56598800	4.86583900	1.16418800
C	-6.69747300	2.42464300	0.00273100
H	-6.71802200	1.73848200	-0.85766400
H	-7.64824300	2.97919900	0.00702800
H	-6.66175200	1.82224100	0.92325200
C	-2.71656500	-0.84063800	0.07253900
C	-2.75793600	-1.42558700	1.33723200
H	-2.86874100	-0.77390500	2.20698900
C	-2.62430800	-2.81049800	1.49857900
C	-2.49746200	-3.58559800	0.34741400
H	-2.40188300	-4.66582000	0.44901300
C	-2.45914800	-3.03552800	-0.94214200
C	-2.54707000	-1.64771700	-1.06326600
H	-2.50730300	-1.16358600	-2.03965700
C	-2.57689600	-3.40372200	2.90813900
C	-3.89153800	-3.09516800	3.63931000
H	-4.74882500	-3.54804400	3.11700000
H	-4.07542800	-2.01253400	3.71042700
H	-3.86399400	-3.49687600	4.66506000
C	-1.39657300	-2.77023100	3.66303200
H	-0.45808500	-2.92556700	3.11008000
H	-1.29926000	-3.21534100	4.66632700
H	-1.52995700	-1.68536700	3.79076600
C	-2.37367500	-4.91977300	2.88748000
H	-3.19604800	-5.43777400	2.36980800
H	-2.33845800	-5.30396600	3.91842900
H	-1.42676400	-5.19420500	2.39800300
C	-2.34431700	-3.96898600	-2.15059400
C	-3.63976500	-4.78971500	-2.25601800
H	-3.59016600	-5.48034400	-3.11352500
H	-4.51213000	-4.13215200	-2.39417900
H	-3.81198500	-5.38919100	-1.34929500
C	-1.15206300	-4.92101000	-1.96872600
H	-1.04879600	-5.57343900	-2.85038800
H	-1.27184100	-5.57144200	-1.09042200
H	-0.21426200	-4.36313100	-1.84050800
C	-2.15220800	-3.19496500	-3.45642200
H	-2.04783900	-3.89924800	-4.29597300

H	-1.24607200	-2.57176300	-3.42889900
H	-3.01204800	-2.54447800	-3.67831900
N	0.61095400	-2.51159600	0.62662500
N	1.55223900	-3.23601200	0.71795900
N	2.41676800	-3.99008500	0.81796700

4.5.8 RCuO^{t}Bu^{F}

0 2

Cu	0.00584800	-0.26226700	0.34140400
O	-0.28854700	-2.07521300	0.09152000
N	0.20056200	1.60992300	0.32969300
C	-0.82309100	2.53850500	0.29918600
C	-2.21806200	2.33341700	0.29239700
C	-3.02837500	3.47501500	0.14793500
H	-4.10527700	3.30892500	0.15739700
C	-2.53297400	4.77714800	0.03052400
C	-1.13441100	4.95493200	0.04504100
H	-0.69967700	5.95185600	-0.05951500
C	-0.30489600	3.85894200	0.17524500
C	1.13739000	3.71389300	0.14668900
C	2.16560400	4.61967400	-0.02623800
H	1.93737400	5.68355000	-0.12553700
C	3.49823200	4.16370800	-0.09033400
C	3.72798400	2.78973100	0.03472500
H	4.74998000	2.41178900	0.02002900
C	2.70999000	1.83817700	0.22554800
C	1.38551300	2.31796100	0.26307700
C	-2.87519300	1.01950100	0.41647300
C	-2.57815100	0.14985200	1.47295600
H	-1.83937700	0.46392500	2.21465100
C	-3.30014500	-1.04196000	1.64995700
C	-4.28195800	-1.35040400	0.71096100
H	-4.83600100	-2.28221800	0.82076900
C	-4.59309200	-0.51547700	-0.37343800
C	-3.88722900	0.67700900	-0.49502700
H	-4.08302300	1.35813400	-1.32302200
C	-3.07801700	-1.91428100	2.88759600
C	-1.58856200	-1.97851900	3.22865900
H	-1.20397600	-0.99901800	3.54921800
H	-1.00817600	-2.31744900	2.35842600
H	-1.41221000	-2.67453500	4.06329900
C	-3.58208300	-3.34619300	2.68145200
H	-3.34758800	-3.95036500	3.57116100
H	-3.10314100	-3.81928200	1.81261300
H	-4.67314800	-3.38897400	2.54436500
C	-3.84689100	-1.27606300	4.05604800
H	-3.70306200	-1.86201500	4.97855300
H	-4.92601500	-1.23099500	3.84145700
H	-3.49929200	-0.24863200	4.24634600
C	-5.67684500	-0.94422600	-1.36609000
C	-5.30174700	-2.30054800	-1.98459800
H	-5.17511200	-3.08176000	-1.22106700
H	-4.35771200	-2.23254700	-2.54283500
H	-6.09213600	-2.63218800	-2.67682400
C	-7.01365100	-1.07117400	-0.61846200
H	-6.96311000	-1.82200000	0.18422500
H	-7.81324500	-1.37745600	-1.31198900
H	-7.30330200	-0.11134000	-0.16294000
C	-5.84897600	0.06851800	-2.49971500
H	-6.15615700	1.05736200	-2.12491200

H	-6.62932800	-0.27873600	-3.19403200
H	-4.92002100	0.19027500	-3.07763300
C	-3.44602600	5.99632300	-0.10998500
C	-3.19352400	6.95207000	1.06707500
H	-3.83768100	7.84186600	0.98240200
H	-2.14966900	7.29852800	1.09883700
H	-3.41334100	6.45982200	2.02688700
C	-3.12727600	6.71194700	-1.43279800
H	-3.77356800	7.59590300	-1.55401700
H	-3.29542600	6.04392900	-2.29148100
H	-2.08302100	7.05583800	-1.47459600
C	-4.92769700	5.61499100	-0.11390800
H	-5.22614200	5.12029000	0.82295300
H	-5.17592400	4.94559700	-0.95181500
H	-5.54348900	6.52095400	-0.22050900
C	4.63111300	5.17340500	-0.28346600
C	4.62261400	6.16987800	0.88686100
H	3.67222300	6.72056500	0.95136700
H	5.42920500	6.91037900	0.76472900
H	4.77582800	5.65106000	1.84562000
C	4.41041700	5.92783700	-1.60467500
H	3.45506900	6.47344700	-1.61339500
H	4.40795600	5.23297000	-2.45849900
H	5.21509000	6.66345300	-1.76324900
C	6.00485300	4.50184200	-0.33642700
H	6.23621100	3.96696000	0.59732200
H	6.78496600	5.26429500	-0.48277400
H	6.07937900	3.78765600	-1.17080000
C	3.08091100	0.42304800	0.39687800
C	3.99406500	-0.16890700	-0.47885900
H	4.34542900	0.41299200	-1.33353100
C	4.42762400	-1.48561300	-0.29684200
C	3.95204100	-2.17450700	0.82102100
H	4.28071800	-3.19915800	0.98940800
C	3.06182900	-1.60860300	1.74322100
C	2.60445500	-0.31112100	1.49826300
H	1.95034000	0.19430400	2.21086800
C	5.40371300	-2.11442300	-1.29598200
C	6.76303200	-1.41143700	-1.15478400
H	7.16259400	-1.52793400	-0.13536500
H	6.68187800	-0.33328300	-1.36239900
H	7.49380200	-1.83718400	-1.86138200
C	4.88206600	-1.93662000	-2.73037100
H	3.90322600	-2.41947300	-2.85898200
H	5.58780800	-2.38735000	-3.44596600
H	4.76971300	-0.87698900	-3.00287000
C	5.59058300	-3.61263300	-1.04424700
H	6.04410000	-3.81428000	-0.06175900
H	6.26041100	-4.03793200	-1.80706600
H	4.63114800	-4.14982700	-1.09963100
C	2.63369100	-2.41873000	2.96847500
C	3.87522000	-2.93055400	3.71428200
H	3.56993700	-3.50159600	4.60551100
H	4.51331400	-2.09570000	4.04419500
H	4.48782000	-3.59843600	3.09115200
C	1.78299900	-3.60732300	2.49588200
H	1.39557900	-4.17109200	3.35985500
H	2.37289700	-4.30052800	1.87827500
H	0.93722400	-3.26957400	1.88136800
C	1.81843700	-1.56857700	3.94237100
H	1.51092800	-2.17688800	4.80635800
H	0.90394300	-1.18384300	3.47406000

H	2.40102200	-0.71413300	4.32134400
C	-0.35345500	-2.80855100	-1.02421200
C	-1.14442200	-4.11489600	-0.70911400
C	-1.08281400	-2.04904300	-2.17948900
C	1.07768300	-3.17883400	-1.52253200
F	-0.28517700	-1.11011300	-2.69967700
F	-2.15700900	-1.42743600	-1.71172400
F	-2.45296200	-3.85729400	-0.61495200
F	-0.75719900	-4.62453800	0.45320300
F	-0.98376700	-5.05269600	-1.64264400
F	-1.46971600	-2.84929300	-3.17227200
F	1.09444500	-3.68655100	-2.75681100
F	1.65109400	-4.07079900	-0.71319100
F	1.83819200	-2.08934500	-1.52510400

4.5.9 RCuCl

0 2

Cu	-0.15664300	-0.85368200	-0.12392500
Cl	-0.55741700	-2.83931700	-0.85484700
N	0.19892100	0.99545900	0.12386100
C	-0.73579300	2.00257000	0.18894100
C	-2.13441000	1.87255900	0.16056000
C	-2.88576100	3.05507900	0.09096600
H	-3.97042700	2.95002300	0.06210100
C	-2.30365300	4.33007100	0.09894800
C	-0.89512700	4.42986200	0.17201400
H	-0.41298100	5.41028200	0.17893000
C	-0.12188200	3.28532400	0.21434500
C	1.31144500	3.02392700	0.21096000
C	2.44163900	3.82093700	0.19835600
H	2.33929400	4.90820800	0.23259500
C	3.72121000	3.22864900	0.12697200
C	3.81055100	1.82869000	0.05751100
H	4.78558200	1.34642400	-0.01093500
C	2.68759700	0.99307400	0.07305900
C	1.43175700	1.60906900	0.15571100
C	-2.76025600	0.54041400	0.21839400
C	-2.43165100	-0.32080800	1.27627200
H	-1.75305300	0.04761000	2.04665800
C	-3.00027700	-1.59600900	1.37266700
C	-3.87721600	-1.98701300	0.36073600
H	-4.30223100	-2.99031900	0.39905700
C	-4.19882200	-1.16923100	-0.73077700
C	-3.64773800	0.11243400	-0.77468200
H	-3.85702200	0.78653000	-1.60561100
C	-2.67511200	-2.53101800	2.53927000
C	-1.33797300	-2.15114200	3.18221800
H	-1.37617100	-1.17371500	3.68651700
H	-0.54061400	-2.11857900	2.42323500
H	-1.05461000	-2.89703700	3.93997800
C	-2.57498800	-3.98453400	2.05469300
H	-2.22801400	-4.63098000	2.87584900
H	-1.87009500	-4.06368600	1.21343000
H	-3.54821100	-4.37855900	1.72592500
C	-3.79382300	-2.41005700	3.58491500
H	-3.59569700	-3.07393100	4.44231000
H	-4.76764700	-2.68978800	3.15385200
H	-3.87641100	-1.37806400	3.96055900
C	-5.06449300	-1.73423900	-1.85847900
C	-4.31977200	-2.92928700	-2.47729300

H	-4.17905200	-3.74202700	-1.74969700
H	-3.31721000	-2.63293100	-2.82072300
H	-4.88530300	-3.33299700	-3.33271000
C	-6.41765100	-2.19415000	-1.29682900
H	-6.30063900	-2.96549700	-0.52129600
H	-7.03864400	-2.62305200	-2.09951400
H	-6.96806200	-1.35038800	-0.85188100
C	-5.32412300	-0.70196200	-2.95736600
H	-5.84686300	0.18670700	-2.57009700
H	-5.95781900	-1.14477200	-3.74094000
H	-4.38939700	-0.37295800	-3.43645600
C	-3.13819800	5.61183100	0.04247600
C	-2.87217200	6.44347500	1.30762800
H	-3.46199500	7.37377000	1.28455600
H	-1.81235500	6.72379000	1.40112400
H	-3.15224400	5.88244100	2.21243800
C	-2.73360200	6.42069800	-1.20085700
H	-3.32290800	7.34968400	-1.26020700
H	-2.91141500	5.84262100	-2.12068700
H	-1.67028900	6.70247600	-1.18200800
C	-4.63886600	5.32488800	-0.03887100
H	-4.99881100	4.77122200	0.84167700
H	-4.89683800	4.74682200	-0.93939300
H	-5.19569500	6.27321300	-0.08350000
C	4.95930000	4.12885600	0.11920600
C	4.98570800	4.95914600	1.41271100
H	4.09414900	5.59625500	1.51146100
H	5.86909100	5.61745500	1.42647000
H	5.03127200	4.30604800	2.29775100
C	4.89216000	5.06899100	-1.09537200
H	3.99969400	5.71189500	-1.07076400
H	4.86730700	4.49570900	-2.03484800
H	5.77564600	5.72686600	-1.11834100
C	6.26014700	3.32769400	0.03513600
H	6.37947100	2.64895800	0.89335100
H	7.11950700	4.01540300	0.03478500
H	6.31452400	2.72990600	-0.88744400
C	2.78573800	-0.48170700	-0.04539900
C	2.94874900	-1.05679400	-1.30443700
H	3.02014100	-0.39727100	-2.17221200
C	2.96605100	-2.44826800	-1.46540800
C	2.87995800	-3.23191000	-0.31652100
H	2.89151000	-4.31619300	-0.41782900
C	2.73759300	-2.69010300	0.96883000
C	2.66373500	-1.30141300	1.08766900
H	2.52711400	-0.82481300	2.05901500
C	3.03415400	-3.04535300	-2.87256900
C	4.38831900	-2.69422500	-3.50550000
H	5.21970100	-3.11790100	-2.92037100
H	4.53874300	-1.60517700	-3.56256500
H	4.45027300	-3.09562100	-4.53005900
C	1.89127600	-2.45864600	-3.71781600
H	0.92137000	-2.64319900	-3.23122600
H	1.88445200	-2.91917900	-4.71888900
H	1.99897400	-1.37218800	-3.85590500
C	2.87237200	-4.56647700	-2.85268000
H	3.68316500	-5.06149700	-2.29570000
H	2.89529400	-4.95627500	-3.88194000
H	1.90991200	-4.85444000	-2.40285700
C	2.65250700	-3.63383200	2.17101400
C	3.96227200	-4.43094400	2.27303400
H	3.92138000	-5.12841600	3.12534800

H	4.82249400	-3.75922300	2.42004000
H	4.15002000	-5.02289800	1.36481400
C	1.47473200	-4.60094700	1.97386800
H	1.36116400	-5.25106600	2.85639700
H	1.62056300	-5.24882500	1.09737700
H	0.53557500	-4.05465400	1.81227100
C	2.45109800	-2.87456500	3.48397500
H	2.39099200	-3.58713000	4.32092100
H	1.51923500	-2.29073800	3.47988400
H	3.28716700	-2.18897600	3.69193000

4.5.10 RCuOTos

0 2

Cu	0.00016900	-0.31517000	0.51642900
S	1.57317500	-2.49257500	-0.70709900
O	1.76083200	-3.85754100	-0.21739200
O	0.49194500	-2.22933000	-1.66954100
O	1.46949200	-1.50543600	0.46686600
N	-1.34023100	0.99504400	0.28010700
C	-2.67502900	0.74024700	0.06903200
C	-3.30249200	-0.50922600	-0.07651500
C	-4.65878900	-0.50186400	-0.44114100
H	-5.13980600	-1.47322900	-0.55577300
C	-5.40158600	0.67171900	-0.62034000
C	-4.75079100	1.91196300	-0.43888500
H	-5.29640000	2.84771800	-0.58226000
C	-3.41079000	1.94487700	-0.10243000
C	-2.43984900	3.01433200	0.05505700
C	-2.51418100	4.38970700	-0.06040300
H	-3.47887100	4.86396300	-0.25622500
C	-1.34502900	5.17051700	0.05541400
C	-0.12891200	4.51252200	0.27582700
H	0.78898400	5.09173300	0.37609100
C	-0.01296500	3.11850400	0.40794200
C	-1.19540500	2.36513200	0.29483800
C	-2.58739200	-1.77686600	0.14250500
C	-1.94415200	-2.02138300	1.37036300
H	-2.02364400	-1.26511400	2.15396800
C	-1.35572200	-3.26700100	1.63110500
C	-1.34630700	-4.20625100	0.59685900
H	-0.83961500	-5.15753000	0.76542300
C	-1.96384900	-3.99096300	-0.64222900
C	-2.60516600	-2.76914000	-0.84218300
H	-3.10010000	-2.54590500	-1.78682000
C	-0.77129100	-3.63907000	2.99726000
C	-1.06613200	-2.56806400	4.04991500
H	-2.14910600	-2.43000700	4.19565200
H	-0.62991400	-1.59463700	3.78740200
H	-0.63295400	-2.86676800	5.01676400
C	0.74639800	-3.82896800	2.86904800
H	1.18118100	-4.09671200	3.84600100
H	1.22911100	-2.91559300	2.49882000
H	0.99739900	-4.61533600	2.14376100
C	-1.41257900	-4.95060500	3.47894700
H	-1.02497200	-5.21624100	4.47549900
H	-1.18977900	-5.79019300	2.80483100
H	-2.50752800	-4.85541500	3.55036300
C	-1.92542100	-5.08729000	-1.70913400
C	-0.46746400	-5.36328500	-2.10524400
H	0.15135200	-5.64616600	-1.24226300

H	-0.00959300	-4.46107000	-2.53300300
H	-0.42783300	-6.17831000	-2.84626800
C	-2.56169300	-6.36399700	-1.13690600
H	-2.01504100	-6.73609800	-0.25773400
H	-2.55425700	-7.16429500	-1.89424100
H	-3.60617100	-6.18706300	-0.83477800
C	-2.69796800	-4.68573300	-2.96718500
H	-3.76177700	-4.49298400	-2.75529700
H	-2.64955000	-5.49987000	-3.70655900
H	-2.26632300	-3.78767500	-3.43442500
C	-6.88421800	0.65430200	-0.99927300
C	-7.69438400	1.35938200	0.10034900
H	-8.76599100	1.36170400	-0.15577100
H	-7.38211700	2.40582400	0.23492900
H	-7.57404600	0.84616100	1.06679500
C	-7.07065000	1.39599600	-2.33299300
H	-8.13307400	1.39586500	-2.62477800
H	-6.49443200	0.91106100	-3.13588400
H	-6.74326200	2.44455800	-2.27126600
C	-7.42561900	-0.76759800	-1.16073500
H	-7.34650300	-1.34485900	-0.22694000
H	-6.89703800	-1.31936600	-1.95304800
H	-8.49029400	-0.72978000	-1.43728900
C	-1.44487300	6.69285600	-0.06594500
C	-2.37686900	7.22566200	1.03413600
H	-3.38940000	6.80232500	0.95574800
H	-2.46705100	8.32140500	0.96224700
H	-1.98637200	6.97855900	2.03330900
C	-2.01901400	7.05154900	-1.44615600
H	-3.02240900	6.62683700	-1.59841600
H	-1.36971300	6.67541200	-2.25167300
H	-2.10083600	8.14500000	-1.55365700
C	-0.08459100	7.37777700	0.08135100
H	0.36984100	7.17951400	1.06408100
H	-0.20575200	8.46760800	-0.01353400
H	0.62311600	7.05476400	-0.69753700
C	1.30820300	2.50163500	0.61945100
C	2.37383600	2.85072700	-0.21262800
H	2.18364600	3.54707000	-1.03218300
C	3.65144500	2.31221700	-0.02494500
C	3.83207000	1.42504300	1.03910800
H	4.82000600	0.99993600	1.21064900
C	2.79793600	1.06449000	1.91195200
C	1.52590000	1.59643300	1.67506800
H	0.70302800	1.40367200	2.36672700
C	4.79524100	2.73275600	-0.95198800
C	5.09181300	4.22229100	-0.71659400
H	5.39179400	4.40418800	0.32712500
H	4.21095000	4.84800500	-0.92674900
H	5.90980200	4.56054100	-1.37312200
C	4.38307900	2.51410100	-2.41478700
H	4.13744700	1.45756900	-2.59484300
H	5.20766900	2.79753500	-3.08867000
H	3.50766000	3.11862400	-2.69392900
C	6.07096500	1.92929400	-0.68947700
H	6.46228400	2.09612200	0.32576200
H	6.85633800	2.23959800	-1.39603900
H	5.90101500	0.84995200	-0.82372500
C	3.10371600	0.16793600	3.11352800
C	3.96633700	0.96606300	4.10395600
H	4.19891600	0.35301100	4.98968700
H	3.44392700	1.87466400	4.44219200

H	4.91887600	1.27619800	3.64758100
C	3.85928400	-1.09139800	2.66444400
H	4.05792500	-1.73984100	3.53227900
H	4.82971800	-0.85070700	2.20527700
H	3.25846400	-1.64800700	1.93287900
C	1.82107000	-0.27399200	3.81426200
H	2.05179400	-0.98125600	4.62479400
H	1.15790400	-0.78974900	3.10573300
H	1.27338800	0.57116100	4.25918500
C	3.09853200	-2.02715400	-1.50109900
C	4.31458400	-2.33747100	-0.89425200
C	3.06418800	-1.38381800	-2.73428900
C	5.50158300	-1.97171000	-1.52055600
H	4.32400400	-2.87790300	0.05429800
C	4.26125700	-1.04138300	-3.35892600
H	2.09721000	-1.17541600	-3.19644600
C	5.49663600	-1.31665700	-2.76056000
H	6.45641000	-2.21367500	-1.04500600
H	4.23552500	-0.54902000	-4.33524400
C	6.77848700	-0.89242200	-3.41564300
H	7.62147000	-1.52897900	-3.11037300
H	6.70209500	-0.92345200	-4.51231000
H	7.03074100	0.14433600	-3.13448600

4.5.11 $RCuO_2C_2F_3$

0 2

Cu	-0.45335800	0.04620700	0.41446300
O	-1.96650600	0.34223000	-1.99441500
O	-2.34793600	0.05461300	0.19552200
N	1.42966500	-0.02837500	0.28113900
C	2.29766900	1.03480700	0.26315400
C	1.96509600	2.39697600	0.19858900
C	3.01807000	3.30629100	0.02868100
H	2.75693400	4.36309500	-0.02934100
C	4.36184600	2.90920500	-0.03794700
C	4.66622000	1.53197800	0.04623900
H	5.70292400	1.19395600	-0.02333500
C	3.65063900	0.60350700	0.18942200
C	3.58773800	-0.85212200	0.19056400
C	4.52024900	-1.86667900	0.06103700
H	5.58343900	-1.62163600	0.00208600
C	4.09585000	-3.21032400	-0.02173300
C	2.71873700	-3.48506400	0.01468500
H	2.36463100	-4.51293500	-0.06471100
C	1.74863200	-2.48584500	0.15861100
C	2.20219800	-1.16345800	0.25177300
C	0.55279000	2.80845400	0.28080400
C	-0.20874600	2.43935800	1.40292600
H	0.30204100	1.93911100	2.22825600
C	-1.55913000	2.80614000	1.51158100
C	-2.12843500	3.49198700	0.43812800
H	-3.18687300	3.74896800	0.48509100
C	-1.40651000	3.84616900	-0.71222000
C	-0.05054000	3.51726900	-0.76261300
H	0.55343700	3.75896100	-1.63686800
C	-2.36204500	2.50130200	2.77757100
C	-1.78348800	1.28858100	3.51240900
H	-0.78260200	1.48226200	3.92639200
H	-1.71975300	0.42291400	2.83583700
H	-2.43335100	1.00756000	4.35468700

C	-3.82816200	2.20091400	2.44458600
H	-4.36527500	1.90172500	3.35782000
H	-3.90066800	1.38959400	1.70829000
H	-4.35120100	3.07909500	2.03835600
C	-2.28365300	3.73248500	3.69443800
H	-2.84670000	3.55662500	4.62549900
H	-2.70872700	4.61907500	3.19915500
H	-1.24058600	3.96385500	3.96148600
C	-2.12944500	4.53485500	-1.87238900
C	-3.22091600	3.59112900	-2.40331300
H	-3.97227300	3.36078200	-1.63412300
H	-2.78907000	2.63649100	-2.73988300
H	-3.74505600	4.05775000	-3.25278900
C	-2.76569100	5.84114500	-1.37353900
H	-3.49826100	5.66231900	-0.57248500
H	-3.29405200	6.34554200	-2.19815600
H	-2.00086000	6.53113900	-0.98378600
C	-1.17863000	4.86808900	-3.02365500
H	-0.37093100	5.54676600	-2.70731200
H	-1.73565200	5.36891100	-3.83007200
H	-0.72514600	3.96074900	-3.45054600
C	5.50485200	3.91476500	-0.20041300
C	6.45291000	3.79990000	1.00409400
H	7.28612800	4.51392400	0.90482700
H	6.88655500	2.79253600	1.09202900
H	5.92278800	4.02022800	1.94344100
C	6.27392800	3.59946600	-1.49379900
H	7.10195300	4.31348100	-1.62956800
H	5.61271100	3.67025100	-2.37108500
H	6.70648500	2.58799200	-1.48069200
C	5.00215200	5.35766300	-0.28123800
H	4.46284800	5.65561500	0.63088100
H	4.33464300	5.51120400	-1.14293700
H	5.85631900	6.04174800	-0.39881400
C	5.14630900	-4.31567100	-0.16072900
C	6.08111300	-4.27774400	1.05895800
H	6.60133100	-3.31240100	1.14865400
H	6.84871300	-5.06398500	0.97767600
H	5.51818400	-4.44334600	1.99043100
C	5.96182000	-4.07985300	-1.44246500
H	6.48339700	-3.11130500	-1.42728000
H	5.31144600	-4.09687400	-2.33049000
H	6.72429700	-4.86638500	-1.56043900
C	4.51754400	-5.70827500	-0.24162000
H	3.93595000	-5.94831300	0.66152500
H	5.30887600	-6.46726300	-0.33737200
H	3.85554700	-5.80910500	-1.11521300
C	0.29649900	-2.77547500	0.14846600
C	-0.31849200	-3.18150500	-1.03411400
H	0.29481100	-3.27222100	-1.93308600
C	-1.69690200	-3.43310600	-1.08542300
C	-2.42117000	-3.31380400	0.10177500
H	-3.49132900	-3.51628400	0.08768500
C	-1.83387200	-2.94328300	1.32083000
C	-0.47115100	-2.63176500	1.31997300
H	0.04014900	-2.33990600	2.23864500
C	-2.33925500	-3.84423100	-2.41355800
C	-1.73862200	-5.18931500	-2.85264500
H	-1.93501800	-5.97281700	-2.10398900
H	-0.64908400	-5.12345600	-2.99349400
H	-2.18051700	-5.51068700	-3.80948800
C	-2.04704300	-2.77666200	-3.48018800

H	-2.43927700	-1.79160500	-3.18912700
H	-2.50757400	-3.06729500	-4.43794800
H	-0.96761800	-2.65577000	-3.65464800
C	-3.85641600	-4.00501700	-2.29511700
H	-4.13186400	-4.79681800	-1.58104300
H	-4.27492100	-4.28556200	-3.27368100
H	-4.34103200	-3.06953100	-1.98035200
C	-2.68891900	-2.92654700	2.59096900
C	-3.25300400	-4.33830800	2.82113400
H	-3.86663700	-4.35829000	3.73610600
H	-2.44241500	-5.07503800	2.93507700
H	-3.88990600	-4.66482400	1.98581400
C	-3.84780400	-1.93259800	2.42546700
H	-4.45602100	-1.90218400	3.34375300
H	-4.50839300	-2.21007400	1.59204100
H	-3.47523200	-0.92309800	2.20885700
C	-1.87324500	-2.53777400	3.82577500
H	-2.52232600	-2.53692300	4.71470100
H	-1.44229500	-1.53061200	3.73464700
H	-1.05317100	-3.24796400	4.01409900
C	-2.68636600	0.19209600	-1.02368100
C	-4.21963200	0.15423200	-1.22091200
F	-4.81137000	1.17431100	-0.58289900
F	-4.55761200	0.22655100	-2.50079800
F	-4.74358000	-0.97662300	-0.72540300

4.5.12 RCuN(SiMe$_3$)$_2$

0 2

Cu	-0.28126700	-0.46822700	-0.34417300
N	-0.02924000	1.14384700	0.61085300
C	-1.17001600	1.92343500	0.69078600
C	-2.50846700	1.49885900	0.79929000
C	-3.49520200	2.49329400	0.80483800
H	-4.52874100	2.16131800	0.90400400
C	-3.21517600	3.86643000	0.71276800
C	-1.87242200	4.26246000	0.60285000
H	-1.61101500	5.32040800	0.51997700
C	-0.86676400	3.30621400	0.58608300
C	0.57069200	3.37301800	0.42056100
C	1.44749200	4.42300100	0.18831600
H	1.06347500	5.44569700	0.15144100
C	2.81172800	4.16458700	-0.01868100
C	3.24454700	2.83145300	0.04676100
H	4.30414600	2.60215500	-0.06952700
C	2.39308600	1.74559300	0.30248600
C	1.02640100	2.02608900	0.46418400
C	-2.88717600	0.06911900	0.86041000
C	-2.22211900	-0.81901200	1.72039100
H	-1.41932300	-0.42459900	2.34271200
C	-2.61632100	-2.15837300	1.81991100
C	-3.67285700	-2.59228600	1.01097800
H	-3.99365300	-3.63370600	1.08388600
C	-4.34591600	-1.74368400	0.12479700
C	-3.93912200	-0.40885400	0.06927100
H	-4.43083800	0.29040900	-0.60679600
C	-1.98316000	-3.12234200	2.82792200
C	-0.73446400	-2.51909100	3.47004600
H	-0.96668000	-1.63085200	4.07670900
H	0.00296800	-2.22482000	2.70777300
H	-0.25463900	-3.25419800	4.13354100

C	-1.58072800	-4.43634700	2.14344000
H	-1.13625900	-5.12477000	2.87945300
H	-0.83979600	-4.26241600	1.35037900
H	-2.44000500	-4.95108800	1.68952200
C	-3.01453800	-3.41621200	3.92887300
H	-2.58961800	-4.09746600	4.68383000
H	-3.91886900	-3.88901000	3.51635600
H	-3.32257500	-2.48904900	4.43633100
C	-5.50466500	-2.28590900	-0.71804100
C	-5.03664700	-3.50752000	-1.52191400
H	-4.68334100	-4.32184000	-0.87260500
H	-4.20956300	-3.24197400	-2.19654300
H	-5.86470000	-3.90350100	-2.13101500
C	-6.64772800	-2.69682900	0.22343200
H	-6.32940500	-3.47689400	0.93144100
H	-7.49895800	-3.09303500	-0.35344400
H	-7.00190900	-1.83555500	0.81082500
C	-6.03537800	-1.24185600	-1.70321900
H	-6.44301000	-0.35860000	-1.18810400
H	-6.84890500	-1.67655600	-2.30384600
H	-5.25143300	-0.90352800	-2.39854700
C	-4.31725700	4.92998100	0.72312900
C	-4.10083400	5.87401500	1.91634000
H	-4.88115200	6.65193900	1.93896300
H	-3.12559300	6.38075700	1.86560800
H	-4.14082200	5.31980600	2.86676300
C	-4.25414300	5.73266700	-0.58624100
H	-5.03962700	6.50550900	-0.60263000
H	-4.40096500	5.07436900	-1.45643000
H	-3.28544100	6.23918600	-0.71039200
C	-5.71479500	4.31811800	0.84234900
H	-5.83400100	3.74795300	1.77641000
H	-5.94145900	3.64844400	-0.00158600
H	-6.47176500	5.11740100	0.84311100
C	3.76987800	5.32767600	-0.29269700
C	3.74312800	6.29913200	0.89773100
H	2.73637000	6.70857500	1.06916000
H	4.42307800	7.14780100	0.71861300
H	4.06076800	5.79386800	1.82285600
C	3.31939800	6.06228700	-1.56543000
H	2.30030200	6.46509400	-1.46704100
H	3.32871200	5.38463900	-2.43308700
H	3.99310900	6.90721500	-1.78183600
C	5.21149300	4.85664000	-0.49644100
H	5.60602700	4.34738300	0.39611300
H	5.86080500	5.72246000	-0.69820300
H	5.29987500	4.16912600	-1.35170800
C	2.99245300	0.40047000	0.45451300
C	3.83656900	-0.10760900	-0.53315200
H	3.92307100	0.44444800	-1.47212300
C	4.57537500	-1.28181200	-0.33074700
C	4.44807800	-1.92065000	0.90265000
H	5.02730800	-2.82403700	1.09239100
C	3.60225100	-1.44646300	1.91975600
C	2.85453800	-0.29721600	1.66637200
H	2.19401100	0.12357000	2.42482500
C	5.49026500	-1.80104700	-1.44399500
C	6.50905000	-0.71225400	-1.81453100
H	7.12469600	-0.43801200	-0.94379200
H	6.01857000	0.20190100	-2.18041000
H	7.18090800	-1.07044500	-2.61101400
C	4.63506800	-2.14929800	-2.67087700

H	3.91960500	-2.94926600	-2.42966500
H	5.27091800	-2.49831700	-3.50046200
H	4.05853000	-1.28314600	-3.02938800
C	6.25868200	-3.05527900	-1.02308600
H	6.90644700	-2.86590000	-0.15316600
H	6.90269000	-3.39234900	-1.84978000
H	5.57981800	-3.88534200	-0.77377300
C	3.56971100	-2.18052100	3.26502600
C	4.99812100	-2.27728400	3.82495500
H	4.98991800	-2.78244200	4.80414400
H	5.43566300	-1.27597700	3.95950600
H	5.66515100	-2.84935900	3.16340800
C	3.00702500	-3.59521500	3.06005400
H	2.98831000	-4.14492900	4.01510300
H	3.61679200	-4.17416800	2.35009600
H	1.98247300	-3.56081200	2.66348300
C	2.71196900	-1.44705400	4.29814200
H	2.71306400	-2.00426300	5.24763600
H	1.66678500	-1.34988200	3.97483900
H	3.10148700	-0.43786000	4.50199800
N	-0.39567400	-1.47289500	-1.88828500
Si	-0.95541000	-0.58098700	-3.28340300
Si	0.19766800	-3.11284700	-1.85478700
C	-1.77416400	1.02628000	-2.74624100
H	-2.62252800	0.84134900	-2.07045700
H	-1.06463400	1.68109500	-2.21435700
H	-2.14896100	1.58176100	-3.62125900
C	0.51667000	-0.12921000	-4.37608600
H	0.20870600	0.52631200	-5.20690100
H	1.26848300	0.40842400	-3.77574900
H	0.99800900	-1.02189200	-4.80282300
C	-2.20080700	-1.59459100	-4.27527300
H	-1.76753700	-2.54084100	-4.63528900
H	-3.07495900	-1.83863700	-3.65116000
H	-2.55480700	-1.03060400	-5.15321200
C	-1.28021900	-4.27104200	-1.66660800
H	-1.92187500	-3.93053000	-0.84039700
H	-1.88876200	-4.27417200	-2.58420700
H	-0.96026300	-5.30620700	-1.46397500
C	1.33184000	-3.28544700	-0.36604500
H	1.69599500	-4.31590100	-0.22698900
H	2.20430900	-2.62135600	-0.45855600
H	0.80063000	-2.99174600	0.55354300
C	1.12652700	-3.60520900	-3.42151100
H	1.52370900	-4.62916500	-3.32749500
H	0.46501500	-3.58616000	-4.30217500
H	1.97152300	-2.92939300	-3.61871100

4.5.13 RCuO^{t}Bu

0 2

Cu	-0.01058400	-0.80356600	0.21100400
O	-0.12628400	-2.56287200	-0.18955500
N	0.03837100	1.07758300	0.27878500
C	-1.04788900	1.93182100	0.30696000
C	-2.41770400	1.62358100	0.22789000
C	-3.31915600	2.69759000	0.16695700
H	-4.37781600	2.44323400	0.10981900
C	-2.92804800	4.04338600	0.20080200
C	-1.55391500	4.32668200	0.27585700
H	-1.20020000	5.36080300	0.28214600

C	-0.63231500	3.29189000	0.32062200
C	0.81449500	3.25732500	0.29990700
C	1.78413100	4.24560800	0.22537700
H	1.48189100	5.29589400	0.23658800
C	3.14053500	3.89501500	0.11626400
C	3.46581800	2.53171500	0.08783400
H	4.51009900	2.22626900	0.01806500
C	2.51479800	1.50345700	0.17889500
C	1.16353100	1.87936500	0.28001500
C	-2.93176200	0.24191800	0.16736400
C	-2.62957100	-0.69124400	1.16642400
H	-2.01789100	-0.36562900	2.01187200
C	-3.20075200	-1.97556200	1.15460800
C	-4.03393500	-2.30305500	0.08743800
H	-4.47627900	-3.29860400	0.05377500
C	-4.33977400	-1.40331200	-0.94756600
C	-3.78701400	-0.12696200	-0.88322100
H	-3.98981900	0.61215300	-1.65817800
C	-2.98243900	-2.93574400	2.32609100
C	-1.53439200	-2.85638600	2.81495400
H	-1.30399600	-1.87665900	3.26040500
H	-0.84648300	-3.03530700	1.97577500
H	-1.34927100	-3.60955400	3.59657400
C	-3.26775600	-4.38899400	1.93557500
H	-3.04206000	-5.05144500	2.78510800
H	-2.64221400	-4.70043900	1.08581900
H	-4.32360300	-4.55353300	1.67268400
C	-3.93696500	-2.52161300	3.45761900
H	-3.80432500	-3.17761000	4.33346600
H	-4.98741200	-2.58736100	3.13369300
H	-3.74820000	-1.48463400	3.77607600
C	-5.26315100	-1.84974800	-2.08469500
C	-4.67183000	-3.09248400	-2.76811500
H	-4.55716900	-3.93312200	-2.06795200
H	-3.67968100	-2.87527500	-3.19116700
H	-5.32950700	-3.42735600	-3.58609400
C	-6.64310000	-2.19093500	-1.50055500
H	-6.58392700	-3.00247300	-0.75993800
H	-7.32856000	-2.51621800	-2.29965800
H	-7.08687600	-1.31448600	-1.00334700
C	-5.44032500	-0.75881700	-3.14254500
H	-5.89803600	0.14990000	-2.72226400
H	-6.10178100	-1.12133900	-3.94426400
H	-4.48031800	-0.47979500	-3.60345200
C	-3.93629400	5.19517900	0.15340800
C	-3.79692800	6.04121400	1.42881800
H	-4.51044400	6.88098500	1.41443500
H	-2.78610700	6.46364100	1.53108000
H	-3.99839700	5.43485100	2.32534200
C	-3.64845900	6.06971300	-1.07731600
H	-4.36223000	6.90772700	-1.12923700
H	-3.73915100	5.48354900	-2.00482100
H	-2.63504600	6.49706700	-1.04949400
C	-5.38065800	4.69850900	0.06103200
H	-5.65904200	4.08491000	0.93147800
H	-5.55080700	4.10260300	-0.84886900
H	-6.06804300	5.55770300	0.02764200
C	4.20182100	4.99613100	0.03395100
C	4.13636200	5.85774300	1.30484400
H	3.15039300	6.33017300	1.42858100
H	4.88951300	6.66128900	1.26507000
H	4.33069500	5.24908000	2.20138300

C	3.92515600	5.87412700	-1.19688300
H	2.93432100	6.34976500	-1.14752700
H	3.96412400	5.27727300	-2.12118200
H	4.67664800	6.67646900	-1.27400300
C	5.61772900	4.42935500	-0.09031100
H	5.88917300	3.81111300	0.77903800
H	6.34452200	5.25397600	-0.15028400
H	5.73529000	3.81756000	-0.99800700
C	2.95674500	0.09739000	0.15782300
C	3.82949400	-0.34710200	-0.83795800
H	4.10720900	0.35219400	-1.62976800
C	4.33519300	-1.65190200	-0.83593700
C	3.95462900	-2.49392700	0.21233200
H	4.34261200	-3.51157100	0.24091700
C	3.09187600	-2.08751300	1.23793200
C	2.57436200	-0.78915200	1.18208800
H	1.95490100	-0.39968200	1.99260800
C	5.29210200	-2.09533300	-1.94688500
C	6.58051800	-1.26329000	-1.84497000
H	7.06709000	-1.41075700	-0.86833300
H	6.37899800	-0.18755300	-1.96045300
H	7.29304200	-1.55843500	-2.63211600
C	4.63985300	-1.86448100	-3.31872400
H	3.71405000	-2.45038600	-3.41842800
H	5.32826200	-2.16990200	-4.12282900
H	4.38707800	-0.80665700	-3.48292400
C	5.65744500	-3.57698600	-1.83564700
H	6.18354600	-3.80218400	-0.89531400
H	6.32761100	-3.85781600	-2.66249000
H	4.76534600	-4.21946800	-1.89568500
C	2.75846900	-3.06254600	2.36892500
C	4.05623500	-3.55388000	3.02747100
H	3.82289900	-4.25346400	3.84600600
H	4.63115100	-2.71387500	3.44804500
H	4.70610600	-4.08416600	2.31572200
C	1.98622500	-4.25219600	1.77606600
H	1.65545200	-4.93353200	2.57666600
H	2.61877400	-4.83193500	1.08566800
H	1.11179200	-3.89686800	1.20921600
C	1.90285300	-2.39808700	3.44734500
H	1.65881000	-3.12661200	4.23527600
H	0.95327700	-2.02979300	3.03853000
H	2.42824800	-1.55412100	3.92114600
C	-0.16680900	-3.05297200	-1.49449500
C	-1.00126100	-4.33648100	-1.47962800
H	-0.56718600	-5.05288500	-0.76551800
H	-2.02641800	-4.10480800	-1.15536300
H	-1.04273900	-4.81156200	-2.47257900
C	-0.82498500	-2.02269800	-2.42076900
H	-0.95998200	-2.41082200	-3.44334900
H	-1.80715200	-1.73736100	-2.01533300
H	-0.20290400	-1.11328000	-2.48661800
C	1.25547700	-3.34366100	-1.97863900
H	1.85536000	-2.42377400	-1.90969500
H	1.73118900	-4.09608400	-1.33195800
H	1.27823500	-3.70657100	-3.01913700

4.5.14 [RCu(THF)]$^{+}$

1 2

Cu	-0.06787200	-0.72699400	-0.11833400

N	0.10761900	1.13238300	0.20367300
C	-0.93214000	2.02731700	0.18078100
C	-2.30567300	1.74907000	0.05999300
C	-3.16512300	2.85167200	-0.06947800
H	-4.23142900	2.64291400	-0.15301800
C	-2.72025300	4.18031600	-0.05228900
C	-1.33229400	4.42613600	0.09111700
H	-0.95415100	5.45062500	0.09714600
C	-0.45614200	3.36772400	0.20213600
C	0.99564800	3.25830400	0.26630800
C	2.01995600	4.18003000	0.25491400
H	1.79215400	5.24805800	0.24768600
C	3.36541700	3.73771400	0.23552300
C	3.61253600	2.35936500	0.21993100
H	4.64122100	1.99998800	0.21915300
C	2.59728800	1.38869100	0.24057300
C	1.27037700	1.86017900	0.26552500
C	-2.85531100	0.37938200	0.09971600
C	-2.55488700	-0.47033400	1.17997400
H	-1.88092000	-0.10180500	1.95521900
C	-3.23130900	-1.68762000	1.34325000
C	-4.14608700	-2.06020100	0.35173300
H	-4.68233500	-3.00427900	0.46807900
C	-4.42888600	-1.26702900	-0.76868300
C	-3.77780400	-0.03426600	-0.86990200
H	-3.98524500	0.63758700	-1.70295300
C	-3.09270300	-2.54477600	2.60532800
C	-1.97368100	-2.04137900	3.51796700
H	-2.17750300	-1.03057700	3.90200500
H	-1.00325800	-2.02537000	3.00134100
H	-1.87144100	-2.70601400	4.38862900
C	-2.80504600	-4.00685600	2.23630300
H	-2.73273400	-4.62061200	3.14727800
H	-1.85308700	-4.10065100	1.69427000
H	-3.59838500	-4.44331600	1.61210900
C	-4.42168600	-2.46841300	3.37629700
H	-4.35916200	-3.05725000	4.30488500
H	-5.25945400	-2.86384700	2.78265400
H	-4.66182500	-1.42863100	3.64570600
C	-5.44244300	-1.75503000	-1.80826300
C	-4.93449100	-3.06329900	-2.43502900
H	-4.76404900	-3.84438400	-1.67832500
H	-3.99034600	-2.90148800	-2.97907800
H	-5.67086700	-3.45346400	-3.15462600
C	-6.79172100	-2.00590900	-1.11617700
H	-6.72183700	-2.78051400	-0.33824600
H	-7.53917500	-2.34256400	-1.85113900
H	-7.16857100	-1.08635800	-0.64302800
C	-5.65558800	-0.73413800	-2.92764600
H	-6.05053500	0.21874500	-2.54334400
H	-6.38640300	-1.12392200	-3.65166700
H	-4.72537400	-0.52651800	-3.47891400
C	-3.67701300	5.36523600	-0.17507600
C	-3.56262800	6.23197200	1.09023800
H	-4.24370400	7.09418700	1.01956200
H	-2.54522700	6.62562700	1.23324500
H	-3.83228500	5.65690100	1.98920100
C	-3.28610800	6.19611100	-1.40912100
H	-3.96540800	7.05616600	-1.51419600
H	-3.35266000	5.59460100	-2.32862900
H	-2.26223200	6.59211800	-1.33717600
C	-5.13319000	4.92312000	-0.33123500

H	-5.48451700	4.34771200	0.53890300
H	-5.28300700	4.31418100	-1.23610300
H	-5.77971000	5.80846100	-0.42111600
C	4.49005300	4.77169400	0.23328000
C	4.38059700	5.62865900	1.50570400
H	3.42092300	6.16356000	1.56445800
H	5.18167400	6.38383400	1.52186800
H	4.47805800	5.00837600	2.40974300
C	4.34269200	5.66713900	-1.00860300
H	3.38464700	6.20780600	-1.02099800
H	4.40908900	5.07469000	-1.93399400
H	5.14593300	6.41988800	-1.02716100
C	5.87561400	4.12391000	0.20139300
H	6.05430200	3.49008900	1.08341600
H	6.64853300	4.90652400	0.19822500
H	6.02351900	3.51336100	-0.70274700
C	2.94821400	-0.04475100	0.22585700
C	3.90306900	-0.51339700	-0.68184000
H	4.30634100	0.18531600	-1.41806200
C	4.34109500	-1.84168500	-0.66054000
C	3.80342600	-2.68816900	0.31627000
H	4.15344400	-3.71684500	0.36575400
C	2.83891800	-2.26358900	1.24105300
C	2.39898600	-0.93572900	1.16558200
H	1.70476400	-0.53966800	1.90993400
C	5.37314000	-2.31625100	-1.68720600
C	6.64383600	-1.46138800	-1.55936400
H	7.07778700	-1.54958400	-0.55166700
H	6.44632000	-0.39602400	-1.75028400
H	7.40090600	-1.79262800	-2.28697300
C	4.78268700	-2.15518700	-3.09736500
H	3.87877400	-2.77301300	-3.22043100
H	5.51316800	-2.47454500	-3.85687500
H	4.51124700	-1.11120800	-3.31438100
C	5.75489700	-3.78427100	-1.48740300
H	6.20335100	-3.96160900	-0.49798200
H	6.49770200	-4.08053900	-2.24278700
H	4.88831800	-4.45449500	-1.59996000
C	2.25944400	-3.23110100	2.27787400
C	3.23394900	-4.36977100	2.60058900
H	2.81858300	-4.99798900	3.40268200
H	4.20576900	-3.98277200	2.94276500
H	3.41093500	-5.02976800	1.73865400
C	0.97374200	-3.83223200	1.69163600
H	0.49135900	-4.50770600	2.41525700
H	1.18826300	-4.40576800	0.77625200
H	0.25443200	-3.04357000	1.42344000
C	1.93604200	-2.50292700	3.58861100
H	1.55166300	-3.21792800	4.33153100
H	1.16716200	-1.72697400	3.46595800
H	2.83424300	-2.02532800	4.00847400
C	-1.27622000	-3.18733300	-1.37129100
H	-1.42453000	-3.83195200	-0.49057300
H	-2.14850200	-2.52825100	-1.47025400
C	0.97847300	-2.74754000	-1.99314200
H	1.06652900	-2.00150000	-2.80094700
H	1.89755100	-2.73291700	-1.39293100
C	-0.93738800	-3.98244900	-2.61806300
H	-1.45847300	-4.94913200	-2.64939200
H	-1.21682200	-3.41910100	-3.52267000
C	0.58108500	-4.11138000	-2.51914300
H	1.06370200	-4.34751400	-3.47755000

H	0.85754400	-4.89623300	-1.79676400
O	-0.11524800	-2.36763200	-1.13426800

4.5.15 $Cu(NTf_2)_2^-$

-1 1

Cu	-0.00010100	0.00196800	-0.00132500
N	-1.90660200	0.00085600	0.00019600
N	1.90637500	0.00242700	-0.00251800
S	-2.68097800	0.21898300	-1.42727800
S	-2.67558100	-0.22353700	1.42971200
S	2.67803900	-1.42648600	-0.22121300
S	2.67820900	1.43083200	0.21893500
O	-4.03746400	-0.29175100	-1.44972500
O	-1.73173800	-0.09940000	-2.47967300
O	-4.03589800	0.27670200	1.45583000
O	-1.72605200	0.10210400	2.47965500
O	1.72764000	-2.47705700	0.09982200
O	4.03745700	1.45409300	-0.28439200
O	1.72978800	2.48210400	-0.10548900
O	4.03547000	-1.45088600	0.28688400
C	-2.82844900	2.07049000	-1.46851500
C	-2.80829200	-2.07622200	1.47108700
C	2.82168200	-1.46917800	-2.07304800
C	2.81540500	1.47371700	2.07124300
F	-3.45686400	-2.43826000	2.57054500
F	-3.46757100	-2.52099000	0.41592000
F	-1.60059400	-2.61314400	1.48614100
F	-1.62511900	2.61704900	-1.48785100
F	-3.48365600	2.42719900	-2.56578900
F	-3.48761100	2.51025300	-0.41118100
F	3.48085800	-0.41275000	-2.51493800
F	3.47515700	-2.56727900	-2.43033400
F	1.61718300	-1.48780900	-2.61704200
F	1.60904200	1.49052900	2.61114600
F	3.47469600	0.41829000	2.51535500
F	3.46601200	2.57281900	2.43072600

4.5.16 $Cu(NTf_2)_2$

0 2

Cu	0.00179100	0.01799200	0.01509200
N	-1.93548400	-0.07774700	-0.21649700
N	1.94127200	0.09337700	0.23377300
S	-2.14508700	-1.30527900	0.82078400
S	-3.12680900	0.91378100	-0.83195600
S	2.15828000	1.31662800	-0.80724700
S	3.11959200	-0.93296200	0.81631000
O	-3.19908300	-1.24994000	1.79554000
O	-0.70886400	-1.43963900	1.25375800
O	-4.40874400	0.26038700	-0.69585200
O	-2.63068100	1.47440100	-2.06286300
O	0.71789600	1.48051900	-1.21552600
O	4.41529500	-0.31405300	0.65296100
O	2.63766300	-1.48709500	2.05571100
O	3.19362600	1.23946400	-1.80046000
C	-2.42582100	-2.78590200	-0.27596700

C	-3.04470900	2.27228200	0.43832600
C	2.49349500	2.78860000	0.28561500
C	2.96969300	-2.28192200	-0.45835700
F	-3.85961400	3.24222600	0.09079000
F	-3.38367200	1.79201100	1.61763200
F	-1.80091700	2.72753200	0.49358800
F	-1.39255400	-2.89506900	-1.08890300
F	-2.52406500	-3.85543500	0.47877200
F	-3.52614500	-2.60622600	-0.96699200
F	3.59813300	2.57684300	0.96080100
F	2.61166300	3.85548800	-0.47004000
F	1.47564800	2.92633900	1.11297500
F	1.71087500	-2.69789600	-0.49088000
F	3.29933600	-1.80648800	-1.64223000
F	3.76010400	-3.27894500	-0.13203800

4.5.17 $Cu(N\{SiMe_3\}_2)_2$

0 2

Cu	-0.01683600	-0.00684900	0.26792400
N	1.80317600	0.07447700	0.21429200
N	-1.83291300	-0.05578000	0.10299200
Si	2.63884700	-1.45413700	0.42502500
Si	-2.60681300	1.48953600	0.41087800
C	-1.86550000	2.20012500	1.99167300
H	-2.25825500	3.20717500	2.20606900
H	-2.10752000	1.54910900	2.84701200
H	-0.76807600	2.27126200	1.93006300
C	-2.26201300	2.64654900	-1.03672900
H	-1.17928200	2.78013200	-1.18859900
H	-2.67897300	2.23123200	-1.96800800
H	-2.70863600	3.64083700	-0.87485300
C	-4.46883800	1.36082100	0.66237200
H	-4.72376100	0.58700800	1.40398200
H	-4.84908600	2.32362200	1.04118400
H	-5.00398900	1.13321100	-0.27102500
C	1.46616600	-2.68355900	1.24179100
H	1.09950300	-2.29010400	2.20386100
H	0.59068700	-2.90738800	0.61308200
H	1.97966000	-3.63772500	1.44273800
C	3.17975400	-2.12877400	-1.25024200
H	3.88653900	-1.44387300	-1.74533100
H	3.67394800	-3.10839300	-1.14828700
H	2.31005600	-2.25087100	-1.91542600
C	4.13377100	-1.27355800	1.55720100
H	4.53516900	-2.26943600	1.80625100
H	4.94282000	-0.68952300	1.09577200
H	3.84802300	-0.77857800	2.49880900
Si	-2.48121400	-1.59060800	-0.44809400
Si	2.44848900	1.56723100	-0.44873000
C	-2.62235300	-2.76476000	1.01721200
H	-2.97564300	-3.76019400	0.70303200
H	-1.65066700	-2.88700800	1.52011900
H	-3.33459600	-2.36786800	1.75808100
C	-1.29003800	-2.29235500	-1.73334000
H	-0.27350600	-2.42290700	-1.32999000
H	-1.63289200	-3.27075100	-2.10681700
H	-1.21650200	-1.60490700	-2.59130700
C	-4.16652800	-1.44043700	-1.27625300
H	-4.96644700	-1.20400600	-0.55947500
H	-4.16504900	-0.66541600	-2.05930300

H	-4.42020900	-2.39955600	-1.75673100
C	1.91158300	1.66514200	-2.25455300
H	0.81284800	1.62094200	-2.32971100
H	2.25227400	2.59777200	-2.73266100
H	2.31708300	0.81579000	-2.82682200
C	1.73257200	3.03719700	0.48359200
H	2.17233500	3.98163900	0.12473400
H	0.64206000	3.10596700	0.34863100
H	1.93417000	2.95395400	1.56319200
C	4.32741700	1.66642400	-0.36553900
H	4.68961100	1.67004100	0.67401600
H	4.81103900	0.83048800	-0.89498900
H	4.66536200	2.60087400	-0.84230400

4.5.18 $Cu(N\{SiMe_3\}Dipp)_2$

0 2

Cu	0.00001300	-0.00835500	0.00012000
N	1.64582600	-0.01293200	0.75889600
N	-1.64575800	-0.01282100	-0.75873100
C	-2.70210900	-0.01510600	0.17812300
C	-3.13615200	1.19783800	0.76775900
C	-3.28841400	-1.24469900	0.57040400
C	-4.16738200	1.15918700	1.71013600
C	-4.31733100	-1.23096000	1.51458200
C	-4.76590600	-0.04062300	2.07891800
H	-4.50658800	2.09143300	2.16926600
H	-4.77343200	-2.17468600	1.82340100
H	-5.57442000	-0.05012800	2.81375600
C	2.70205000	-0.01491300	-0.17809500
C	3.28856900	-1.24434200	-0.57056300
C	3.13575000	1.19817400	-0.76771100
C	4.31735600	-1.23030900	-1.51488000
C	4.16686300	1.15981700	-1.71022600
C	4.76560400	-0.03983300	-2.07918000
H	4.77360700	-2.17391300	-1.82384700
H	4.50580000	2.09217500	-2.16932900
H	5.57402100	-0.04910500	-2.81412600
Si	1.90681400	0.05748300	2.51106100
Si	-1.90654400	0.05768600	-2.51089700
C	-2.22614600	1.83104600	-3.06153800
H	-2.37200600	1.85772600	-4.15383400
H	-3.13418400	2.23748400	-2.58997100
H	-1.38369900	2.49523700	-2.81598900
C	-0.33410000	-0.57975300	-3.31475800
H	0.55946300	-0.06038500	-2.93033400
H	-0.20098500	-1.65561000	-3.12467700
H	-0.36386300	-0.42938100	-4.40561500
C	-3.40072600	-0.97267400	-3.00347500
H	-4.25516500	-0.74670700	-2.34538900
H	-3.69824800	-0.73799900	-4.03788200
H	-3.20349400	-2.05337800	-2.94383500
C	0.33443300	-0.57992400	3.31504700
H	-0.55914100	-0.06059100	2.93061100
H	0.20128600	-1.65579600	3.12509300
H	0.36422400	-0.42943500	4.40588800
C	3.40115700	-0.97285400	3.00320800
H	4.25511500	-0.74715500	2.34439600
H	3.69947900	-0.73781800	4.03729900
H	3.20382900	-2.05356900	2.94408000
C	2.22649800	1.83083000	3.06167000

H	2.37274000	1.85751400	4.15391300
H	3.13431600	2.23737400	2.58977100
H	1.38389800	2.49494200	2.81643100
C	-2.72704600	-2.55493300	0.05064300
H	-2.21736000	-2.33081600	-0.89813800
C	-2.43737000	2.50503200	0.44300400
H	-1.87140000	2.34029400	-0.48415700
C	-1.41345800	2.84459600	1.52802500
H	-1.89906300	2.98069200	2.50764800
H	-0.67158500	2.03725700	1.63401600
H	-0.86531600	3.76883300	1.28297800
C	-3.39840100	3.66488500	0.20095800
H	-2.84785100	4.55606800	-0.13915300
H	-4.14623900	3.41256100	-0.56631100
H	-3.94256200	3.94885300	1.11580400
C	-1.65379200	-3.08038200	1.00667600
H	-0.84118000	-2.34684300	1.12639400
H	-2.07706100	-3.27717400	2.00469000
H	-1.20831300	-4.01450500	0.62883000
C	-3.78917700	-3.61239600	-0.23032500
H	-3.33536200	-4.49246400	-0.71234400
H	-4.27457400	-3.96648600	0.69306500
H	-4.57508000	-3.22460500	-0.89607800
C	2.43675600	2.50521600	-0.44279800
H	1.87083600	2.34028800	0.48436200
C	2.72749800	-2.55473400	-0.05088600
H	2.21812300	-2.33085800	0.89811600
C	1.41278500	2.84475000	-1.52776500
H	0.67112900	2.03724900	-1.63397200
H	0.86440400	3.76879400	-1.28251400
H	1.89839000	2.98120200	-2.50733800
C	3.39761300	3.66519900	-0.20065000
H	3.94170300	3.94934600	-1.11548300
H	2.84692800	4.55625800	0.13956800
H	4.14550700	3.41291800	0.56657500
C	3.78979200	-3.61218700	0.22949600
H	3.33619200	-4.49236400	0.71151900
H	4.27486800	-3.96609100	-0.69413400
H	4.57591700	-3.22449300	0.89504300
C	1.65395000	-3.08003900	-1.00667700
H	0.84123000	-2.34654200	-1.12589700
H	2.07687000	-3.27652000	-2.00489900
H	1.20869500	-4.01430600	-0.62892400

4.5.19 Cu(N{Si^{i}Pr$_3$}Dipp)$_2$

0 2

Cu	-0.03569400	0.03163900	0.15300900
N	1.78370300	0.21320300	-0.01117500
N	-1.86138400	-0.20989200	0.14520100
C	-2.23591100	-1.52347100	-0.22259800
C	-2.13109400	-2.59865700	0.70800300
C	-2.64738500	-1.81749000	-1.55620100
C	-2.39604500	-3.90363900	0.28341800
C	-2.89387100	-3.14105600	-1.92677800
C	-2.76465900	-4.18794200	-1.02396600
H	-2.31410400	-4.72202600	1.00093100
H	-3.19298600	-3.35827600	-2.95372300
H	-2.95898700	-5.21693300	-1.33505200
C	2.29352500	1.51955900	-0.19837400
C	2.31980900	2.11652600	-1.48802700

C	2.76878700	2.26341800	0.91620200
C	2.79855900	3.42307100	-1.62443700
C	3.22878800	3.56770400	0.72565300
C	3.24677000	4.15576400	-0.53387200
H	2.82445100	3.87510700	-2.61951900
H	3.58539800	4.13780400	1.58632500
H	3.61370400	5.17649800	-0.66402700
Si	2.78003000	-1.26830500	0.01231700
Si	-2.86690400	1.24047500	0.42675400
C	-2.16496800	2.06389900	2.00674600
H	-1.11435300	2.20799800	1.69028300
C	-2.51595300	2.50780900	-0.98607900
H	-2.37993200	1.88505400	-1.88576900
C	-4.70109100	0.73178700	0.62949100
H	-4.63188800	-0.10540400	1.34778400
C	1.97067500	-2.39298400	1.32874000
H	0.91118700	-2.40550400	0.99964800
C	2.59730000	-2.11794700	-1.69213400
H	2.72520900	-1.28763300	-2.40576000
C	4.58772600	-0.80458500	0.42799700
H	4.48939800	-0.08784500	1.26202000
C	-2.77398800	-0.72445900	-2.59878000
H	-3.11975300	0.17367600	-2.06983100
C	-1.81540800	-2.35673600	2.17104900
H	-1.16031900	-1.46945800	2.21309600
C	-1.09125900	-3.51317000	2.85478000
H	-1.75600500	-4.37852100	3.00369000
H	-0.22018400	-3.85462600	2.27963900
H	-0.74612800	-3.20325000	3.85263700
C	-3.09861100	-2.04239800	2.94329900
H	-2.88346700	-1.81485900	3.99911900
H	-3.62977600	-1.18388400	2.51526300
H	-3.77962600	-2.90768600	2.90885400
C	-1.42371600	-0.38534300	-3.22241700
H	-0.70170600	-0.06716000	-2.45761300
H	-1.00164200	-1.25874100	-3.74328000
H	-1.52306100	0.43426000	-3.94974500
C	-3.80339100	-1.01280400	-3.68780800
H	-3.96646400	-0.10970200	-4.29614100
H	-3.46698700	-1.80495100	-4.37522300
H	-4.77276500	-1.31892400	-3.26693900
C	2.73884500	1.66910400	2.30988300
H	2.80300000	0.58078100	2.18544400
C	1.89625600	1.36524800	-2.73793400
H	1.42596100	0.42781800	-2.40264100
C	1.40236500	1.94420100	2.99317000
H	0.57567300	1.53059500	2.39874800
H	1.35986400	1.48145600	3.99208100
H	1.22618400	3.02607500	3.10528700
C	3.91057600	2.09166100	3.19006900
H	3.85410500	3.15344900	3.47778700
H	3.91372200	1.50615700	4.12279000
H	4.87476400	1.93271900	2.68257200
C	3.10735900	1.00813700	-3.60429500
H	2.80339600	0.38830600	-4.46306600
H	3.58598400	1.91862900	-3.99965100
H	3.87112700	0.45748000	-3.03942000
C	0.87216400	2.12742600	-3.57743500
H	-0.04205000	2.34153500	-3.01008800
H	1.27678200	3.08592000	-3.93846900
H	0.59147500	1.53733500	-4.46307400
C	-2.13758700	1.20314600	3.26378700

H	-3.15313000	0.96532500	3.61936800
H	-1.61088700	0.25405400	3.10054500
H	-1.62084900	1.72940600	4.08475500
C	-2.71501800	3.45510800	2.33209200
H	-3.70462200	3.39990000	2.80900700
H	-2.04770200	3.97182700	3.04314600
H	-2.81612300	4.10364500	1.45085500
C	-1.20093200	3.26542700	-0.78300500
H	-1.23438800	3.94605900	0.08011800
H	-0.33743000	2.59641900	-0.63336500
H	-0.96762800	3.88472300	-1.66489700
C	-3.66525000	3.47436700	-1.27930700
H	-3.87920900	4.14025100	-0.42826800
H	-3.41524600	4.12350500	-2.13619300
H	-4.60068900	2.95126600	-1.52781500
C	-5.39148000	0.17306000	-0.61540000
H	-5.39721600	0.88747900	-1.45437900
H	-4.91786400	-0.75409200	-0.96420900
H	-6.44533800	-0.06574400	-0.39124000
C	-5.55819600	1.81204700	1.29475600
H	-6.59256900	1.45107200	1.42653700
H	-5.18266400	2.08728900	2.29019100
H	-5.61359200	2.73364400	0.69486500
C	2.00155300	-1.81473000	2.74248500
H	1.54097400	-2.51018400	3.46269600
H	3.03064400	-1.62639000	3.09127200
H	1.44862200	-0.86627000	2.80556400
C	2.45897400	-3.84292200	1.31290600
H	1.89259600	-4.45128900	2.03896100
H	2.33543900	-4.31723500	0.32923200
H	3.52025300	-3.92859100	1.58912500
C	5.30717300	-0.06596100	-0.70220700
H	5.42182200	-0.69465800	-1.60021600
H	4.78033200	0.85303500	-0.99685200
H	6.32281300	0.22769100	-0.38558100
C	5.42598100	-1.97617200	0.94513900
H	6.45192200	-1.64154500	1.17661900
H	5.01142800	-2.40931500	1.86741700
H	5.51235900	-2.78971600	0.20760000
C	1.18308700	-2.66909400	-1.88211700
H	0.99039900	-3.55112000	-1.25147200
H	0.40482800	-1.92990900	-1.63668600
H	1.01229300	-2.97723800	-2.92758100
C	3.65882000	-3.16504400	-2.03242100
H	3.63813700	-4.02473500	-1.34512100
H	3.49515100	-3.56390000	-3.04860500
H	4.67738700	-2.75019900	-2.00725700

5 References

[1] A. Hinz, *Chem. Eur. J.* **2019**, *25*, 3267–3271.

[2] C. L. Wagner, L. Tao, E. J. Thompson, T. A. Stich, J. Guo, J. C. Fettinger, L. A. Berben, R. D. Britt, S. Nagase, P. P. Power, *Angew. Chem. Int. Ed.* **2016**, *55*, 10444–10447.

[3] G. R. Fulmer, A. J. M. Miller, N. H. Sherden, H. E. Gottlieb, A. Nudelman, B. M. Stoltz, J. E. Bercaw, K. I. Goldberg, *Organometallics* **2010**, *29*, 2176–2179.

[4] S. Stoll, A. Schweiger, *J. Magn. Reson.* **2006**, *178*, 42–55.

[5] G. M. Sheldrick, **1997**, SHELXS-97.

[6] G. M. Sheldrick, *Acta Crystallogr. A* **2015**, *71*, 3–8.

[7] G. M. Sheldrick, **2013**, SHELXL-2013.

[8] C. B. Hübschle, G. M. Sheldrick, B. Dittrich, *J. Appl. Crystallogr.* **2011**, *44*, 1281–1284.

[9] B. Ravel, M. Newville, *J. Synchrotron Rad* **2005**, *12*, 537–541.

[10] M. J. Frisch, G. W. Trucks, H. B. Schlegel, G. E. Scuseria, M. A. Robb, J. R. Cheeseman, G. Scalmani, V. Barone, B. Mennucci, G. A. Petersson, et al., *Gaussian 09, Revision D.01*, **2009**.

[11] E. D. Glendening, C. R. Landis, F. Weinhold, *J. Comput. Chem.* **2013**, *34*, 1429–1437.
